# Metformin May Alter the Metabolic Reprogramming in Cancer Cells by Disrupting the L-Arginine Metabolism: A Preliminary Computational Study

**DOI:** 10.3390/ijms24065316

**Published:** 2023-03-10

**Authors:** Bryan Alejandro Espinosa-Rodriguez, Daniela Treviño-Almaguer, Pilar Carranza-Rosales, Monica Azucena Ramirez-Cabrera, Karla Ramirez-Estrada, Eder Ubaldo Arredondo-Espinoza, Luis Fernando Mendez-Lopez, Isaias Balderas-Renteria

**Affiliations:** 1 Universidad Autonoma de Nuevo Leon, School of Chemistry, Laboratory of Molecular Pharmacology and Biological Models, San Nicolas de los Garza 64570, Mexico; bryan.espinosardr@uanl.edu.mx (B.A.E.-R.); daniela.trevinoal@uanl.edu.mx (D.T.-A.); monica.ramirezcbr@uanl.edu.mx (M.A.R.-C.); karla.ramirezst@uanl.edu.mx (K.R.-E.); eder.arredondosp@uanl.edu.mx (E.U.A.-E.); 2 Centro de Investigacion Biomedica del Noreste, Laboratory of Cell Biology, Instituto Mexicano del Seguro Social, Monterrey 66720, Mexico; carranza60@yahoo.com.mx; 3 Universidad Autonoma de Nuevo Leon, School of Public Health and Nutrition, Center for Research on Nutrition and Public Health, Monterrey 66460, Mexico

**Keywords:** metformin, L-arginine, cancer, creatine, biguanides, metabolism

## Abstract

Metabolic reprogramming in cancer is considered to be one of the most important hallmarks to drive proliferation, angiogenesis, and invasion. AMP-activated protein kinase activation is one of the established mechanisms for metformin’s anti-cancer actions. However, it has been suggested that metformin may exert antitumoral effects by the modulation of other master regulators of cellular energy. Here, based on structural and physicochemical criteria, we tested the hypothesis that metformin may act as an antagonist of L-arginine metabolism and other related metabolic pathways. First, we created a database containing different L-arginine-related metabolites and biguanides. After that, comparisons of structural and physicochemical properties were performed employing different cheminformatic tools. Finally, we performed molecular docking simulations using AutoDock 4.2 to compare the affinities and binding modes of biguanides and L-arginine-related metabolites against their corresponding targets. Our results showed that biguanides, especially metformin and buformin, exhibited a moderate-to-high similarity to the metabolites belonging to the urea cycle, polyamine metabolism, and creatine biosynthesis. The predicted affinities and binding modes for biguanides displayed good concordance with those obtained for some L-arginine-related metabolites, including L-arginine and creatine. In conclusion, metabolic reprogramming in cancer cells by metformin and biguanides may be also driven by metabolic disruption of L-arginine and structurally related compounds.

## 1. Introduction

Metabolic reprogramming is an adaptation mechanism implemented by cancer cells in response to their microenvironment, which alters their metabolism to sustain the energy request for growth and proliferation [1]. Usually, the tumor microenvironment is hypoxic, acidic, and low in nutrients; despite these conditions, the cells present an abnormal metabolism to maintain themselves and to keep growing, including alterations in the tricarboxylic acids (TCA) cycle, glycolysis, the urea cycle (UC), nitric oxide (NO) metabolism, polyamines biosynthesis, and protein, lipid, and nucleic acid biosynthesis [2]. Today, one of the most important emergent therapies with remarkable potential is to modulate the metabolic reprogramming of cancer cells [2].

L-Arginine plays a central axis due to its ability to be incorporated into the anabolic and catabolic pathways mentioned before. In non-cancer cells, L-arginine is usually derived from exogenous uptake from the diet and endogenous biosynthesis through UC intermediates [3]. Once it has been absorbed, L-arginine can be incorporated into protein synthesis, acting as a building block. Moreover, L-arginine is reported to positively regulate anabolism itself by upregulating mTORC1 activity through the modulation of CASTOR1 and SLC38A9, the arginine sensors of the cells [4]. In addition, L-arginine is the precursor of a plethora of substances needed for proliferation, immune system regulation, DNA repair, or regulation of gene expression, such as polyamines, NO, and creatine. L-Arginine is key in two polyamine-producing pathways: the first pathway is the decarboxylation of L-arginine to agmatine by arginine decarboxylase (ADC) and subsequent hydrolysis via agmatinase, culminating with putrescine production; the second pathway involves L-ornithine, which is derived from L-arginine hydrolysis via arginase (ARG) in the UC. In addition, L-arginine is also involved as a precursor in the nitric oxide synthase (NOS)-catalyzed NO production and in the protein arginine methyltransferase (PRMT)-dependent production of the dimethylated derivatives of L-arginine: asymmetric dimethylarginine (ADMA) and symmetric dimethylarginine (SDMA). There are three NOS isoforms: inducible (iNOS), endothelial (eNOS), and neural (nNOS); eNOS is involved in vascular tone regulation, but iNOS is normally overexpressed during inflammation. On the other hand, ADMA is considered to be a potent competitive inhibitor of NOS that leads to less NO production. In addition to this, in the kidneys, L-arginine serves as the precursor of guanidinoacetate (GAA) via arginine:glycine amidinotransferase (AGAT), which is transported to the liver and converted into creatine by guanidinoacetate N-methyltransferase (GAMT) [3]. Interconversion between creatine and phosphocreatine in the cells in the so-called phosphagen system can maintain the different ATP pools by phosphorylating ADP to ATP via different isoforms of creatine kinases (CK), such as the muscle-type and brain-type CK [5].

Cancer cells reprogram L-arginine metabolism to support the proliferation and progression of malignancy, suppressing the immune response, and regulating gene expression [2]. The absorption of L-arginine in cancer cells is achieved by the overexpression of cationic amino acid transporters [6]. Indeed, hyperactivation of mTORC1 by L-arginine sensors has been reported in several types of cancer and is associated with increased anabolic pathways, such as the biosynthesis of nucleotides, protein, and fatty acids, and with the suppression of autophagy [7]. In tumors, UC was reported to be disrupted at different points. For instance, ORNT1, OTC, ASS1, and ASL were commonly downregulated. Additionally, it was observed at a higher carbamoyl phosphate concentration and its consequent input in the pyrimidine biosynthesis by carbamoyl phosphate synthetase 2 (CPS2) [8]. These metabolic changes led to increases in cellular growth and survival. Additionally, increased polyamines concentrations are usually observed in cancer cells. Polyamines have been implicated in nucleic acids and protein synthesis, chromatin stabilization, the regulation of paracrine communication, and prevention against oxidative DNA damage [9]. It has been reported that cancer cells exhibit increased ARG expression and activity, leading to a high production of L-ornithine [10]. Some studies also reported the overexpression of ODC1 due to MYC activity with a subsequent elevation in the putrescine concentration [11,12]. These alterations contribute to cancer progression. Furthermore, cancer cells can restore energy by using the phosphagen system to allow increased proliferation and survival. Additionally, creatine plays a key role in immunity by modulating T cells and acting as an efficient energy buffering mechanism when the cells demand high levels of ATP due to the high phosphate transfer potential of phosphocreatine [13,14]. Although some studies have showed contradictory results about expression of CK isoforms, CK has been correlated with the cancer prognosis [14,15,16]. Regarding NO metabolism, it was reported that NO had a paradoxical dual activity, i.e., it promoted several hallmarks of cancer, such as apoptosis inhibition, epithelial-to-mesenchymal transition induction, and increased vascular infiltration and permeability, but at the same time, it was reported that NO counteracts the mechanisms mentioned before [17]. Further, decreased ADMA levels were reported in prostate cancer due to the upregulation of ADMA breakdown via dimethylarginine dimethylaminohydrolase (DDAH), leading to high NOS activity and angiogenesis [18].

Taking advantage of L-arginine dependency in some tumors, in arginine deprivation therapy (ADT), L-arginine levels are decreased by administering arginine-depleting agents, such as pegylated arginine deiminase or pegylated arginase 1 [19]. ADT has shown promising results due to the pivotal role of L-arginine and their related metabolites involved in cancer metabolism, e.g., NO, ADMA, polyamines, and creatine, although cancer sensitivity seems to be dependent on the urea cycle enzymes [19,20]. Hence, the modulation of metabolic reprogramming by deprivation therapies or other approaches seems a rational alternative to some specific cancer types, and more research is needed to improve its efficacy and application.

In pharmacology, the disruption of specific metabolic pathways has been performed using structurally related molecules that resemble endogenous ligands. For instance, statins imitate the structure of β-hydroxy-β-methylglutaryl-CoA (HMG-CoA), leading to the inhibition of HMG-CoA reductase and blocking cholesterol biosynthesis [21]. In line with this, there are several reports about metformin altering different metabolites related to L-arginine, suggesting the potential to interfere with UC, creatine biosynthesis, NO production, and polyamines metabolism [22].

Metformin, buformin, and phenformin are biguanides with anti-diabetic properties but nowadays, metformin is the only biguanide on the market and the first-line treatment for management of type 2 diabetes mellitus. Beyond their anti-diabetic properties, anticancer effects have been described in both in vitro and in vivo assays [23]. Despite it being such a versatile drug, the mechanism (or mechanisms) of action behind these effects remain elusive. Currently, the most established anti-cancer mechanism of action is the activation of AMP-activated protein kinase (AMPK) due to the energy deficit induced by the metformin-dependent inhibition of mitochondrial complex 1. In turn, AMPK activation negatively regulates those energy-consuming metabolic pathways, e.g., gluconeogenesis, glycogen synthesis, and fatty acid synthesis, but upregulates energy-generating metabolic pathways, e.g., glycolysis, glycogen breakdown, fatty acid oxidation, and autophagy [24].

**Figure 1:**
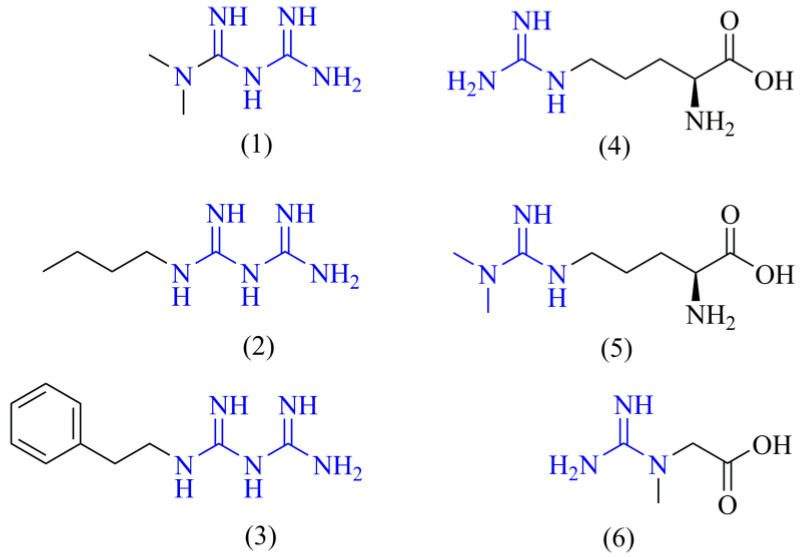
Chemical structures of biguanides and L-arginine-related metabolites. The chemical structures of the main biguanides, metformin (**1**), buformin (**2**), and phenformin (**3**), and three representative arginine-related metabolites such as L-arginine (**4**), ADMA (**5**), and creatine (**6**) are shown. The guanidine pharmacophore is highlighted in blue.

However, there are some inconsistencies about this previous description related to the simplicity of metformin’s chemical structure. With a small structure and the lack of directing groups compared to other complex drugs, it is virtually impossible that metformin could bind to only one target in the cell. Even the more complex drugs usually act through binding to different targets [25]. Molecular promiscuity appears to be a more logical approach to understand metformin’s anti-cancer mechanism of action. Given that the basic metformin’s pharmacophore is the guanidine group, it is reasonable to think that it may resemble guanidine-containing endogenous ligands inside the cell, e.g., L-arginine, creatine, ADMA, or some other related metabolites (Figure 1). Our hypothesis states that metformin (and biguanides in general) may be acting in the cancer cell as an antagonist of L-arginine and its related metabolites, leading to modulation of their corresponding targets, and, therefore, altering the metabolic reprogramming of cancer cells.

Computational tools can be employed to generate, transform, interpret, predict, and visualize data of a chemical and biological nature. These tools are commonly applied in drug discovery and exploration of the biological effects of molecules, allowing the prediction of physicochemical properties and drug-likeness, or even the prediction of possible targets according to the ligand structure [26]. In bioinformatics, molecular dynamics and molecular docking are commonly used to model the binding of ligands to specific targets, and to predict the affinities of ligands to targets of interest. However, molecular docking is normally used as a quick and inexpensive exploratory tool to generate preliminary insights to design further experiments [27]. On the other hand, given the computational power needed to run molecular dynamics simulations and the long waiting times needed to perform the thermodynamic calculations, it has been used as a more reliable computational technique to further validate the interactions observed in molecular docking simulations or experimental evidence [26]. In addition, it is common to use machine learning algorithms to extract relevant and significant information from the data [28]. In general, computational tools have demonstrated a high potential to generate, develop, and sustain hypotheses, as well as, to gain insights about the molecular actions of substances, as a first step before experiment design and performance [26]. For instance, exploration of the possible mechanism of action behind the arrest of the cell cycle in the sub-G1 phase in cancer cells led to the identification of possible interactions of geranyl farnesol, sahandinone, and 4-dehydrosalvilimbinol, a group of terpenoids present in *Salvia lachnocalyx*, with DNA topoisomerase I through a computational methodology centered on molecular docking and molecular dynamics simulations, suggesting that these terpenoids could be good candidates for the design of new drugs [29]. On the other hand, it has been reported that six possible targets genes (e.g., AR, HSP90AA1, MMP9, PGR, PTGS2, and TNF) are responsible for the action of the pseudo phosphorous stem of *Cremastra appendiculata* by employing a reverse network pharmacology approach that can allow for the in vitro or in vivo targeted study of molecular actions in the future [30]. The computational methodology they followed included tools for predicting the physicochemical properties and biological targets, such as SwissADME and SwissTargetPrediction, respectively, as well as tools to predict the binding and affinity of ligands in different targets of interest, such as molecular docking [30].

To test this hypothesis, in the present work, we employed different cheminformatic tools to seek for structural and physicochemical similarities between biguanides and different L-arginine-related metabolites by employing tools, such as the PubChem Score Matrix Service, SwissADME, and SwissTargetPrediction. Finally, we employed molecular docking, a bioinformatic technique, to test if the chemical similarity observed for biguanides may be translated into similar binding modes and affinities to those of L-arginine-related metabolites in their corresponding targets. Given that the complete metformin’s mechanism of action is still unknown, the relevance behind our hypothesis and this study is the establishment and support of a new possible theoretical framework to progress in the understanding of metformin’s biological actions in cancer cells, although this knowledge may be extrapolated to improve the comprehension of its therapeutic effects in other diseases where L-arginine, or its related metabolites, play a pivotal role.

## 2. Results

### 2.1. Database Creation

Before performing the different comparisons, we created a database containing the candidate metabolites, whose simplified molecular-input line-entry system (SMILES) and their compound ID (CID) were collected from PubChem. There were 20 candidate molecules in the final version of the constructed database, including 3 biguanides (metformin, buformin, and phenformin), 7 members of the UC (L-ornithine, L-citrulline, carbamoyl phosphate, L-aspartic acid, L-argininosuccinic acid, fumaric acid, and urea), L-arginine and their endogenous methylated derivatives (ADMA and SDMA), 5 members from the polyamine metabolism (agmatine, putrescine, spermine, spermidine, and cadaverine), and 2 members from creatine biosynthesis (guanidinoacetic acid and creatine).

### 2.2. Structural Comparison between Biguanides and L-arginine-Related Metabolites

After generating the database, we proceeded to assess the structural similarity between biguanides and the candidate metabolites by employing the PubChem Score Matrix Service to compare their chemical structures at two levels: the 2D level and 3D level. Selected results about the comparison of biguanides against candidate metabolites are shown in Table 1 for the 2D and 3D analyses, while the complete results can be observed in the Supplementary Material.

Tanimoto coefficients (TC) were generated from 2D structural comparisons. They ranged from 0 to 100 depending on the grade of the 2D structural similarity. The higher the TC value is, the more structurally similar the compared molecules are. Given metformin, buformin, and phenformin share the same pharmacophore, we expected to see high TC values (TC ≥ 65) between them. However, as shown in Table 1, a moderate similarity was observed only between metformin and buformin with a TC of 63, while phenformin showed low similarities with metformin (TC of 23) and buformin (TC of 33). Compared to those of the candidate metabolites, there were moderate similarities observed for buformin against different polyamines, e.g., cadaverine, spermidine, spermine, and putrescine, with TC values ranging from 51 to 62. It is noteworthy that buformin showed a high similarity with agmatine (TC of 88), but a moderate similarity against guanidinoacetic acid (TC of 50), an intermediate of creatine biosynthesis. On the other hand, metformin showed moderate similarity only with agmatine (TC of 56), and phenformin did not even reach or surpass a TC of 50 with any candidate metabolite.

Related to TC, Shape Tanimoto (ST) and Color Tanimoto (CT) were generated from 3D structural comparisons. They ranged from 0 to 100 depending on the grade of similarity in terms of shape or features (H-bonding donors or acceptors, rings, etc.), respectively. When ST and CT were summed up, combo T was generated, and it describes both the shape and features in only one parameter going from 0 to 200. Similar to the 2D structural comparisons, at the 3D level, the results suggested a moderate similarity between metformin and buformin with a combo T of 122, but phenformin still showed a low level of similarity with metformin (combo T of 89). Additionally, when phenformin was compared with buformin at the 3D level, its similarity was moderate (combo T of 111). Compared to the candidate metabolites, metformin showed moderate similarities against L-arginine, cadaverine, creatine, agmatine, and L-ornithine with combo T values going from 101 to 129. It is noteworthy that metformin showed higher similarities compared to those of guanidinoacetic acid and L-aspartic acid (combo T > 130). In the same way, buformin showed moderate similarities against L-arginine, agmatine, guanidinoacetic acid, creatine, and spermidine, with combo T values ranging from 102 to 120. Only phenformin showed lower similarities (combo T < 100) compared to those of all the candidate metabolites.

Remarkably, the moderate-to-high similarity observed for metformin or buformin compared to those of some candidate metabolites was due principally to their high level of shape resemblance with ST values > 80. On the other hand, the CT values tended to be less than 50 because of the high diversity of functional features of tested metabolites. However, the presence of the common guanidine moiety in L-arginine, agmatine, creatine, and guanidinoacetic acid led to the highest CT values observed for biguanides.

Finally, we performed another structural similarity assay employing the SwissSimilarity tool, aiming to reproduce the previous observations by seeking if there were a common scaffold between the biguanides and some candidate metabolites. As we anticipated in our hypothesis, the results showed that L-arginine, agmatine, creatine, spermidine, and cadaverine shared a common scaffold with metformin and buformin, but not phenformin (data not shown).

Considering only structural criteria, these results suggested moderate-to-high structural similarities between the biguanides, especially metformin and buformin, and some candidate metabolites, such as agmatine, intermediates from UC and, especially, creatine biosynthesis.

### 2.3. Physicochemical Comparison between Biguanides and L-arginine-Related Metabolites

The physicochemical comparisons between biguanides and the candidate metabolites began with the prediction of their physicochemical properties, employing the collected SMILES through the SwissADME tool [31]. The predictions were performed in ionized (pH 7.4) and non-ionized modalities. For each molecule, we collected seven physicochemical parameters, including molecular weight, consensus log P, topological polar surface area (TPSA), rotatable bonds, the number of hydrogen bond donors and acceptors, and the fraction of sp^3^ carbons. After the results were collected for each modality, the analysis of those parameters aiming to obtain the similarity under physicochemical criteria was performed. To identify physicochemical relationships between biguanides and the candidate metabolites, an unsupervised machine learning approach with the collected results was employed. First, we performed a principal component analysis (PCA), employing our variables as an exploratory analysis of the data. The PCA of ionized modality showed a close relationship of metformin and buformin with creatine, guanidinoacetate, and L-aspartate. In terms of the physicochemical properties, phenformin was isolated in the PCA. When it was performed in the non-ionized modality, the PCA showed that all the biguanides were similar to creatine, agmatine and L-ornithine in terms of physicochemical properties.

After that, we proceeded to perform hierarchical clustering (HC) using the different physicochemical profiles. First, preliminary hierarchical clustering including only candidate metabolites was performed. After that, each biguanide was compared, one at a time, against the different candidate metabolites in HC.

Without including any of the biguanides, the preliminary hierarchical clustering at both the ionized and non-ionized modalities generated a cluster for L-arginine, L-citrulline, ADMA, and SDMA, metabolites that belong to the urea cycle and NO metabolism. Polyamines were grouped in two clusters: one for low-molecular-weight polyamines (putrescine and cadaverine) and high-molecular-weight polyamines (spermidine and spermine). In the case of ionized modality, L-ornithine was grouped together with spermidine and spermine. With some differences, creatine, agmatine, guanidinoacetic acid, and L-aspartic acid were grouped together at both of the modalities, accounting for metabolites from the urea cycle, polyamines metabolism, and, mainly, creatine metabolism. L-Argininosuccinic acid alone and fumaric acid with urea (and carbamoyl phosphate in the ionized modality) are represented in the last two clusters.

**Figure 2:**
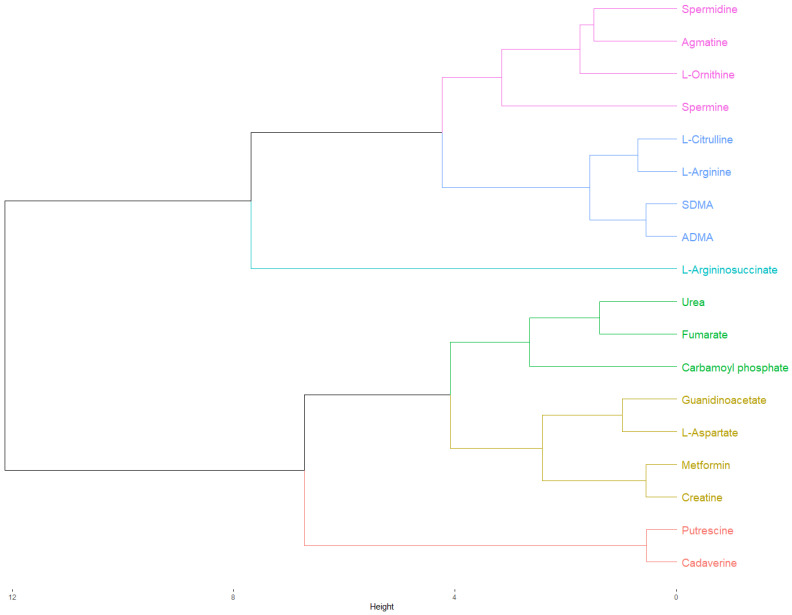
Hierarchical clustering of the physicochemical comparison between metformin and candidate metabolites in the ionized modality (pH 7.4). As highlighted in yellow, metformin and creatine were clustered together. After that, this cluster was grouped together with another cluster composed of L-aspartate and guanidinoacetate. The Euclidean distances from these clusters suggested a closer physicochemical relationship of metformin with creatine, L-aspartate, and guanidinoacetate, in that order, indicating a possible involvement of metformin in creatine biosynthesis and the urea cycle. The cluster of metformin was grouped with another cluster composed of urea, fumarate, and carbamoyl phosphate into a new cluster, although the Euclidean distance of this association was relatively large, suggesting a poor physicochemical relationship. Finally, this new cluster was grouped together with putrescine and cadaverine, the low-molecular-weight polyamines, but the Euclidean distance of this association was even larger. As expected, L-arginine was grouped with its precursor, L-citrulline, and their dimethylated derivatives, ADMA, and SDMA in the same cluster. The cluster of L-arginine were grouped together with another cluster composed of spermidine, agmatine, L-ornithine, and spermine, the precursors and final products of polyamine metabolism, into a new cluster. Finally, this cluster was grouped with L-argininosuccinate.

When metformin was included in the HC, this was grouped together with creatine, guanidinoacetic acid, and L-aspartic acid in both modalities, although in the non-ionized modality, L-ornithine and agmatine were also included (Figure 2). In the case of buformin, this was grouped together with agmatine, creatine, guanidinoacetic acid, and L-aspartic acid, although in the non-ionized modality, L-ornithine and carbamoyl phosphate were also included. Finally, phenformin was grouped with fumaric acid and urea, but carbamoyl phosphate was included in the ionized modality.

As can be observed, the physicochemical comparison agreed with the previous structural relationships obtained for metformin and buformin against creatine, guanidinoacetate, agmatine, and L-aspartate. Although phenformin showed a physicochemical relationship with agmatine, creatine, and L-ornithine during the PCA in the non-ionized modality, physiologically, non-ionized relationships are of little relevance because all the candidate metabolites and biguanides are ionized inside the cell. This is observed when the PCA was performed in the ionized modality, where phenformin was alone. PCA analyses and all the hierarchical clustering are shown in the Supplementary Material.

By limiting our analysis to both structural and physicochemical criteria, these results suggested a relationship between metformin and buformin, but not phenformin, with agmatine, L-aspartate, and especially, intermediates from creatine metabolism.

### 2.4. Structure-Based Target Prediction of Biguanides and L-arginine-Related Metabolites

Once we observed structural and physicochemical similarities of biguanides with some arginine-related metabolites, we were interested in testing if this similarity could be translated into an affinity for targets whose main substrates or ligands belong to the candidate metabolites included in the database that we tested before. To prove this assumption, we performed a structure-based target prediction employing the SwissTargetPrediction tool [32]. For each predicted target, its probability score, and its known actives 3D/2D were collected. We observed that biguanides, especially phenformin, were predicted to target the three different isoforms of nitric oxide synthase, whose main substrate is L-arginine, and its main inhibitor is ADMA. However, only the neural isoform of NOS was predicted with a high probability score, although the known actives 3D/2D parameter suggested all biguanides could target the inducible, endothelial, and neural isoforms. Furthermore, the known actives 3D/2D suggested that biguanides could also bind to several isoforms of carbonic anhydrases, whose known ligands are polyamines.

Indeed, these observations agree with our previous results of structural and physicochemical similarities that showed moderate-to-high similarities of biguanides to intermediates from polyamines metabolism, NO production, and UC. The complete predicted targets for biguanides can be found in the Supplementary Material.

### 2.5. Affinity Comparison between Biguanides and L-arginine-Related Metabolites

Following the target prediction in the SwissTargetPrediction tool, we proceeded to perform a molecular docking simulation to test if the affinities were comparable to those of candidate metabolites in their corresponding targets. The protein files required for the simulation were obtained from Protein Data Bank [33]. All the sites employed in the simulation were based on information from UniProt and predictions from the DoGSiteScorer tool [34,35].

**Figure 3:**
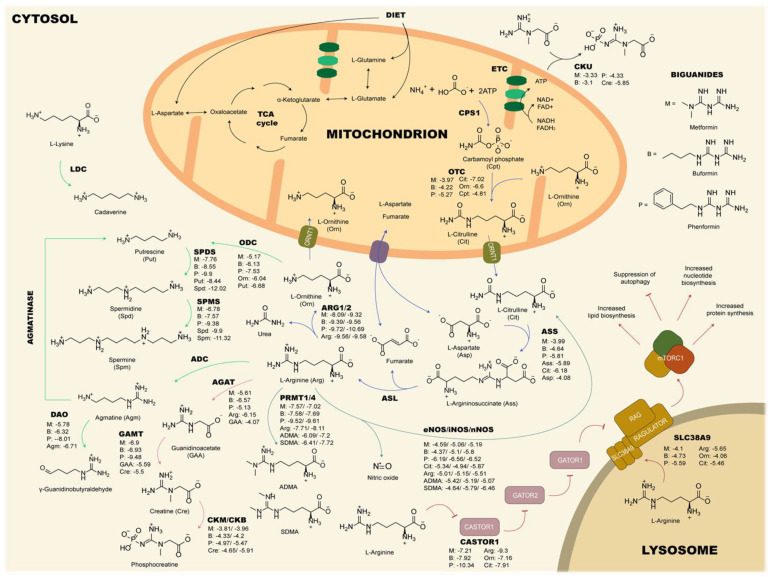
Binding energies (kcal/mol) of biguanides and selected candidate metabolites in their corresponding targets. The best affinity values of biguanides compared to those of the endogenous ligands were obtained in ARG1/2, CASTOR1, SPMS, SPDS, and GAMT, where biguanides showed the lowest binding energies, suggesting comparable or even higher affinity values than those of the corresponding ligands. These results may indicate an involvement of metformin in the urea cycle, NO metabolism, mTORC1 pathway, polyamine metabolism, and creatine biosynthesis. ADC: Arginine decarboxylase; AGAT: Arginine:glycine amidinotransferase; ARG: Arginase; ASL: Argininosuccinate lyase; ASS: Argininosuccinate synthetase; CPS: Carbamoyl phosphate synthetase; CKB: Brain-type creatine kinase; CKM: Muscle-type creatine kinase; CKU: Ubiquitous creatine kinase; DAO: Diamine oxidase; ETC: Electron transport chain; eNOS: Endothelial nitric oxide synthase; iNOS: Inducible nitric oxide synthase; nNOS: Neural nitric oxide synthase; GAMT: Guanidinoacetate N-methyltransferase; Lysine decarboxylase; ODC: Ornithine decarboxylase; SPDS: Spermidine synthase; SPMS: Spermine synthase; PRMT: Protein arginine methyltransferase; OTC: Ornithine transcarbamylase; TCA: Tricarboxylic acids.

The results from the molecular docking simulations are shown in Figure 3. In general, there was a trend for metformin, buformin and phenformin, in that order, of being the biguanides with the highest affinities to each target. Additionally, according to binding energies and predicted inhibition constants, phenformin showed even more affinity than the endogenous ligands did during their binding to some targets. For instance, the binding energies for metformin (−8.09 kcal/mol), buformin (−9.39 kcal/mol), and phenformin (−9.72 kcal/mol) in ARG1 were comparable to that obtained for L-arginine (−9.56 kcal/mol). Another example was CASTOR1, one of the L-arginine sensors in the cell, in which the binding energy for L-arginine was −9.3 kcal/mol, but in the case of metformin, buformin, and phenformin, they were −7.21 kcal/mol, −7.92 kcal/mol, and −10.34 kcal/mol, respectively. In the case of GAMT, biguanides showed comparable or even higher affinities (−6.9 kcal/mol to −9.48 kcal/mol) than guanidinoacetate did (−5.59 kcal/mol), the precursor of creatine. Furthermore, the binding energies of biguanides (from −7.76 kcal/mol to −9.9 kcal/mol) were similar to that of putrescine (−8.44 kcal/mol) in spermidine synthase. Finally, for the three isoforms of NOS, the estimated binding energies of biguanides ranged from −4.37 kcal/mol to −6.56 kcal/mol, while for L-arginine and ADMA, they ranged from −5.01 kcal/mol to −5.51 kcal/mol and from −5.07 kcal/mol to −5.42 kcal/mol, respectively. Remarkably, these results agreed with phenformin and metformin being the most and least potent biguanides, respectively [36].

### 2.6. Binding Comparison between Biguanides and L-arginine-Related Metabolites

**Figure 4:**
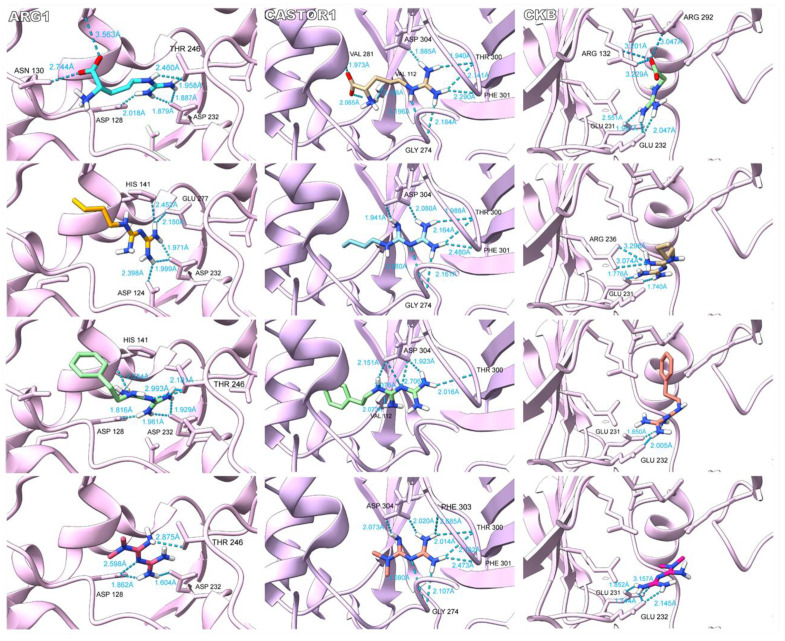
Binding modes of biguanides against the endogenous substrate of ARG1, CASTOR1 and brain-type creatine kinase (CKB). For ARG1 and CASTOR1, L-arginine, buformin, phenformin, and metformin are shown in descending order. For CKB, creatine, buformin, phenformin, and metformin are shown in descending order. As our hypothesis suggested, the guanidine pharmacophore of biguanides bound with the same orientation of the guanidine side chain of L-arginine in ARG1 and CASTOR1. Additionally, both moieties obtained hydrogen bonded to common amino acid residues. In the same way, biguanides interacted with CKB through the guanidine pharmacophore with a similar orientation to that of the guanidine group of creatine and establishing hydrogen bonds with some common amino acid residues. However, the binding of L-arginine and creatine were not completely identical to biguanides because of differences in size and the presence of the carboxylate moiety.

Despite the similarity between the predicted binding energies of biguanides and the candidate metabolites, we proceeded to assess the binding modes inside the tested targets to sustain our hypothesis about a structure-dependent cross mechanism of action. The different binding mode analyses were carried out in Chimera X 1.2.5. It is noteworthy that some biguanides’ binding modes were similar to those of candidate metabolites in their corresponding targets as shown in Figure 4 for ARG1, CASTOR1, and brain-type CK. Other interactions can be found in the Supplementary Material. We observed the establishment of hydrogen bonds between L-arginine and Asp 128, Asn 130, Ser 137, Thr 246, and Asp 232 in ARG1. Our simulation for the L-arginine binding mode in ARG1 agreed with the binding site reported in UniProt, which includes His 126, Thr 127, Asp 128, Ile 129, Asn 130, Ser 137, Gly 138, Asn 139, Asp 183, Thr 246, and Glu 277, indicating the good reproducibility of our methodology [34]. Although with some differences, biguanides established hydrogen bonds with amino acid residues present in the L-arginine binding site, including Asp 128, Thr 246, and Glu 277, among others. As well as for ARG1, in CASTOR1, our simulation for L-arginine showed good reproducibility compared to that of the reported interacting amino acid residues from UniProt [34]. In our methodology, L-arginine established hydrogen bonds with Val 112, Gly 274, Val 281, Thr 300, Phe 301, and Asp 304. With some differences, the biguanides shared interactions with different L-arginine-interacting amino acid residues, including Gly 274, Thr 300, Phe 301, and Asp 304. Finally, for the brain-type CK, the reported interacting amino acid residues in UniProt include Val 72, Glu 232, and Se 285 [34]. Our simulation for creatine predicted the binding of the guanidine moiety to Glu 231 and Glu 232 and the carboxylate moiety to Arg 132 and Arg 292. Such as creatine, the guanidine moiety of biguanides showed a tendency to establish hydrogen bonds with Glu 231 and Glu 232, except for buformin, which established two hydrogen bonds with Arg 236 instead of Glu 232. Remarkably, the orientations of the guanidine groups inside ARG1, CASTOR1, and brain-type CK for L-arginine and biguanides were strongly similar.

As can be observed, these results are in line with our previous observations, where it should be expected to observe a good concordance of binding if the molecules are supposed to be similar.

## 3. Discussion

Beyond its anti-diabetic properties, metformin has showed to exert anti-cancer effects in different types of cancer models and epidemiological studies [23]. Despite its clinical success and the progress made to elucidate its anticancer molecular actions, a complete picture of metformin’s anticancer biological effects is lacking. However, based only on structural and physicochemical characteristics, here, we proposed and sustained, with a computational methodology, our hypothesis that described another possible mechanism by which biguanides may resemble different endogenous L-arginine-related metabolites, leading to an antagonist effect in their corresponding targets.

In pharmacology, the binding of ligands to a specific target is dependent on several factors such as shape, charges, hydrogen-bonding capacity, flexibility, planarity, polarity, and size, among others [37]. According to our hypothesis, if biguanides are supposed to bind targets from arginine-related metabolites, they must be comparable at both the structural and physicochemical levels. For this reason, the methodology followed here began with the creation of a database that contains different metabolites related to L-arginine metabolism and other related metabolic pathways including the urea cycle, nitric oxide, polyamines, and creatine metabolism. Subsequently, comparisons of the structural and physicochemical elements by employing cheminformatic tools were performed. In the final step to test our hypothesis, bioinformatic tools were carried out to prove if the comparable structural and physicochemical elements between biguanides and arginine-related metabolites could be translated into a comparable binding in their corresponding targets. If this was true, it is reasonable to think that biguanides may exert an anticancer effect by affecting the metabolic pathways where these metabolites are involved. In the following paragraphs, evidence that sustains or refutes our hypothesis is provided. However, the evidence is focused on metformin mainly because it is the most widely studied biguanide.

Anabolism supports proliferation, survival, and invasion processes in cancer cells. mTORC1 is one of the most important master regulators of anabolic metabolism in normal and cancer cells [38]. The upregulation of mTORC1 has been reported to be dependent on two interrelated stimuli: proliferative signaling pathways by growth factors and nutrient sensing pathways by L-arginine, L-glutamine, and branched-chain amino acids [4]. In cancer cells, anabolism dependency on L-arginine via mTORC1 has been associated with the activation of the RAGULATOR-RAG complex in the lysosomal membrane by both SLC38A9 and CASTOR1, the two intracellular sensors of L-arginine. SLC38A9 is a transmembrane transporter of L-leucine and L-arginine located on the lysosome that activates the RAGULATOR-RAG complex in response to changes in amino acids concentrations [39]. The sensing of an amino acid via SLC38A9 has been reported to be needed in pancreatic cancer cells to form tumors [40]. On the other hand, the binding of L-arginine to CASTOR1 disrupts its suppressing interaction with GATOR2, leading to the GATOR2-dependent inhibition of GATOR1 and the activation of the RAGULATOR-RAG complex [41]. The CASTOR1-dependent inhibition of mTORC1 has shown a tumor suppressor role in lung adenocarcinoma leading to lower proliferation, migration, and invasion, and when it is downregulated, it is associated with a poor prognosis [42]. It has been reported that the inhibition of mTORC1 through competitive binding in CASTOR1 by analogs of L-arginine, including L-citrulline and L-ornithine, among others, avoids the disruption of CASTOR1–GATOR2 interaction [43]. Our hypothesis predicted that it may be possible the suppression of mTORC1 by the competitive binding of biguanides against L-arginine in CASTOR1 and SLC38A9, leading to decreased activity of the RAGULATOR-RAG complex and the consequent modulation of mTORC1. According to our results, metformin and buformin showed moderate structural similarities with L-arginine and L-ornithine, two reported ligands of CASTOR1. In addition, metformin and buformin are similar to L-ornithine in terms of the physicochemical properties. Additionally, the affinities and binding modes of all the biguanides were comparable or even higher than those obtained for L-arginine in CASTOR1 and SLC38A9. In agreement with our hypothesis and results, the inhibition of mTORC1 by a metformin treatment has been reported, independently of AMPK and TSC1/2, and in a RAG GTPase-dependent manner [44]. Recently, it has been reported that L-arginine exerts an epigenetic regulation over TEA-like domain 4 (TEAD4) in prostate cancer cells [45]. TEAD4 is a transcription factor that controls the expression of genes involved in oxidative phosphorylation. This suggests that global metabolism and epigenetics can be controlled by L-arginine in cancer cells via different sensing systems, such as CASTOR1, SLC38A9, and possibly TEAD4. As our hypothesis predicted, there was reported the metformin targeting of the YAP1–TEAD4 axis in bladder cancer cells [46].

The urea cycle is related to several metabolic pathways that allow anabolism. For instance, the urea cycle was reported to be linked to the TCA via fumarate and oxalacetate-derived L-aspartate [47]. Given this connection, the urea cycle disruptions were reported to modify TCA metabolites, leading to the metabolic and epigenetic changes needed for cancer cells. It is noteworthy that some essential building blocks and epimetabolites are directly derived from TCA, such as α-ketoglutarate [48]. For instance, it has been reported that the fumarate accumulation in cancer cells allowed epithelial-to-mesenchymal transition due to the inhibition of the α-ketoglutarate-dependent dioxygenases, proteins with histone demethylase activity [49]. Our results may indicate a possible reduction of urea cycle metabolites after the metformin treatment due to a cross structure-dependent inhibition of key enzymes. According to our analyses, metformin and buformin showed moderate-to-high similarities when they were compared at both the structural and physicochemical levels with some urea cycle metabolites, including L-aspartic acid, L-arginine, and L-ornithine. As expected, the structural and physicochemical similarities were translated in comparable affinities and binding modes of biguanides with urea cycle enzymes, at least in ARG1 and ARG2. Similar to this study, Detroja and Samson (2022) reported the possible inhibition of ARG1 by metformin based on molecular docking simulations and molecular dynamics. They found that the binding of metformin to the active sites of both ARG1 and ARG2 was stable for up to 50 ns in molecular dynamics simulations [50]. There is evidence of biguanides affecting the urea cycle. According to Zhang et al. (2021), metformin has been shown to negatively regulate the urea cycle intermediates including L-arginine and L-aspartate in an in vivo xenograft model of HCT116, a colorectal cancer cell line [51]. However, the authors reported that this reduction was due to the decreased expression in urea cycle enzymes such as CPS1, ARG1, and OTC. Additionally, it has been reported that the metformin treatment decreased the ARG1 activity of granulocytic myeloid-derived suppressor cells in a tumor-bearing mouse model of colon carcinoma [52]. Furthermore, the decreased arginase activity was maintained despite the use of compound C, an AMPK inhibitor, suggesting that the reduction of arginase activity was independent on AMPK. In the same way, Koroglu-Aydin et al. (2021) reported a decreased activity of arginase in kidney homogenates from rats with diabetes and prostate cancer treated with metformin compared to that of non-treated rats [53].

When they are altered, polyamines in cancer have been associated with increased proliferation, survival, and resistance to therapy, i.e., a poor cancer prognostic. In the case of polyamine metabolism, our results strongly supported the resemblance between biguanides and some specific intermediates. There was a remarkable structural similarity between buformin and metformin with agmatine and L-ornithine. These relationships were maintained during the physicochemical comparisons, although not in both modalities. As well as for the UC, metformin was capable of decreasing the concentrations of putrescine via decreased expression of ODC in an in vivo xenograft model of HCT116 [51].

Cell division is a high-energy-demanding process that needs to be supported by plenty of energy. In cancer cells, a reported strategy to maintain the cell cycle forward was the overexpression of creatine kinases isoforms, especially in those sites where a lot of energy is needed [54]. For these reasons, the phosphagen system represents one of the most important ways of storing, buffering, and transferring energy in tumors. Regarding creatine metabolism, our data indicated a competitive binding of biguanides against targets related to creatine biosynthesis or usage and the consequent disruption of cell bioenergetics. Our results showed moderate-to-high similarity at both the 2D and, especially, 3D levels. Additionally, the hierarchical clustering of biguanides with guanidinoacetic acid and creatine suggested a close relationship in terms of the physicochemical properties. On the other hand, the affinities and, for some isoforms of creatine kinases, the binding modes of biguanides were comparable to those obtained for creatine. It is noteworthy that metabolites belonging to creatine metabolism were the ones most related to biguanides, especially metformin and buformin, according to our results during structural, physicochemical, and binding comparisons. Recently, has authors have reported the competitive inhibition of AGAT by a metformin treatment (3 × 1500 mg daily for 6 weeks) in individuals with Becker muscular dystrophy, leading to reduced concentrations of guanidinoacetic acid in both serum and urine [55]. Remarkably, decreased renal and pancreatic AGAT activity, but not decreased mRNA expression, have been reported after creatine supplementation in rats, suggesting that creatine can be involved in a negative feedback regulation mechanism [56]. If biguanides were comparable to creatine, it may be possible that biguanides could become involved in that regulation, resembling creatine. Regarding the evidence supporting the action of biguanides on phosphocreatine biosynthesis, Garbati et al. (2017) reported the decrement of 40% in brain-type creatine kinase activity by metformin treatment (10 mM) during enzymatic assays, and its lowering effect on the ATP/AMP ratio of the KM-H2, SHSY-5Y, and MDA-MB-468 cancer cell lines [57]. It is noteworthy that metformin was suggested to bind to a site different from that for creatine binding, acting in a non-competitive manner. It is possible that metformin may bind to the other isoforms of creatine kinases given the strong homology of this family of proteins [58].

It is noteworthy that our main limitation was that our results were generated through a computational methodology. Hence, the experimental demonstration is needed to sustain or refute the results presented here and to test our hypothesis. Another limitation of our computational methodology was the absence of molecular dynamics simulations to test the binding of biguanides and L-arginine-related metabolites to the targets of interest in a more reliable manner, since both the protein and the ligand are flexible [59]. However, the use of molecular dynamics simulations was beyond the scope of our original methodology, as our principal aim was to establish our hypothesis and to provide preliminary computational evidence about the possible structural, physicochemical, and binding similarities that biguanides share with some L-arginine-related metabolites, as a first approach. In the future, molecular dynamics simulations are intended to be included with an experimental methodology to provide stronger evidence for or against our hypothesis. Additionally, as mentioned above, authors have already reported the molecular dynamics simulations of metformin’s binding to ARG1/2, partially strengthening our molecular docking simulations results for metformin in the same targets [50]. Another opportunity for improvement in the future is that in the present study, we limited our computational analysis to certain L-arginine-related metabolites, but there are many metabolites that may also be related structurally and functionally to biguanides, such as N(G)-monomethyl-L-arginine, L-proline, L-glutamate, or L-homoarginine, as well as different biguanides, including galegine or even microbiome-derived metabolites of biguanides.

## 4. Materials and Methods

### 4.1. Database Creation

The database was created including different candidate metabolites related to L-arginine metabolism in the cell, including compounds from the urea cycle, creatine biosynthesis, nitric oxide metabolism, and polyamines metabolism, among others. Given that buformin and phenformin are more potent drugs than metformin is, but belong to the same pharmacological class, we decided to include both drugs to enrich the analyses. For every molecule, its CID number and SMILES from PubChem were collected.

### 4.2. Structural Comparison between Biguanides and L-arginine-Related Metabolites

Assessment of the 2D and 3D similarities between biguanides and candidate metabolites were carried out employing the PubChem Score Matrix Service [60]. In the case of the 3D similarity assay, both shape optimized and feature optimized analyses were carried out for 10 conformers per CID. Two-dimensional similarity assays generated a TC value that ranged from 0 to 100 depending on the grade of similarity, with 0 being a null similarity and 100 being an identical molecule. Three-dimensional similarity assays generated two scores: ST assessed the shape and CT assessed the features (e.g., hydrogen bond donors and acceptors, rings, etc.) of the compared molecules. Both scores ranged from 0 to 100 following the same logic as that used for TC. When these two scores were summed up, combo T was generated [61]. This score ranged from 0 to 200 and was used to assess shape and features together in only one parameter. Given that the TC cut offs ranged from 50 to 80 in the literature, for 2D similarity, the scores were “high” when TC ≥ 65. In the case of 3D similarity, it was “high” when ST ≥ 80 and CT ≥ 50, according to Bolton et al. (2011), or when combo T ≥ 130 [61]. For every pair of compared molecules, their TCs were collected from the 2D analysis, as well as their ST and CT from the 3D analysis. Additionally, the identification of a common scaffold between biguanides and the structurally related metabolites was performed in the SwissSimilarity tool (http://www.swisssimilarity.ch/ (accessed on 10 January 2023)). This tool is capable of performing high-throughput screening of molecules similar to an input molecule based on 2D and 3D molecular descriptors. The search can be performed in different classes of compounds, e.g., drugs and commercial or bioactive substances. For this methodology, we introduced the different SMILES in the menu and selected “Bioactive” as the class of compounds. Finally, we chose the Chemical Entities of Biological Interest (ChEBI) as the library and “Scaffold” as the screening method [62].

### 4.3. Physicochemical Comparison between Biguanides and L-arginine-Related Metabolites

The physicochemical properties of biguanides and candidate metabolites presented in the database were predicted by using the collected SMILES of every molecule with the SwissADME tool (http://www.swissadme.ch/ (accessed on 21 December 2022)) [31]. The physicochemical comparisons were performed in both ionized and non-ionized modalities. For the ionized modality, all the molecules in our database were ionized to physiological pH of 7.4 according to scientific reports and their corresponding new SMILES were collected. For every molecule, several physicochemical parameters were collected, including molecular weight, accounting for size, hydrogen bond donors and hydrogen bond acceptors, accounting for hydrogen-bonding capacity, consensus log P and TPSA, accounting for polarity, rotatable bonds, accounting for flexibility, and sp^3^-carbon fraction, accounting for planarity. After this, a PCA of the collected variables was performed as an exploratory analysis of data from physicochemical profiles. Finally, an HC was carried out aiming to test if an unsupervised machine learning algorithm was capable of identifying a relationship between biguanides and candidate metabolites. Each biguanide was compared, one at a time, against the candidate metabolites. PCA and HC were performed in the R software (version 4.2.2) by employing RStudio as our graphical user interface and some R-packages including tidyverse, factoextra, cluster, ggplot2, ggcorrplot, and readr [63,64,65,66,67,68,69]. Data analysis began with the loading of our database in RStudio. This database contained biguanides and our candidate metabolites and all their collected physicochemical parameters. However, these data are presented at different scales. For this reason, these magnitudes were scaled into the standard scale of the Z-score. PCA was performed by using the prcomp function based on singular value decomposition. Indeed, according to R documentation, a better numerical accuracy was reported for this method compared with that of the eigen decomposition. Additionally, it is not necessary to generate the covariance matrix in this method. On the other hand, the clusters of the HC were generated by calculating the Euclidean distances and employing the Ward’s linkage with the hclust function. All dendrograms were generated in RStudio using the packages mentioned above.

### 4.4. Structure-Based Target Prediction of Biguanides and L-arginine-Related Metabolites

The biguanides were subjected to a structure-based target prediction by using the collected SMILES employing the SwissTargetPrediction Tool (www.swisstargetprediction.ch/ (accessed on 29 December 2022)) from the Swiss Institute of Bioinformatics [32]. In a similar manner to our hypothesis, the SwissTargetPrediction tool is based on the chemical similarity, i.e., the algorithm was trained with a big collection of molecules (almost 400,000), whose targets were identified experimentally. In simple terms, when a new ligand is presented to the algorithm, it searches for similar molecules at the 2D and the 3D levels, returning their associated macromolecules as possible targets for the new ligand. The output of a SwissTargetPrediction assay are two parameters: the probability score and the known actives (3D/2D). The probability score represents a combined score of the 2D and 3D similarity values between the input ligand and those molecules from the algorithm. The known actives (3D/2D) represent the list of molecules similar at the 2D and the 3D levels to those of the input ligand, whose interaction has been demonstrated experimentally. In this methodology, for every molecule, its predicted biological targets associated with candidate metabolites and their corresponding probability scores and known actives (3D/2D) were collected. After this, we confirmed if the interactions between the predicted targets and the tested ligands have been reported in scientific literature.

### 4.5. Affinity Comparison between Biguanides and L-arginine-Related Metabolites

Once we predicted the targets for biguanides and candidate metabolites, a molecular docking simulation was carried out employing AutoDock 4.2 [70]. Other non-predicted targets whose ligand or substrate was reported to be one of the candidate metabolites included in our database were subjected to simulation. All the protein structures for the simulation were obtained from Protein Data Bank [33]. Our search was limited to PDB files with a resolution lower than 3Å and derived from Homo sapiens. In the case of SLC38A9, we used a PDB file from Danio rerio because the human files showed lower resolution. The water molecules and other ligands co-crystallized with the protein were removed from the PDB files. The tested candidate metabolites were constructed in Avogadro based on the different collected SMILES [71]. Additionally, hydrogen atoms were added to the ligands, simulating a pH of 7.4. Finally, the structures of ligands were optimized in Avogadro using both the force field MMFF94 and the algorithm steepest descent, and they were saved as .mol2 files.

AutoDock Tools (ADT) was employed as the graphical user interface to perform the docking simulations. In ADT, polar-only hydrogens and Kollman charges were added to proteins, while ligands were subjected to additions of polar-only hydrogens and Gasteiger charges. The docking sites were chosen based on information from the UniProt database and based on the predictions obtained from the DoGSiteScorer tool [34,35]. The complete 3D coordinates used for docking simulations are shown in the Supplementary Material. During the simulations, the ligand was flexible, but the protein was maintained in a rigid modality. The affinity maps of simulations were computed using a grid spacing of 0.375Å. Additionally, we used a Lamarckian genetic algorithm for simulations employing a population of 150, with a rate of mutation of 0.2 and a maximum number of generations of 27,000.

For every simulation, its predicted binding energy and inhibition constant were collected. Our molecular docking methodology was validated through redocking. The validation methodology used here is shown in the Supplementary Material.

### 4.6. Binding Comparison between Biguanides and L-arginine-Related Metabolites

In order to analyze the different binding modes obtained from molecular docking simulations, Chimera X 1.2.5 was used to study the interactions, orientation, and conformations of biguanides and candidate metabolites in the tested targets [72]. For this analysis, we included the conformations that showed the most negative binding energies from the previous step. Additionally, we calculated the distances and found the established hydrogen bonds between the different ligands and the tested targets using the H-bonds tool from the Structure Analysis menu. According to the documentation of Chimera X 1.2.5, the H-bonds tool identifies the possible hydrogen bonds based on the atom types present in the macromolecule and the ligand and geometric criteria. The settings were established as follows: the radius at 0.075 Å, the tolerance distance at 0.4 Å, and the angle tolerance at 20°. Established interactions and conformations were compared with data from UniProt and scientific reports. All the figures shown here were generated using Chimera X 1.2.5.

## 5. Conclusions

Nowadays, therapies targeting metabolic reprogramming are gaining relevance due to the growing field of metabolomics applied to cancer research and the new findings about metabolic vulnerabilities in some specific cancer types. Metformin has been reported to alter L-arginine metabolism and other related metabolic pathways in different biological models, including humans, such as ADT, suggesting a potential use for cancer treatment in combination with chemotherapy. However, the molecular actions by which metformin performed such biological effects are incomplete. Here, we demonstrated a possible relationship between biguanides, especially metformin and buformin, with some L-arginine-related metabolites, particularly those from creatine metabolism, using a computational methodology based on cheminformatic and bioinformatic tools. The results obtained here, and the evidence discussed may suggest a new possible mechanism of action in which biguanides may resemble L-arginine and its related metabolites, leading to the modulation of their corresponding targets. In the future, this structure-dependent cross mechanism of action must be confirmed with experimental evidence. Additionally, the elucidation of the complete metformin’s mechanism of action can contribute to establishing better therapy interventions through the rational design of chemotherapy combinations and the repurposing of metformin for other diseases.

## Figures and Tables

**Table 1:** Structural comparisons of biguanides and candidate metabolites at 2D and 3D levels.

	Metformin				Buformin				Phenformin			
	TC	ST	CT	Comb	TC	ST	CT	Comb	TC	ST	CT	Comb
L-Aspartic acid	16	**89**	41	**130**	26	**80**	11	91	14	59	7	66
Fumaric acid	7	**90**	0	90	14	79	0	79	14	65	0	65
L-Argininosuccinic acid	29	49	10	59	34	60	9	69	24	71	10	81
Agmatine	56	**82**	40	122	**88**	**86**	34	120	32	62	27	89
L-Arginine	31	**82**	21	103	46	**81**	21	121	27	77	17	91
Buformin	63	75	45	120	**100**	**100**	**100**	**200**	33	69	40	111
Cadaverine	32	**88**	13	101	58	79	15	94	26	63	11	74
Carbamoyl phosphate	12	**84**	10	94	12	69	9	78	5	56	5	61
L-Citrulline	23	74	13	87	31	**84**	8	92	20	**84**	7	91
Creatine	46	**93**	36	129	42	77	25	102	20	69	14	83
ADMA	38	75	16	91	43	**81**	15	96	26	**87**	7	94
SDMA	33	73	18	91	39	73	15	88	25	**82**	9	91
Spermidine	35	**80**	13	93	54	**85**	25	110	22	66	25	91
Spermine	36	58	11	69	55	70	9	79	25	75	11	86
Phenformin	23	68	21	89	33	69	40	109	**100**	**100**	**100**	**200**
Guanidinoacetic acid	36	**95**	35	130	50	75	29	104	22	61	21	82
L-Ornithine	19	**92**	17	109	32	**81**	11	92	19	66	10	76
Metformin	**100**	**100**	**100**	**200**	63	75	47	122	23	68	20	89
Putrescine	34	**82**	17	99	62	62	31	93	23	53	13	66
Urea	25	61	14	75	24	46	12	58	9	31	11	42

High scores of TC, ST, or CT are highlighted in bold.

## Data Availability

Data are contained within the article or Supplementary Materials.

## Supplementary Materials

The following supporting information can be downloaded at: https://www.mdpi.com/article/10.3390/ijms24065316/s1.
