# Supplementary material for: Metformin May Alter the Metabolic Reprogramming in Cancer Cells by Disrupting the L-Arginine Metabolism: A Preliminary Computational Study
Source: Int J Mol Sci. 2023 Mar 10;24(6):5316. doi: 10.3390/ijms24065316 (PMC10049129; doi:10.3390/ijms24065316)
Supplement: Supplementary file 1 [file ijms-24-05316-s001.zip › Supplementary material (Part A) IJMS - Isaias Balderas.pdf]

Metformin may alter the metabolic reprogramming in cancer cells by disrupting the L-arginine metabolism: A preliminary computational study

Bryan Alejandro Espinosa-Rodriguez [1], Daniela Treviño-Almaguer [1], Pilar Carranza-Rosales [2], Monica Azucena Ramirez-Cabrera [1], Karla Ramirez-Estrada [1], Eder Ubaldo Arredondo-Espinoza [1], Luis Fernando Mendez-Lopez [3],*, and Isaias Balderas-Renteria [1],*

Authors affiliation:

[1] Universidad Autonoma de Nuevo Leon, School of Chemistry, Laboratory of Molecular Pharmacology and Biological Models, San Nicolas de los Garza, Nuevo Leon, 66451, Mexico

[2] Centro de Investigacion Biomedica del Noreste, Laboratory of Cell Biology, Instituto Mexicano del Seguro Social, Monterrey, Nuevo Leon, 66720, Mexico

[3] Universidad Autonoma de Nuevo Leon, School of Public Health and Nutrition, Center for Research on Nutrition and Public Health, Monterrey, Nuevo Leon, 66460, Mexico

*Correspondence: I.B.R.: isaias.balderasrn@uanl.edu.mx, ibalderas@hotmail.com; Tel.: +52 81-8329-4000. L.F.M.L.: luis.mendezlop@uanl.edu.mx; Tel: +52 81-1042-2622.

Complementary results of the structural comparison at the 2D and 3D levels

The complete results of both 2D and 3D structural comparisons between biguanides and the candidate metabolites can be observed in Supplementary Material part B, including the different TC, ST, CT, and combo T values.

Complementary results of the physicochemical comparison

The complete list of physicochemical parameters employed in the physicochemical comparisons between biguanides and the candidate metabolites can be observed in Supplementary Material part B, including the molecular weight, consensus log P, topological polar surface area, hydrogen bond donors, hydrogen bond acceptors, Csp^3 fraction, and rotatable bonds.

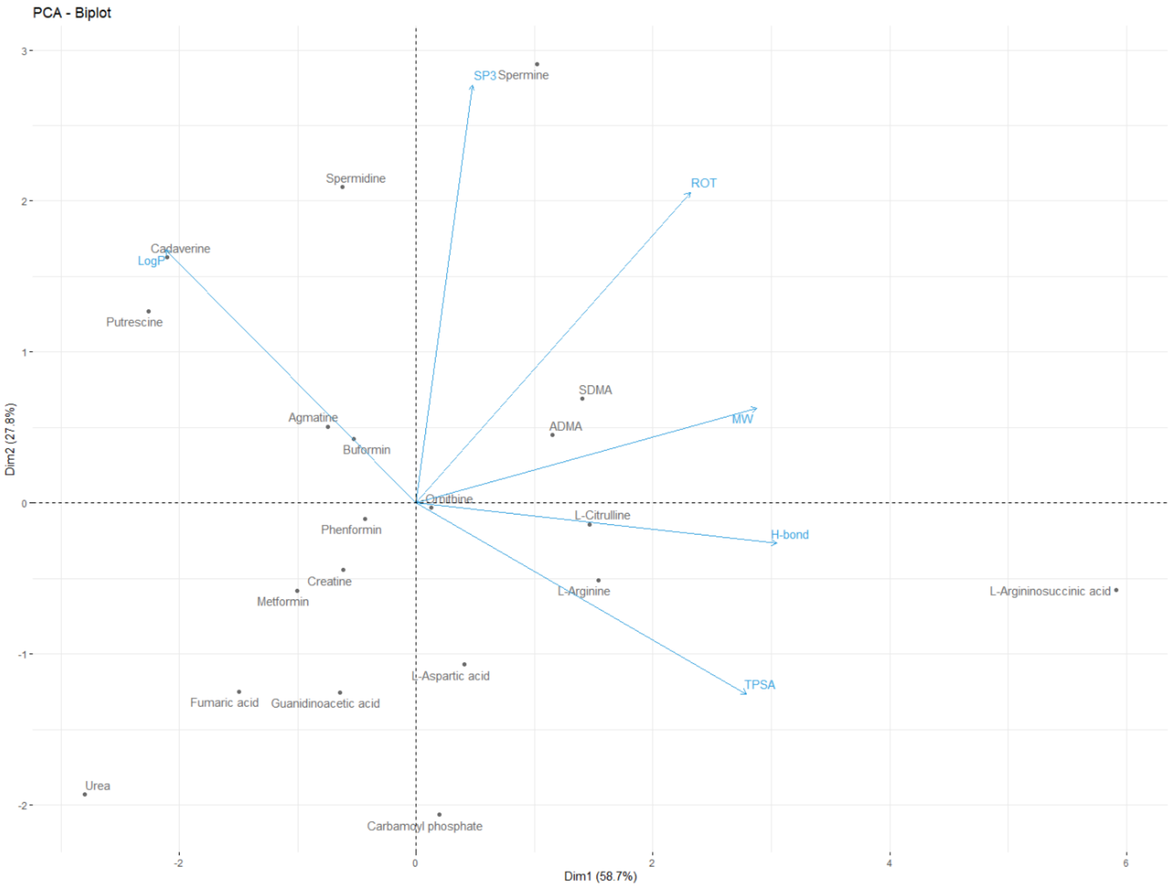

Figure S1. Principal component analysis of biguanides and candidate metabolites in the non-ionized modality

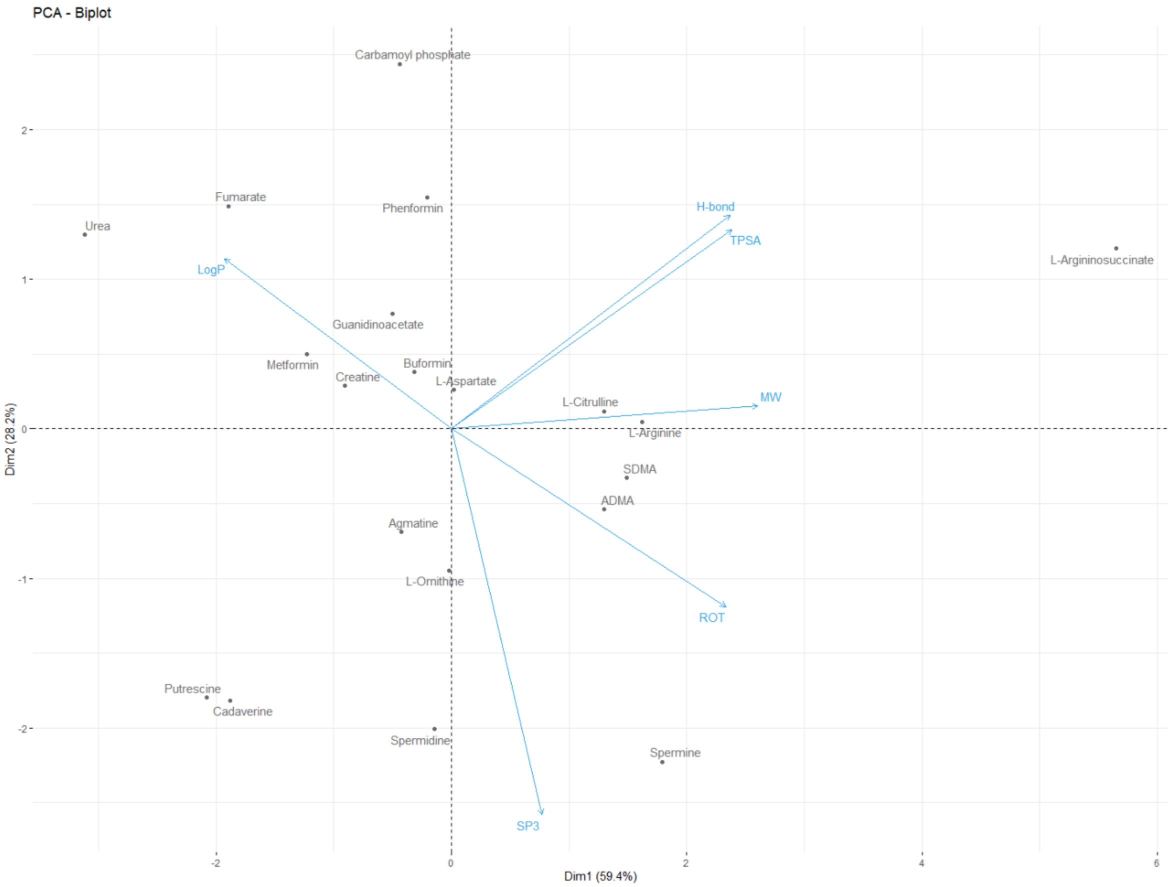

Figure S2. Principal component analysis of biguanides and candidate metabolites in the ionized modality

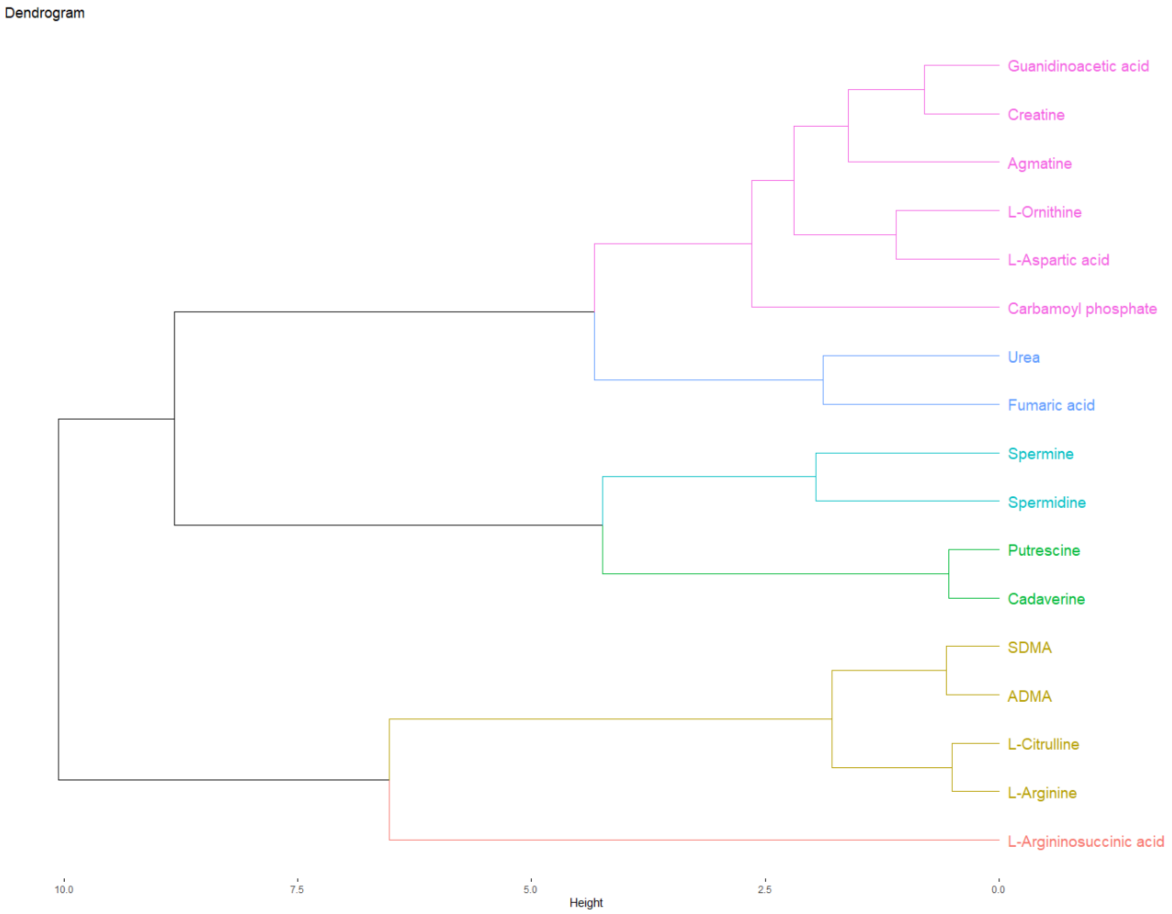

Figure S3. Hierarchical clustering of candidate metabolites in the non-ionized modality

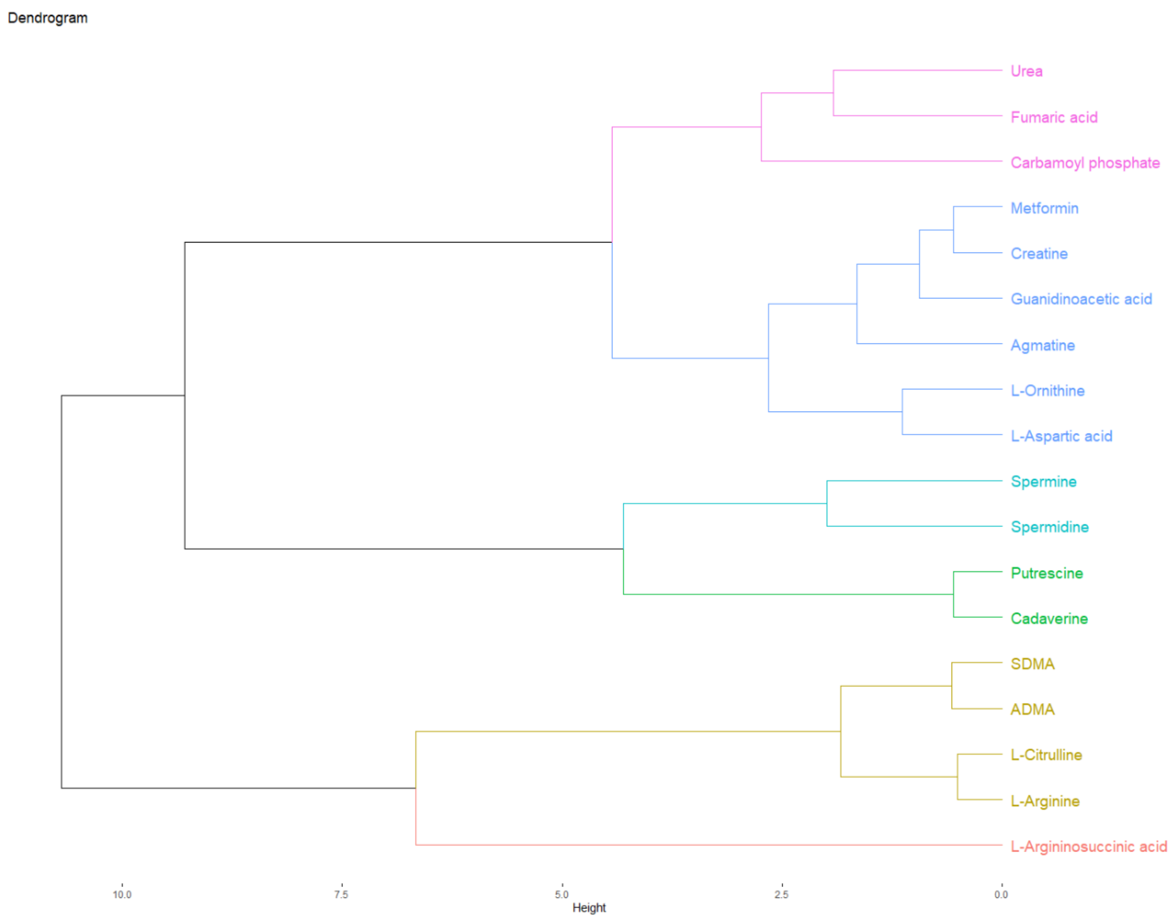

Figure S4. Hierarchical clustering of metformin and candidate metabolites in the non-ionized modality

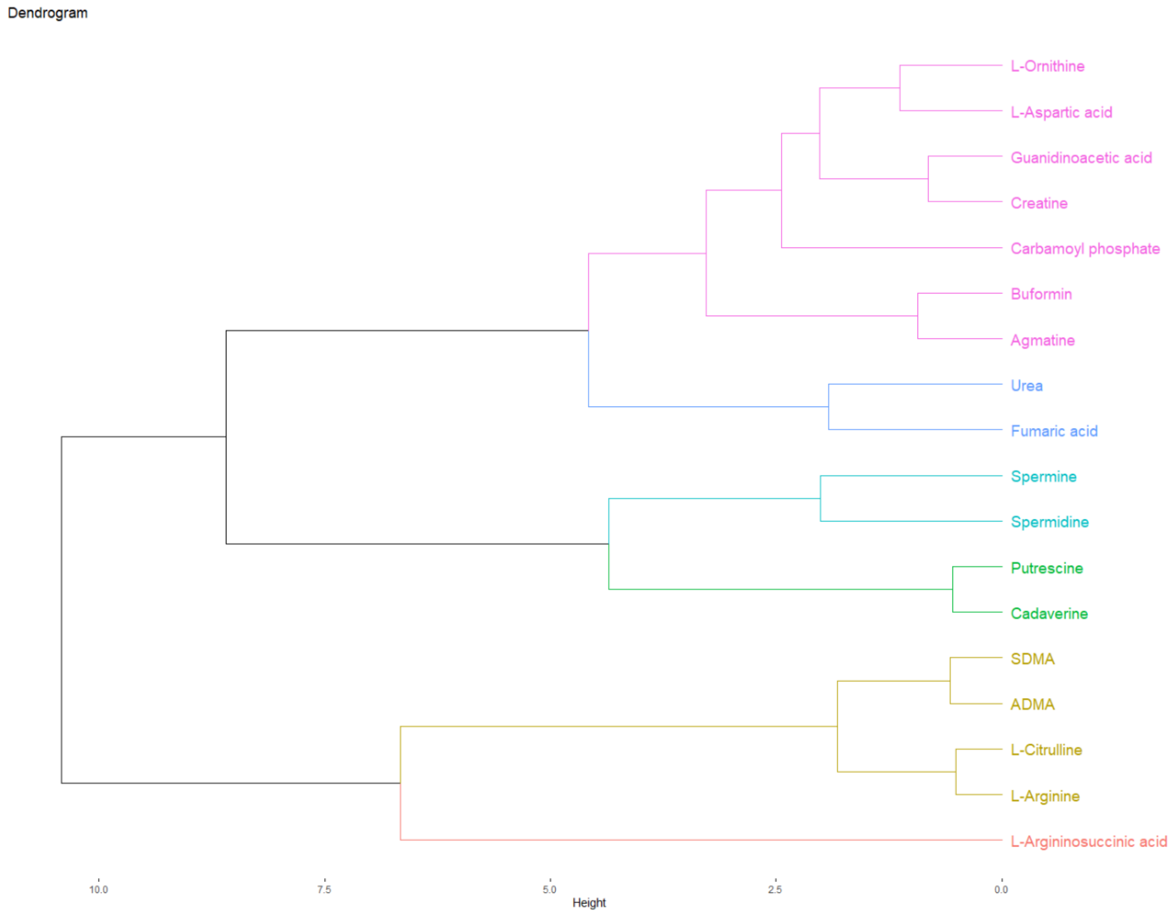

Figure S5. Hierarchical clustering of buformin and candidate metabolites in the non-ionized modality.

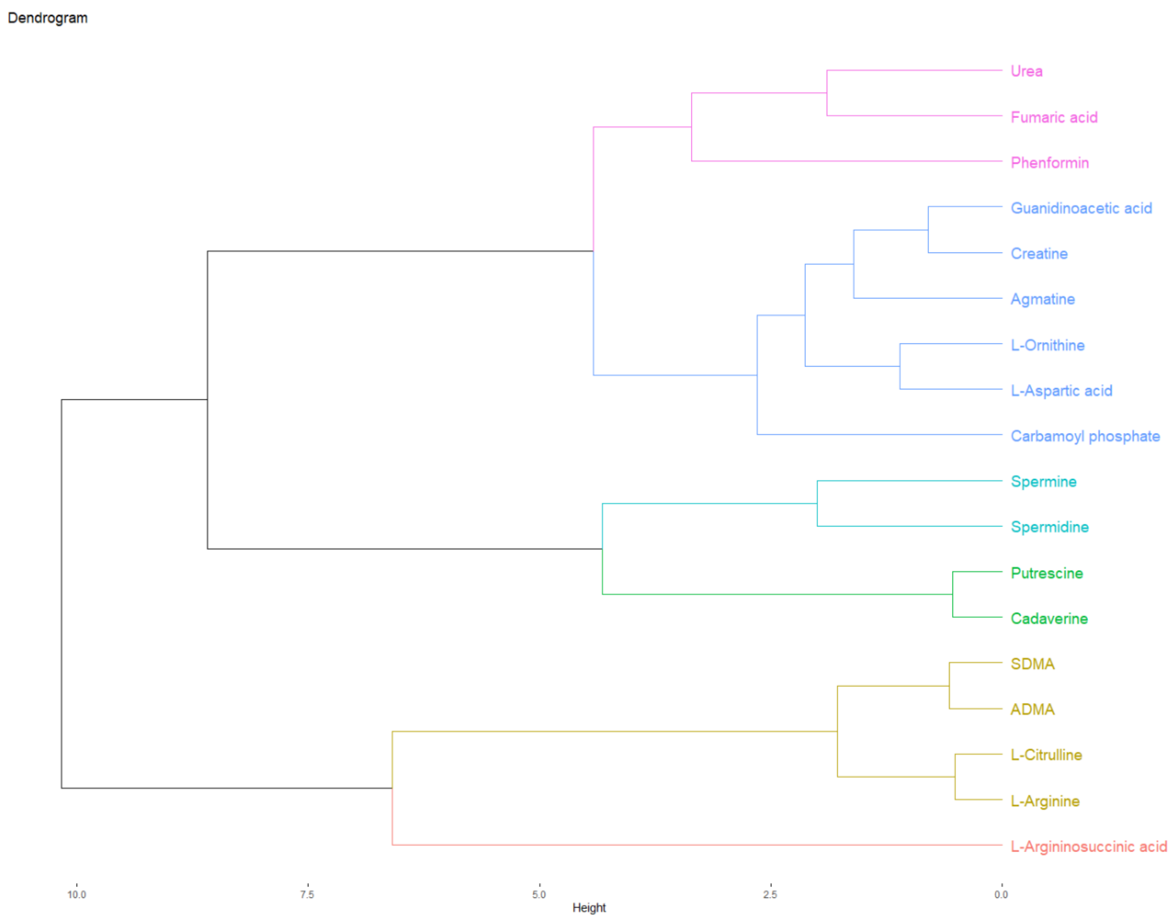

Figure S6. Hierarchical clustering of phenformin and candidate metabolites in the non-ionized modality.

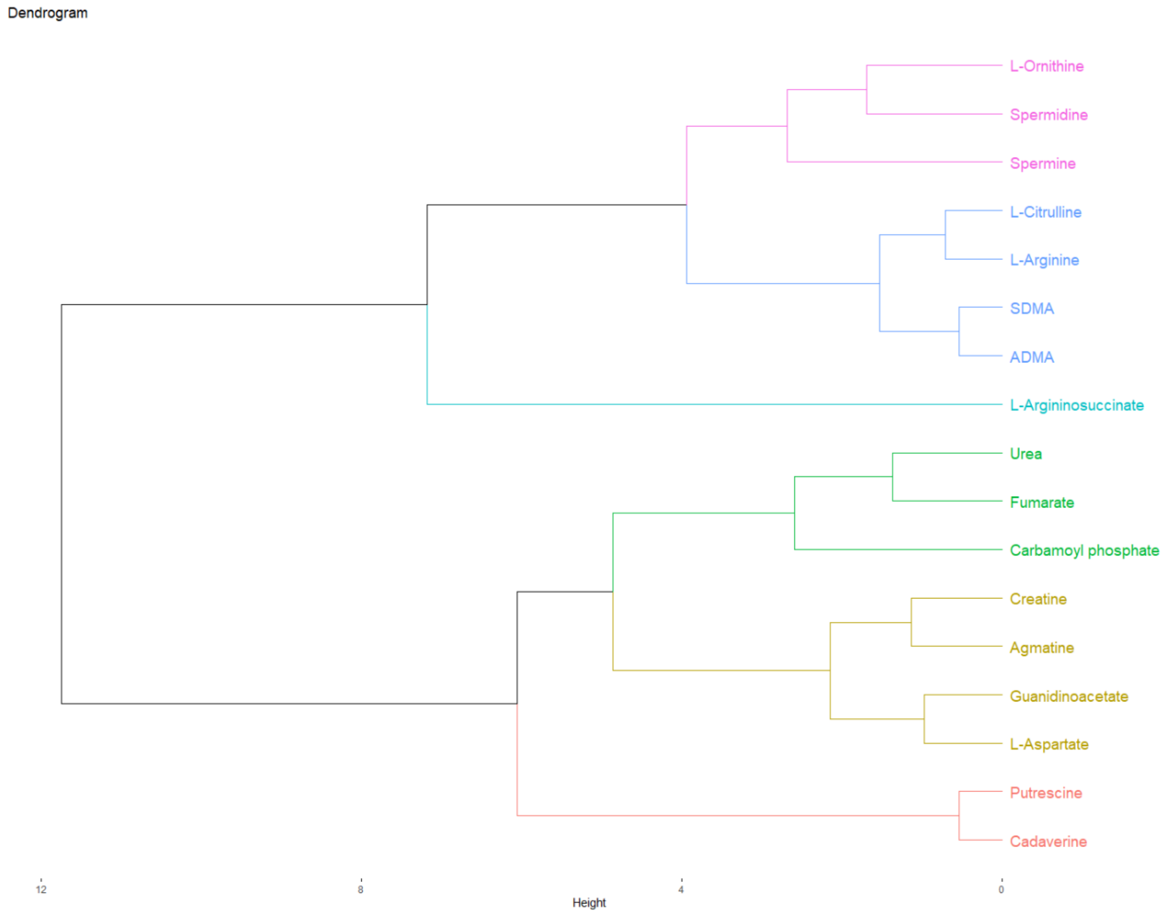

Figure S7. Hierarchical clustering of candidate metabolites in the ionized modality.

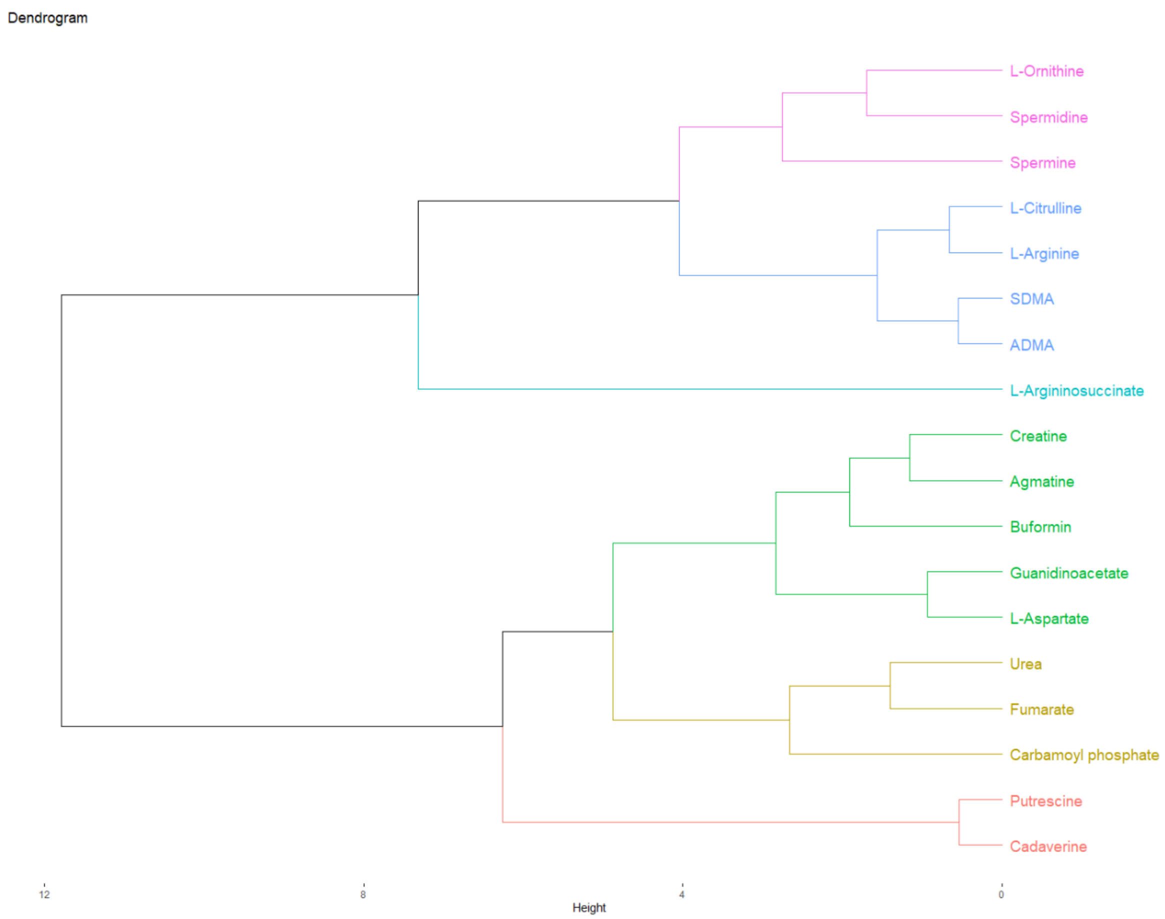

Figure S8. Hierarchical clustering of buformin and candidate metabolites in the ionized modality.

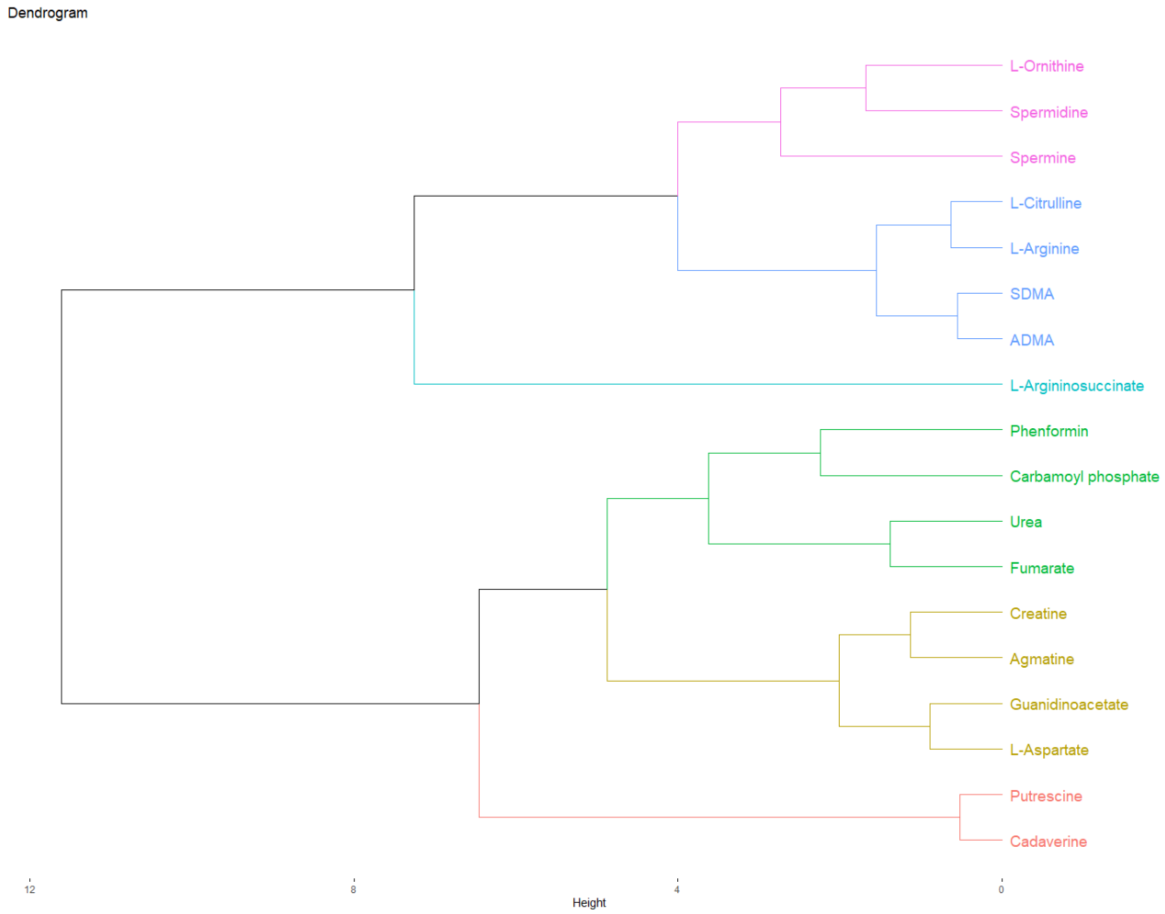

Figure S9. Hierarchical clustering of phenformin and candidate metabolites in the ionized modality.

Methodology and complementary results of molecular docking simulations

- **Search of PDB files**

After we predicted the possible targets for biguanides using the SwissTargetPrediction tool, we decided to add targets whose ligand or substrate is reported to be one included in our arginine-related metabolites database. Our final list of candidate targets was composed of four urea cycle enzymes including arginase 1 (ARG1), arginase 2 (ARG2), ornithine transcarbamylase (OTC), and argininosuccinate synthase (ASS); three enzymes involved in nitric oxide production including inducible nitric oxide synthase (iNOS), neural nitric oxide synthase (nNOS), and endothelial nitric oxide synthase (eNOS); three enzymes involved in the production and breakdown of asymmetric dimethylarginine including protein arginine methyltransferase (PRMT) 1, 4, and dimethylarginine dimethylaminohydrolase 1 (DDAH1); five enzymes from creatine metabolism, including the muscle-type, brain-type, and ubiquitous creatine kinases (CK), arginine:glycine amidinotransferase (AGAT), and guanidinoacetate N-methyltransferase (GAMT); four enzymes from polyamine metabolism spermidine synthase (SPDS), spermine synthase (SPMS), ornithine decarboxylase, and diamine oxidase (DAO); two intracellular sensors of L-arginine, including CASTOR1 and SLC38A9. Once we defined the candidate targets, we search for them in the Protein Data Bank. We limited our search to *Homo sapiens* and proteins with a resolution lower than 3 Å. In the case of SLC38A9, we used a PDB file from *Danio rerio* because the human PDB files had a lower quality in terms of resolution. The targets that we selected and their corresponding PDB codes are shown below in Table S1.

Table S1. Selected targets for molecular docking and their corresponding PDB codes

Target	PDB code
ARG1	6Q92
ARG2	6Q37
OTC	1C9Y
ASS	2NZ2
iNOS	3E7G
eNOS	4D1O
nNOS	4D1N
PRMT1	6NT2
PRMT4	5DWQ
DDAH1	3I2E
ODC	7S3G
CKB	3B6R
CKU	1QK1
CKM	1I0E
GAMT	3ORH
AGAT	2JDW
CASTOR1	5I2C
SLC38A9	6C08
DAO	3HIG
SPMS	3C6K
SPDS	2O06

- **Validation of protein targets**

Once we identified the targets for molecular docking, we proceeded to download them in.pdb format and began with the quality analysis. First, we uploaded the different pdb files into the MolProbity platform to assess the quality of the crystallized protein models. Below, we show screen captures of the quality reports obtained in MolProbity.

Summary statistics

All-Atom Contacts	Clashscore, all atoms:	1.74		99th percentile* (N=598, 1.50Å ± 0.25Å)
	Clashscore is the number of serious steric overlaps (> 0.4 Å) per 1000 atoms.			
Protein Geometry	Poor rotamers	2	0.38%	Goal: <0.3%
	Favored rotamers	507	95.30%	Goal: >98%
	Ramachandran outliers	2	0.32%	Goal: <0.05%
	Ramachandran favored	611	96.68%	Goal: >98%
	Rama distribution Z-score	-0.50 ± 0.32		Goal: abs(Z score) < 2
	MolProbity score^	1.14		98th percentile* (N=4836, 1.50Å ± 0.25Å)
	Cβ deviations >0.25Å	0	0.00%	Goal: 0
	Bad bonds:	0 / 4930	0.00%	Goal: 0%
	Bad angles:	0 / 6688	0.00%	Goal: <0.1%
Peptide Omegas	Cis Prolines:	0 / 42	0.00%	Expected: ≤1 per chain, or ≤5%
	Cis nonProlines:	2 / 592	0.34%	Goal: <0.05%
Additional validations	Chiral volume outliers	0/770		
	Waters with clashes	0/0	0.00%	See UnDowser table for details

In the two column results, the left column gives the raw count, right column gives the percentage.
* 100th percentile is the best among structures of comparable resolution; 0th percentile is the worst. For clashscore the comparative set of structures was selected in 2004, for MolProbity score in 2006.
^ MolProbity score combines the clashscore, rotamer, and Ramachandran evaluations into a single score, normalized to be on the same scale as X-ray resolution.
Key to table colors and cutoffs here:

Figure S10. Quality report for 6Q92 (ARG1) obtained from MolProbity.

Summary statistics

All-Atom Contacts	Clashscore, all atoms:	1.77		100th percentile* (N=773, 1.90Å ± 0.25Å)
	Clashscore is the number of serious steric overlaps (> 0.4 Å) per 1000 atoms.			
Protein Geometry	Poor rotamers	13	4.71%	Goal: <0.3%
	Favored rotamers	251	90.94%	Goal: >98%
	Ramachandran outliers	1	0.31%	Goal: <0.05%
	Ramachandran favored	310	97.18%	Goal: >98%
	Rama distribution Z-score	-1.27 ± 0.43		Goal: abs(Z score) < 2
	MolProbity score^	1.59		92nd percentile* (N=12147, 1.90Å ± 0.25Å)
	Cβ deviations >0.25Å	2	0.66%	Goal: 0
	Bad bonds:	13 / 2585	0.50%	Goal: 0%
	Bad angles:	35 / 3497	1.00%	Goal: <0.1%
Peptide Omegas	Cis Prolines:	1 / 13	7.69%	Expected: ≤1 per chain, or ≤5%
Additional validations	Chiral volume outliers	0/398		
	Waters with clashes	0/0	0.00%	See UnDowser table for details

In the two column results, the left column gives the raw count, right column gives the percentage.
* 100th percentile is the best among structures of comparable resolution; 0th percentile is the worst. For clashscore the comparative set of structures was selected in 2004, for MolProbity score in 2006.
^ MolProbity score combines the clashscore, rotamer, and Ramachandran evaluations into a single score, normalized to be on the same scale as X-ray resolution.
Key to table colors and cutoffs here:

Figure S11. Quality report for 1C9Y (OTC) obtained from MolProbity.

Summary statistics

All-Atom Contacts	Clashscore, all atoms:	6.59		98th percentile* (N=331, 2.40Å ± 0.25Å)
	Clashscore is the number of serious steric overlaps (> 0.4 Å) per 1000 atoms.			
Protein Geometry	Poor rotamers	31	9.09%	Goal: <0.3%
	Favored rotamers	276	80.94%	Goal: >98%
	Ramachandran outliers	2	0.51%	Goal: <0.05%
	Ramachandran favored	380	95.96%	Goal: >98%
	Rama distribution Z-score	-1.99 ± 0.38		Goal: abs(Z score) < 2
	MolProbity score^	2.37		77th percentile* (N=8058, 2.40Å ± 0.25Å)
	Cβ deviations >0.25Å	15	4.02%	Goal: 0
	Bad bonds:	4 / 3270	0.12%	Goal: 0%
	Bad angles:	15 / 4419	0.34%	Goal: <0.1%
Peptide Omegas	Cis Prolines:	0 / 20	0.00%	Expected: ≤1 per chain, or ≤5%
	Twisted Peptides:	4 / 399	1.00%	Goal: 0
Additional validations	Tetrahedral geometry outliers	2		
	Waters with clashes	0/0	0.00%	See UnDowser table for details

In the two column results, the left column gives the raw count, right column gives the percentage.
* 100th percentile is the best among structures of comparable resolution; 0th percentile is the worst. For clashscore the comparative set of structures was selected in 2004, for MolProbity score in 2006.
^ MolProbity score combines the clashscore, rotamer, and Ramachandran evaluations into a single score, normalized to be on the same scale as X-ray resolution.
Key to table colors and cutoffs here:

Figure S12. Quality report for 2NZ2 (ASS) obtained from MolProbity.

Summary statistics

All-Atom Contacts	Clashscore, all atoms:	5.85		98th percentile* (N=456, 2.20Å ± 0.25Å)
	Clashscore is the number of serious steric overlaps (> 0.4 Å) per 1000 atoms.			
Protein Geometry	Poor rotamers	12	1.64%	Goal: <0.3%
	Favored rotamers	695	95.21%	Goal: >98%
	Ramachandran outliers	1	0.12%	Goal: <0.05%
	Ramachandran favored	800	95.47%	Goal: >98%
	Rama distribution Z-score	-1.05 ± 0.27		Goal: abs(Z score) < 2
	MolProbity score^	1.80		93rd percentile* (N=10167, 2.20Å ± 0.25Å)
	Cβ deviations >0.25Å	0	0.00%	Goal: 0
	Bad bonds:	10 / 7170	0.14%	Goal: 0%
	Bad angles:	12 / 9764	0.12%	Goal: <0.1%
Peptide Omegas	Cis Prolines:	2 / 48	4.17%	Expected: ≤1 per chain, or ≤5%
Additional validations	Tetrahedral geometry outliers	2		
	Waters with clashes	0/0	0.00%	See UnDowser table for details

In the two column results, the left column gives the raw count, right column gives the percentage.
* 100th percentile is the best among structures of comparable resolution; 0th percentile is the worst. For clashscore the comparative set of structures was selected in 2004, for MolProbity score in 2006.
^ MolProbity score combines the clashscore, rotamer, and Ramachandran evaluations into a single score, normalized to be on the same scale as X-ray resolution.
Key to table colors and cutoffs here:

Figure S13. Quality report for 3E7G (iNOS) obtained from MolProbity.

Summary statistics

All-Atom Contacts	Clashscore, all atoms:	2.31		99th percentile* (N=816, 1.82Å ± 0.25Å)
	Clashscore is the number of serious steric overlaps (> 0.4 Å) per 1000 atoms.			
Protein Geometry	Poor rotamers	10	1.45%	Goal: <0.3%
	Favored rotamers	664	95.95%	Goal: >98%
	Ramachandran outliers	1	0.13%	Goal: <0.05%
	Ramachandran favored	783	98.49%	Goal: >98%
	Rama distribution Z-score	0.53 ± 0.27		Goal: abs(Z score) < 2
	MolProbity score^	1.13		99th percentile* (N=11325, 1.82Å ± 0.25Å)
	Cβ deviations >0.25Å	0	0.00%	Goal: 0
	Bad bonds:	2 / 6798	0.03%	Goal: 0%
	Bad angles:	4 / 9310	0.04%	Goal: <0.1%
Peptide Omegas	Cis Prolines:	2 / 58	3.45%	Expected: ≤1 per chain, or ≤5%
Additional validations	Chiral volume outliers	0/970		
	Waters with clashes	0/0	0.00%	See UnDowser table for details

In the two column results, the left column gives the raw count, right column gives the percentage.
* 100th percentile is the best among structures of comparable resolution; 0th percentile is the worst. For clashscore the comparative set of structures was selected in 2004, for MolProbity score in 2006.
^ MolProbity score combines the clashscore, rotamer, and Ramachandran evaluations into a single score, normalized to be on the same scale as X-ray resolution.
Key to table colors and cutoffs here: ?

Figure S14. Quality report for 4D1O (eNOS) obtained from MolProbity.

Summary statistics

All-Atom Contacts	Clashscore, all atoms:	2.46		100th percentile* (N=269, 2.48Å ± 0.25Å)
	Clashscore is the number of serious steric overlaps (> 0.4 Å) per 1000 atoms.			
Protein Geometry	Poor rotamers	5	0.81%	Goal: <0.3%
	Favored rotamers	592	96.42%	Goal: >98%
	Ramachandran outliers	0	0.00%	Goal: <0.05%
	Ramachandran favored	636	96.95%	Goal: >98%
	Rama distribution Z-score	-0.49 ± 0.31		Goal: abs(Z score) < 2
	MolProbity score^	1.28		100th percentile* (N=6912, 2.48Å ± 0.25Å)
	Cβ deviations >0.25Å	0	0.00%	Goal: 0
	Bad bonds:	0 / 5698	0.00%	Goal: 0%
	Bad angles:	0 / 7736	0.00%	Goal: <0.1%
Peptide Omegas	Cis Prolines:	2 / 18	11.11%	Expected: ≤1 per chain, or ≤5%
Additional validations	Chiral volume outliers	0/846		
	Waters with clashes	0/0	0.00%	See UnDowser table for details

In the two column results, the left column gives the raw count, right column gives the percentage.
* 100th percentile is the best among structures of comparable resolution; 0th percentile is the worst. For clashscore the comparative set of structures was selected in 2004, for MolProbity score in 2006.
^ MolProbity score combines the clashscore, rotamer, and Ramachandran evaluations into a single score, normalized to be on the same scale as X-ray resolution.
Key to table colors and cutoffs here: ?

Figure S15. Quality report for 6NT2 (PRMT1) obtained from MolProbity.

Summary statistics

All-Atom Contacts	Clashscore, all atoms:	0.55		100th percentile* (N=336, 2.36Å ± 0.25Å)
	Clashscore is the number of serious steric overlaps (> 0.4 Å) per 1000 atoms.			
Protein Geometry	Poor rotamers	2	0.33%	Goal: <0.3%
	Favored rotamers	572	95.49%	Goal: >98%
	Ramachandran outliers	0	0.00%	Goal: <0.05%
	Ramachandran favored	654	96.04%	Goal: >98%
	Rama distribution Z-score	-0.77 ± 0.30		Goal: abs(Z score) < 2
	MolProbity score^	0.96		100th percentile* (N=8043, 2.36Å ± 0.25Å)
	Cβ deviations >0.25Å	0	0.00%	Goal: 0
	Bad bonds:	0 / 5648	0.00%	Goal: 0%
	Bad angles:	0 / 7658	0.00%	Goal: <0.1%
Peptide Omegas	Cis Prolines:	2 / 26	7.69%	Expected: ≤1 per chain, or ≤5%
Additional validations	Chiral volume outliers	0/839		
	Waters with clashes	0/0	0.00%	See UnDowser table for details

In the two column results, the left column gives the raw count, right column gives the percentage.
* 100th percentile is the best among structures of comparable resolution; 0th percentile is the worst. For clashscore the comparative set of structures was selected in 2004, for MolProbity score in 2006.
^ MolProbity score combines the clashscore, rotamer, and Ramachandran evaluations into a single score, normalized to be on the same scale as X-ray resolution.
Key to table colors and cutoffs here: ?

Figure S16. Quality report for 5DWQ (PRMT4) obtained from MolProbity.

Summary statistics

All-Atom Contacts	Clashscore, all atoms:	5.25		97th percentile* (N=677, 2.03Å ± 0.25Å)
	Clashscore is the number of serious steric overlaps (> 0.4 Å) per 1000 atoms.			
Protein Geometry	Poor rotamers	3	1.32%	Goal: <0.3%
	Favored rotamers	222	97.80%	Goal: >98%
	Ramachandran outliers	0	0.00%	Goal: <0.05%
	Ramachandran favored	261	95.60%	Goal: >98%
	Rama distribution Z-score	-1.60 ± 0.44		Goal: abs(Z score) < 2
	MolProbity score^	1.68		92nd percentile* (N=12152, 2.03Å ± 0.25Å)
	Cβ deviations >0.25Å	0	0.00%	Goal: 0
	Bad bonds:	0 / 2121	0.00%	Goal: 0%
	Bad angles:	0 / 2870	0.00%	Goal: <0.1%
Peptide Omegas	Cis Prolines:	0 / 12	0.00%	Expected: ≤1 per chain, or ≤5%
Additional validations	Chiral volume outliers	0/337		
	Waters with clashes	0/0	0.00%	See UnDowser table for details

In the two column results, the left column gives the raw count, right column gives the percentage.
* 100th percentile is the best among structures of comparable resolution; 0th percentile is the worst. For clashscore the comparative set of structures was selected in 2004, for MolProbity score in 2006.
^ MolProbity score combines the clashscore, rotamer, and Ramachandran evaluations into a single score, normalized to be on the same scale as X-ray resolution.
Key to table colors and cutoffs here: ?

Figure S17. Quality report for 3I2E (DDAH1) obtained from MolProbity.

Summary statistics

All-Atom Contacts	Clashscore, all atoms:	2.55		99th percentile* (N=791, 1.66Å ± 0.25Å)
	Clashscore is the number of serious steric overlaps (> 0.4 Å) per 1000 atoms.			
Protein Geometry	Poor rotamers	7	1.02%	Goal: <0.3%
	Favored rotamers	654	95.75%	Goal: >98%
	Ramachandran outliers	0	0.00%	Goal: <0.05%
	Ramachandran favored	779	98.48%	Goal: >98%
	Rama distribution Z-score	0.20 ± 0.29		Goal: abs(Z score) < 2
	MolProbity score^	1.05		100th percentile* (N=8776, 1.66Å ± 0.25Å)
	Cβ deviations >0.25Å	0	0.00%	Goal: 0
	Bad bonds:	1 / 6442	0.02%	Goal: 0%
	Bad angles:	2 / 8722	0.02%	Goal: <0.1%
Peptide Omegas	Cis Prolines:	0 / 35	0.00%	Expected: ≤1 per chain, or ≤5%
Additional validations	Chiral volume outliers	0/965		
	Waters with clashes	0/0	0.00%	See UnDowser table for details

In the two column results, the left column gives the raw count, right column gives the percentage.
* 100th percentile is the best among structures of comparable resolution; 0th percentile is the worst. For clashscore the comparative set of structures was selected in 2004, for MolProbity score in 2006.
^ MolProbity score combines the clashscore, rotamer, and Ramachandran evaluations into a single score, normalized to be on the same scale as X-ray resolution.
Key to table colors and cutoffs here: ?

Figure S18. Quality report for 7S3G (ODC) obtained from MolProbity.

Summary statistics

All-Atom Contacts	Clashscore, all atoms:	2.53		100th percentile* (N=457, 2.21Å ± 0.25Å)
	Clashscore is the number of serious steric overlaps (> 0.4 Å) per 1000 atoms.			
Protein Geometry	Poor rotamers	1	0.38%	Goal: <0.3%
	Favored rotamers	244	92.08%	Goal: >98%
	Ramachandran outliers	1	0.33%	Goal: <0.05%
	Ramachandran favored	297	97.70%	Goal: >98%
	Rama distribution Z-score	0.40 ± 0.45		Goal: abs(Z score) < 2
	MolProbity score^	1.09		100th percentile* (N=10183, 2.21Å ± 0.25Å)
	Cβ deviations >0.25Å	0	0.00%	Goal: 0
	Bad bonds:	0 / 2433	0.00%	Goal: 0%
	Bad angles:	0 / 3323	0.00%	Goal: <0.1%
Peptide Omegas	Cis Prolines:	0 / 18	0.00%	Expected: ≤1 per chain, or ≤5%
	Cis nonProlines:	1 / 295	0.34%	Goal: <0.05%
Additional validations	Chiral volume outliers	0/382		
	Waters with clashes	0/0	0.00%	See UnDowser table for details

In the two column results, the left column gives the raw count, right column gives the percentage.
* 100th percentile is the best among structures of comparable resolution; 0th percentile is the worst. For clashscore the comparative set of structures was selected in 2004, for MolProbity score in 2006.
^ MolProbity score combines the clashscore, rotamer, and Ramachandran evaluations into a single score, normalized to be on the same scale as X-ray resolution.
Key to table colors and cutoffs here: ?

Figure S19. Quality report for 6Q37 (ARG2) obtained from MolProbity.

Summary statistics

All-Atom Contacts	Clashscore, all atoms:	1.62		100th percentile* (N=677, 2.03Å ± 0.25Å)
	Clashscore is the number of serious steric overlaps (> 0.4 Å) per 1000 atoms.			
Protein Geometry	Poor rotamers	10	2.65%	Goal: <0.3%
	Favored rotamers	352	93.37%	Goal: >98%
	Ramachandran outliers	0	0.00%	Goal: <0.05%
	Ramachandran favored	410	98.09%	Goal: >98%
	Rama distribution Z-score	-0.39 ± 0.38		Goal: abs(Z score) < 2
	MolProbity score^	1.23		99th percentile* (N=12152, 2.03Å ± 0.25Å)
	Cβ deviations >0.25Å	1	0.25%	Goal: 0
	Bad bonds:	0 / 3541	0.00%	Goal: 0%
	Bad angles:	1 / 4808	0.02%	Goal: <0.1%
Peptide Omegas	Cis Prolines:	1 / 24	4.17%	Expected: ≤1 per chain, or ≤5%
Additional validations	Chiral volume outliers	0/505		
	Waters with clashes	0/0	0.00%	See UnDowser table for details

In the two column results, the left column gives the raw count, right column gives the percentage.

* 100th percentile is the best among structures of comparable resolution; 0th percentile is the worst. For clashscore the comparative set of structures was selected in 2004, for MolProbity score in 2006.

^ MolProbity score combines the clashscore, rotamer, and Ramachandran evaluations into a single score, normalized to be on the same scale as X-ray resolution.

Key to table colors and cutoffs here:

Figure S20. Quality report for 4D1N (nNOS) obtained from MolProbity.

Summary statistics

All-Atom Contacts	Clashscore, all atoms:	8.1		98th percentile* (N=189, 2.70Å ± 0.25Å)
	Clashscore is the number of serious steric overlaps (> 0.4 Å) per 1000 atoms.			
Protein Geometry	Poor rotamers	21	6.40%	Goal: <0.3%
	Favored rotamers	290	88.41%	Goal: >98%
	Ramachandran outliers	4	1.06%	Goal: <0.05%
	Ramachandran favored	360	95.49%	Goal: >98%
	Rama distribution Z-score	-1.53 ± 0.37		Goal: abs(Z score) < 2
	MolProbity score^	2.37		91st percentile* (N=5412, 2.70Å ± 0.25Å)
	Cβ deviations >0.25Å	2	0.57%	Goal: 0
	Bad bonds:	0 / 3099	0.00%	Goal: 0%
	Bad angles:	2 / 4191	0.05%	Goal: <0.1%
Peptide Omegas	Cis Prolines:	1 / 19	5.26%	Expected: ≤1 per chain, or ≤5%
Low-resolution Criteria	CaBLAM outliers	14	3.7%	Goal: <1.0%
	CA Geometry outliers	2	0.53%	Goal: <0.5%
Additional validations	Chiral volume outliers	0/453		
	Waters with clashes	3/46	6.52%	See UnDowser table for details

In the two column results, the left column gives the raw count, right column gives the percentage.

* 100th percentile is the best among structures of comparable resolution; 0th percentile is the worst. For clashscore the comparative set of structures was selected in 2004, for MolProbity score in 2006.

^ MolProbity score combines the clashscore, rotamer, and Ramachandran evaluations into a single score, normalized to be on the same scale as X-ray resolution.

Key to table colors and cutoffs here:

Figure S21. Quality report for 1QK1 (CKU) obtained from MolProbity.

Summary statistics

All-Atom Contacts	Clashscore, all atoms:	54.24		45th percentile* (N=37, 3Å - 9999Å)
	Clashscore is the number of serious steric overlaps (> 0.4 Å) per 1000 atoms.			
Protein Geometry	Poor rotamers	41	12.89%	Goal: <0.3%
	Favored rotamers	235	73.90%	Goal: >98%
	Ramachandran outliers	16	4.43%	Goal: <0.05%
	Ramachandran favored	281	77.84%	Goal: >98%
	Rama distribution Z-score	-6.08 ± 0.29		Goal: abs(Z score) < 2
	MolProbity score^	3.81		46th percentile* (N=342, 3.50Å ± 0.25Å)
	Cβ deviations >0.25Å	0	0.00%	Goal: 0
	Bad bonds:	0 / 2965	0.00%	Goal: 0%
	Bad angles:	1 / 3994	0.03%	Goal: <0.1%
Peptide Omegas	Cis Prolines:	1 / 18	5.56%	Expected: ≤1 per chain, or ≤5%
Low-resolution Criteria	CaBLAM outliers	15	4.2%	Goal: <1.0%
	CA Geometry outliers	6	1.68%	Goal: <0.5%
Additional validations	Chiral volume outliers	0/428		
	Waters with clashes	0/0	0.00%	See UnDowser table for details

In the two column results, the left column gives the raw count, right column gives the percentage.

* 100th percentile is the best among structures of comparable resolution; 0th percentile is the worst. For clashscore the comparative set of structures was selected in 2004, for MolProbity score in 2006.

^ MolProbity score combines the clashscore, rotamer, and Ramachandran evaluations into a single score, normalized to be on the same scale as X-ray resolution.

Key to table colors and cutoffs here:

Figure S22. Quality report for 1I0E (CKM) obtained from MolProbity.

Summary statistics

All-Atom Contacts	Clashscore, all atoms:	1.67		100th percentile* (N=777, 1.86Å ± 0.25Å)
	Clashscore is the number of serious steric overlaps (> 0.4 Å) per 1000 atoms.			
Protein Geometry	Poor rotamers	0	0.00%	Goal: <0.3%
	Favored rotamers	182	96.30%	Goal: >98%
	Ramachandran outliers	0	0.00%	Goal: <0.05%
	Ramachandran favored	219	96.05%	Goal: >98%
	Rama distribution Z-score	-0.11 ± 0.55		Goal: abs(Z score) < 2
	MolProbity score^	1.19		99th percentile* (N=11957, 1.86Å ± 0.25Å)
	Cβ deviations >0.25Å	0	0.00%	Goal: 0
	Bad bonds:	1 / 1873	0.05%	Goal: 0%
	Bad angles:	3 / 2554	0.12%	Goal: <0.1%
Peptide Omegas	Cis Prolines:	0 / 18	0.00%	Expected: ≤1 per chain, or ≤5%
Additional validations	Chiral volume outliers	0/272		
	Waters with clashes	0/0	0.00%	See UnDowser table for details

In the two column results, the left column gives the raw count, right column gives the percentage.

* 100th percentile is the best among structures of comparable resolution; 0th percentile is the worst. For clashscore the comparative set of structures was selected in 2004, for MolProbity score in 2006.

^ MolProbity score combines the clashscore, rotamer, and Ramachandran evaluations into a single score, normalized to be on the same scale as X-ray resolution.

Key to table colors and cutoffs here:

Figure S23. Quality report for 3ORH (GAMT) obtained from MolProbity.

Summary statistics

All-Atom Contacts	Clashscore, all atoms:	4.3		99th percentile* (N=576, 2.10Å ± 0.25Å)
	Clashscore is the number of serious steric overlaps (> 0.4 Å) per 1000 atoms.			
Protein Geometry	Poor rotamers	7	2.19%	Goal: <0.3%
	Favored rotamers	297	93.10%	Goal: >98%
	Ramachandran outliers	0	0.00%	Goal: <0.05%
	Ramachandran favored	340	94.97%	Goal: >98%
	Rama distribution Z-score	-1.54 ± 0.41		Goal: abs(Z score) < 2
	MolProbity score^	1.82		90th percentile* (N=11758, 2.10Å ± 0.25Å)
	Cβ deviations >0.25Å	2	0.58%	Goal: 0
	Bad bonds:	0 / 3027	0.00%	Goal: 0%
	Bad angles:	1 / 4116	0.02%	Goal: <0.1%
Peptide Omegas	Cis Prolines:	1 / 27	3.70%	Expected: ≤1 per chain, or ≤5%
Additional validations	Chiral volume outliers	0/433		
	Waters with clashes	4/196	2.04%	See UnDowser table for details

In the two column results, the left column gives the raw count, right column gives the percentage.

* 100th percentile is the best among structures of comparable resolution; 0th percentile is the worst. For clashscore the comparative set of structures was selected in 2004, for MolProbity score in 2006.

^ MolProbity score combines the clashscore, rotamer, and Ramachandran evaluations into a single score, normalized to be on the same scale as X-ray resolution.

Key to table colors and cutoffs here:

Figure S24. Quality report for 2JDW (AGAT) obtained from MolProbity.

Summary statistics

All-Atom Contacts	Clashscore, all atoms:	2.79		99th percentile* (N=837, 1.80Å ± 0.25Å)
	Clashscore is the number of serious steric overlaps (> 0.4 Å) per 1000 atoms.			
Protein Geometry	Poor rotamers	0	0.00%	Goal: <0.3%
	Favored rotamers	250	96.15%	Goal: >98%
	Ramachandran outliers	0	0.00%	Goal: <0.05%
	Ramachandran favored	291	99.32%	Goal: >98%
	Rama distribution Z-score	-0.10 ± 0.47		Goal: abs(Z score) < 2
	MolProbity score^	1.07		100th percentile* (N=11444, 1.80Å ± 0.25Å)
	Cβ deviations >0.25Å	0	0.00%	Goal: 0
	Bad bonds:	0 / 2399	0.00%	Goal: 0%
	Bad angles:	1 / 3277	0.03%	Goal: <0.1%
Peptide Omegas	Cis Prolines:	0 / 22	0.00%	Expected: ≤1 per chain, or ≤5%
Additional validations	Chiral volume outliers	0/389		
	Waters with clashes	0/0	0.00%	See UnDowser table for details

In the two column results, the left column gives the raw count, right column gives the percentage.

* 100th percentile is the best among structures of comparable resolution; 0th percentile is the worst. For clashscore the comparative set of structures was selected in 2004, for MolProbity score in 2006.

^ MolProbity score combines the clashscore, rotamer, and Ramachandran evaluations into a single score, normalized to be on the same scale as X-ray resolution.

Key to table colors and cutoffs here:

Figure S25. Quality report for 5I2C (CASTOR1) obtained from MolProbity.

Summary statistics

All-Atom Contacts	Clashscore, all atoms:	2.29		99th percentile* (N=575, 2.09Å ± 0.25Å)
	Clashscore is the number of serious steric overlaps (> 0.4 Å) per 1000 atoms.			
Protein Geometry	Poor rotamers	5	0.81%	Goal: <0.3%
	Favored rotamers	590	95.62%	Goal: >98%
	Ramachandran outliers	0	0.00%	Goal: <0.05%
	Ramachandran favored	692	97.74%	Goal: >98%
	Rama distribution Z-score	-0.59 ± 0.28		Goal: abs(Z score) < 2
	MolProbity score^	1.07		100th percentile* (N=11618, 2.09Å ± 0.25Å)
	Cβ deviations >0.25Å	0	0.00%	Goal: 0
	Bad bonds:	0 / 6033	0.00%	Goal: 0%
	Bad angles:	0 / 8283	0.00%	Goal: <0.1%
Peptide Omegas	Cis Prolines:	4 / 57	7.02%	Expected: ≤1 per chain, or ≤5%
Additional validations	Chiral volume outliers	0/874		
	Waters with clashes	0/0	0.00%	See UnDowser table for details

In the two column results, the left column gives the raw count, right column gives the percentage.
* 100th percentile is the best among structures of comparable resolution; 0th percentile is the worst. For clashscore the comparative set of structures was selected in 2004, for MolProbity score in 2006.
^ MolProbity score combines the clashscore, rotamer, and Ramachandran evaluations into a single score, normalized to be on the same scale as X-ray resolution.
Key to table colors and cutoffs here:

Figure S26. Quality report for 3HIG (DAO) obtained from MolProbity.

Summary statistics

All-Atom Contacts	Clashscore, all atoms:	8.37		89th percentile* (N=715, 2.00Å ± 0.25Å)
	Clashscore is the number of serious steric overlaps (> 0.4 Å) per 1000 atoms.			
Protein Geometry	Poor rotamers	10	3.11%	Goal: <0.3%
	Favored rotamers	304	94.41%	Goal: >98%
	Ramachandran outliers	1	0.27%	Goal: <0.05%
	Ramachandran favored	367	98.13%	Goal: >98%
	Rama distribution Z-score	-1.01 ± 0.37		Goal: abs(Z score) < 2
	MolProbity score^	1.83		85th percentile* (N=12522, 2.00Å ± 0.25Å)
	Cβ deviations >0.25Å	0	0.00%	Goal: 0
	Bad bonds:	0 / 3023	0.00%	Goal: 0%
	Bad angles:	1 / 4089	0.02%	Goal: <0.1%
Peptide Omegas	Cis Prolines:	1 / 19	5.26%	Expected: ≤1 per chain, or ≤5%
Additional validations	Chiral volume outliers	0/442		
	Waters with clashes	0/0	0.00%	See UnDowser table for details

In the two column results, the left column gives the raw count, right column gives the percentage.
* 100th percentile is the best among structures of comparable resolution; 0th percentile is the worst. For clashscore the comparative set of structures was selected in 2004, for MolProbity score in 2006.
^ MolProbity score combines the clashscore, rotamer, and Ramachandran evaluations into a single score, normalized to be on the same scale as X-ray resolution.
Key to table colors and cutoffs here:

Figure S27. Quality report for 3B6R (CKB) obtained from MolProbity.

Summary statistics

All-Atom Contacts	Clashscore, all atoms:	24.4		87th percentile* (N=41, 2.92Å - 9999Å)
	Clashscore is the number of serious steric overlaps (> 0.4 Å) per 1000 atoms.			
Protein Geometry	Poor rotamers	1	0.30%	Goal: <0.3%
	Favored rotamers	262	78.92%	Goal: >98%
	Ramachandran outliers	30	8.13%	Goal: <0.05%
	Ramachandran favored	305	82.66%	Goal: >98%
	Rama distribution Z-score	-2.76 ± 0.41		Goal: abs(Z score) < 2
	MolProbity score^	2.58		96th percentile* (N=1550, 3.17Å ± 0.25Å)
	Cβ deviations >0.25Å	0	0.00%	Goal: 0
	Bad bonds:	0 / 3145	0.00%	Goal: 0%
	Bad angles:	6 / 4275	0.14%	Goal: <0.1%
Peptide Omegas	Cis Prolines:	0 / 16	0.00%	Expected: ≤1 per chain, or ≤5%
Low-resolution Criteria	CaBLAM outliers	27	7.6%	Goal: <1.0%
	CA Geometry outliers	5	1.42%	Goal: <0.5%
Additional validations	Chiral volume outliers	0/498		
	Waters with clashes	0/0	0.00%	See UnDowser table for details

In the two column results, the left column gives the raw count, right column gives the percentage.
* 100th percentile is the best among structures of comparable resolution; 0th percentile is the worst. For clashscore the comparative set of structures was selected in 2004, for MolProbity score in 2006.
^ MolProbity score combines the clashscore, rotamer, and Ramachandran evaluations into a single score, normalized to be on the same scale as X-ray resolution.
Key to table colors and cutoffs here:

Figure S28. Quality report for 6C08 (SLC38A9) obtained from MolProbity.

Summary statistics

All-Atom Contacts	Clashscore, all atoms:	2.57		99th percentile* (N=715, 2.00Å ± 0.25Å)
	Clashscore is the number of serious steric overlaps (> 0.4 Å) per 1000 atoms.			
Protein Geometry	Poor rotamers	3	1.16%	Goal: <0.3%
	Favored rotamers	246	94.98%	Goal: >98%
	Ramachandran outliers	0	0.00%	Goal: <0.05%
	Ramachandran favored	288	97.63%	Goal: >98%
	Rama distribution Z-score	-0.46 ± 0.48		Goal: abs(Z score) < 2
	MolProbity score^	1.17		100th percentile* (N=12522, 2.00Å ± 0.25Å)
	Cβ deviations >0.25Å	2	0.71%	Goal: 0
	Bad bonds:	0 / 2408	0.00%	Goal: 0%
	Bad angles:	0 / 3268	0.00%	Goal: <0.1%
Peptide Omegas	Cis Prolines:	0 / 17	0.00%	Expected: ≤1 per chain, or ≤5%
Additional validations	Chiral volume outliers	0/356		
	Waters with clashes	0/0	0.00%	See UnDowser table for details

In the two column results, the left column gives the raw count, right column gives the percentage.
* 100th percentile is the best among structures of comparable resolution; 0th percentile is the worst. For clashscore the comparative set of structures was selected in 2004, for MolProbity score in 2006.
^ MolProbity score combines the clashscore, rotamer, and Ramachandran evaluations into a single score, normalized to be on the same scale as X-ray resolution.
Key to table colors and cutoffs here:

Figure S29. Quality report for 2O06 (SPDS) obtained from MolProbity.

Summary statistics

All-Atom Contacts	Clashscore, all atoms:	3.91		99th percentile* (N=821, 1.95Å ± 0.25Å)
	Clashscore is the number of serious steric overlaps (> 0.4 Å) per 1000 atoms.			
Protein Geometry	Poor rotamers	10	3.37%	Goal: <0.3%
	Favored rotamers	271	91.25%	Goal: >98%
	Ramachandran outliers	0	0.00%	Goal: <0.05%
	Ramachandran favored	313	96.90%	Goal: >98%
	Rama distribution Z-score	-0.24 ± 0.44		Goal: abs(Z score) < 2
	MolProbity score^	1.76		86th percentile* (N=13349, 1.95Å ± 0.25Å)
	Cβ deviations >0.25Å	3	0.97%	Goal: 0
	Bad bonds:	0 / 2727	0.00%	Goal: 0%
	Bad angles:	0 / 3677	0.00%	Goal: <0.1%
Peptide Omegas	Cis Prolines:	0 / 10	0.00%	Expected: ≤1 per chain, or ≤5%
Additional validations	Chiral volume outliers	0/414		
	Waters with clashes	0/0	0.00%	See UnDowser table for details

In the two column results, the left column gives the raw count, right column gives the percentage.
* 100th percentile is the best among structures of comparable resolution; 0th percentile is the worst. For clashscore the comparative set of structures was selected in 2004, for MolProbity score in 2006.
^ MolProbity score combines the clashscore, rotamer, and Ramachandran evaluations into a single score, normalized to be on the same scale as X-ray resolution.
Key to table colors and cutoffs here:

Figure S30. Quality report for 3C6K (SPMS) obtained from MolProbity.

As can be observed, some parameters were bad for different targets according to the quality reports obtained from MolProbity. For this reason, we decided to perform a minimization of energy in UCSF Chimera in the low-quality targets, aiming to improve some parameters before the molecular docking simulations. We performed the minimizations using 1000 steepest descent steps in the minimize structure tool. The quality reports after the minimization of energy of proteins are shown below:

Summary statistics

All-Atom Contacts	Clashscore, all atoms:	0.2		100th percentile* (N=773, 1.90Å ± 0.25Å)
	Clashscore is the number of serious steric overlaps (> 0.4 Å) per 1000 atoms.			
Protein Geometry	Poor rotamers	12	4.33%	Goal: <0.3%
	Favored rotamers	252	90.97%	Goal: >98%
	Ramachandran outliers	3	0.94%	Goal: <0.05%
	Ramachandran favored	309	96.87%	Goal: >98%
	Rama distribution Z-score	-2.75 ± 0.42		Goal: abs(Z score) < 2
	MolProbity score^	1.25		99th percentile* (N=12147, 1.90Å ± 0.25Å)
	Cβ deviations >0.25Å	0	0.00%	Goal: 0
	Bad bonds:	0 / 2589	0.00%	Goal: 0%
	Bad angles:	25 / 3501	0.71%	Goal: <0.1%
Peptide Omegas	Cis Prolines:	1 / 13	7.69%	Expected: ≤1 per chain, or ≤5%
	Twisted Peptides:	1 / 320	0.31%	Goal: 0
Additional validations	Chiral volume outliers	0/398		
	Waters with clashes	0/0	0.00%	See UnDowser table for details

In the two column results, the left column gives the raw count, right column gives the percentage.
* 100th percentile is the best among structures of comparable resolution; 0th percentile is the worst. For clashscore the comparative set of structures was selected in 2004, for MolProbity score in 2006.
^ MolProbity score combines the clashscore, rotamer, and Ramachandran evaluations into a single score, normalized to be on the same scale as X-ray resolution.
Key to table colors and cutoffs here:

Figure S31. Quality report for 1C9Y (OTC) after the energy minimization obtained from MolProbity.

Summary statistics

All-Atom Contacts	Clashscore, all atoms:	0		100th percentile* (N=331, 2.40Å ± 0.25Å)
	Clashscore is the number of serious steric overlaps (> 0.4 Å) per 1000 atoms.			
Protein Geometry	Poor rotamers	16	4.64%	Goal: <0.3%
	Favored rotamers	299	86.67%	Goal: >98%
	Ramachandran outliers	4	1.01%	Goal: <0.05%
	Ramachandran favored	373	94.19%	Goal: >98%
	Rama distribution Z-score	-2.35 ± 0.36		Goal: abs(Z score) < 2
	MolProbity score^	1.40		100th percentile* (N=8058, 2.40Å ± 0.25Å)
	Cβ deviations >0.25Å	4	1.08%	Goal: 0
	Bad bonds:	0 / 3280	0.00%	Goal: 0%
	Bad angles:	23 / 4431	0.52%	Goal: <0.1%
Peptide Omegas	Cis Prolines:	0 / 20	0.00%	Expected: ≤1 per chain, or ≤5%
	Twisted Peptides:	3 / 399	0.75%	Goal: 0
Additional validations	Chiral volume outliers	0/479		
	Waters with clashes	0/0	0.00%	See UnDowser table for details

In the two column results, the left column gives the raw count, right column gives the percentage.
* 100th percentile is the best among structures of comparable resolution; 0th percentile is the worst. For clashscore the comparative set of structures was selected in 2004, for MolProbity score in 2006.
^ MolProbity score combines the clashscore, rotamer, and Ramachandran evaluations into a single score, normalized to be on the same scale as X-ray resolution.
Key to table colors and cutoffs here:

Figure S32. Quality report for 2NZ2 (ASS) after the energy minimization obtained from MolProbity.

Summary statistics

All-Atom Contacts	Clashscore, all atoms:	0.36		100th percentile* (N=456, 2.20Å ± 0.25Å)
	Clashscore is the number of serious steric overlaps (> 0.4 Å) per 1000 atoms.			
Protein Geometry	Poor rotamers	9	1.23%	Goal: <0.3%
	Favored rotamers	705	96.05%	Goal: >98%
	Ramachandran outliers	0	0.00%	Goal: <0.05%
	Ramachandran favored	808	96.42%	Goal: >98%
	Rama distribution Z-score	-0.65 ± 0.28		Goal: abs(Z score) < 2
	MolProbity score^	0.93		100th percentile* (N=10167, 2.20Å ± 0.25Å)
	Cβ deviations >0.25Å	0	0.00%	Goal: 0
	Bad bonds:	9 / 7188	0.13%	Goal: 0%
	Bad angles:	60 / 9786	0.61%	Goal: <0.1%
Peptide Omegas	Cis Prolines:	2 / 48	4.17%	Expected: ≤1 per chain, or ≤5%
	Twisted Peptides:	1 / 840	0.12%	Goal: 0
Additional validations	Chiral handedness swaps	1/880	0.11%	See Chiral volume report for details
	Waters with clashes	0/0	0.00%	See UnDowser table for details

In the two column results, the left column gives the raw count, right column gives the percentage.
* 100th percentile is the best among structures of comparable resolution; 0th percentile is the worst. For clashscore the comparative set of structures was selected in 2004, for MolProbity score in 2006.
^ MolProbity score combines the clashscore, rotamer, and Ramachandran evaluations into a single score, normalized to be on the same scale as X-ray resolution.
Key to table colors and cutoffs here:

Figure S33. Quality report for 3E7G (iNOS) after the energy minimization obtained from MolProbity.

Summary statistics

All-Atom Contacts	Clashscore, all atoms:	1.11		100th percentile* (N=777, 1.86Å ± 0.25Å)
	Clashscore is the number of serious steric overlaps (> 0.4 Å) per 1000 atoms.			
Protein Geometry	Poor rotamers	2	1.06%	Goal: <0.3%
	Favored rotamers	181	95.77%	Goal: >98%
	Ramachandran outliers	1	0.44%	Goal: <0.05%
	Ramachandran favored	217	95.18%	Goal: >98%
	Rama distribution Z-score	-1.91 ± 0.51		Goal: abs(Z score) < 2
	MolProbity score^	1.17		99th percentile* (N=11957, 1.86Å ± 0.25Å)
	Cβ deviations >0.25Å	0	0.00%	Goal: 0
	Bad bonds:	0 / 1875	0.00%	Goal: 0%
	Bad angles:	11 / 2558	0.43%	Goal: <0.1%
Peptide Omegas	Cis Prolines:	0 / 18	0.00%	Expected: ≤1 per chain, or ≤5%
Additional validations	Chiral volume outliers	0/273		
	Waters with clashes	0/0	0.00%	See UnDowser table for details

In the two column results, the left column gives the raw count, right column gives the percentage.
* 100th percentile is the best among structures of comparable resolution; 0th percentile is the worst. For clashscore the comparative set of structures was selected in 2004, for MolProbity score in 2006.
^ MolProbity score combines the clashscore, rotamer, and Ramachandran evaluations into a single score, normalized to be on the same scale as X-ray resolution.
Key to table colors and cutoffs here:

Figure S34. Quality report for 3ORH (GAMT) after the energy minimization obtained from MolProbity.

Summary statistics

All-Atom Contacts	Clashscore, all atoms:	1.54		100th percentile* (N=37, 3Å - 9999Å)
	Clashscore is the number of serious steric overlaps (> 0.4 Å) per 1000 atoms.			
Protein Geometry	Poor rotamers	30	9.23%	Goal: <0.3%
	Favored rotamers	249	76.62%	Goal: >98%
	Ramachandran outliers	1	0.28%	Goal: <0.05%
	Ramachandran favored	326	90.30%	Goal: >98%
	Rama distribution Z-score	-3.12 ± 0.37		Goal: abs(Z score) < 2
	MolProbity score^	2.17		100th percentile* (N=342, 3.50Å ± 0.25Å)
	Cβ deviations >0.25Å	8	2.38%	Goal: 0
	Bad bonds:	0 / 2991	0.00%	Goal: 0%
	Bad angles:	38 / 4027	0.94%	Goal: <0.1%
Peptide Omegas	Cis Prolines:	1 / 18	5.56%	Expected: ≤1 per chain, or ≤5%
	Twisted Peptides:	3 / 363	0.83%	Goal: 0
Low-resolution Criteria	CaBLAM outliers	15	4.2%	Goal: <1.0%
	CA Geometry outliers	3	0.84%	Goal: <0.5%
Additional validations	Chiral volume outliers	0/429		
	Waters with clashes	0/0	0.00%	See UnDowser table for details

In the two column results, the left column gives the raw count, right column gives the percentage.
* 100th percentile is the best among structures of comparable resolution; 0th percentile is the worst. For clashscore the comparative set of structures was selected in 2004, for MolProbity score in 2006.
^ MolProbity score combines the clashscore, rotamer, and Ramachandran evaluations into a single score, normalized to be on the same scale as X-ray resolution.
Key to table colors and cutoffs here:

Figure S35. Quality report for 1I0E (CKU) after the energy minimization obtained from MolProbity.

As can be observed from the quality reports, some parameters improved after the energy minimization in UCSF Chimera. After this, we decided to proceed to the identification of the docking site. It is noteworthy that despite the improvement in the parameters not being large, the clashscore, the poor rotamers, and the favored rotamers, among other parameters, were better after the energy minimization. We tried to increase the steepest descent steps to >1000, but contrary to our expectations, the quality became worse. For this reason, we decided to perform the molecular docking simulations with these targets and test their quality in the molecular docking validation process.

- **Identification of the docking site**

In order to perform the molecular docking simulations, we needed to establish our docking sites. For this purpose, we searched for amino acid residues present in the active sites of the selected targets. First, we performed the search in UniProt, where we collected the reported amino acid residues in the function section. Additionally, to better delimitate the active site, we performed the prediction of pockets for our targets in the DoGSiteScorer tool. We chose those pockets that accomplished two characteristics: 1) the pocket selected had to include the amino acid residues reported in UniProt, and 2) we selected the pocket with the best drug score. Below, we show the selected pockets for our candidate targets.

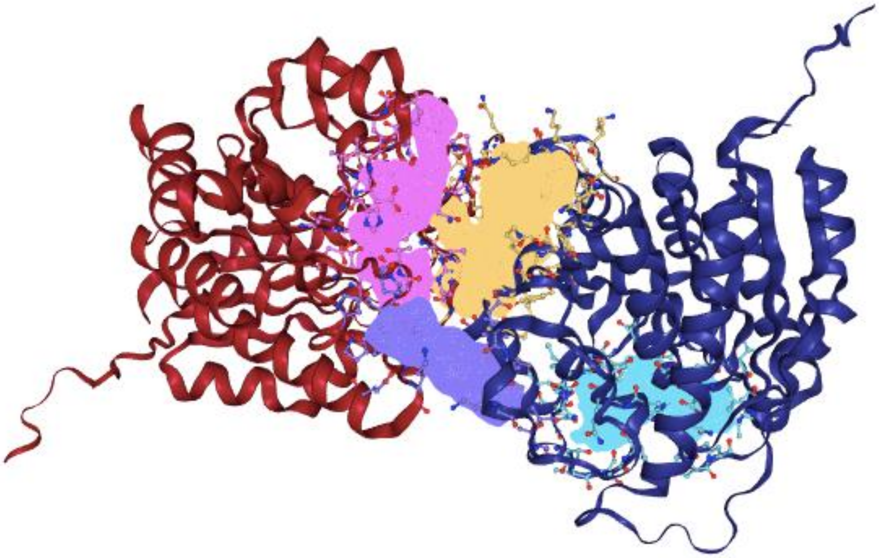

Figure S36. Predicted pockets for ARG1 using DoGSiteScorer.

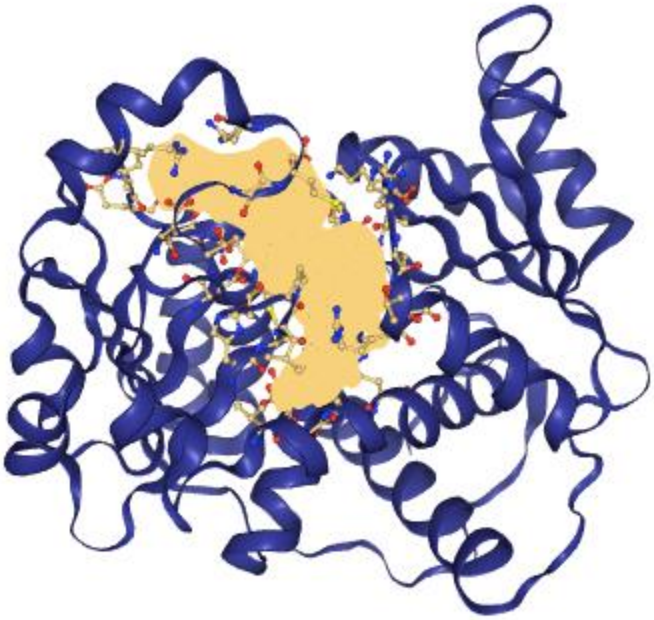

Figure S37. Predicted pockets for OTC using DoGSiteScorer.

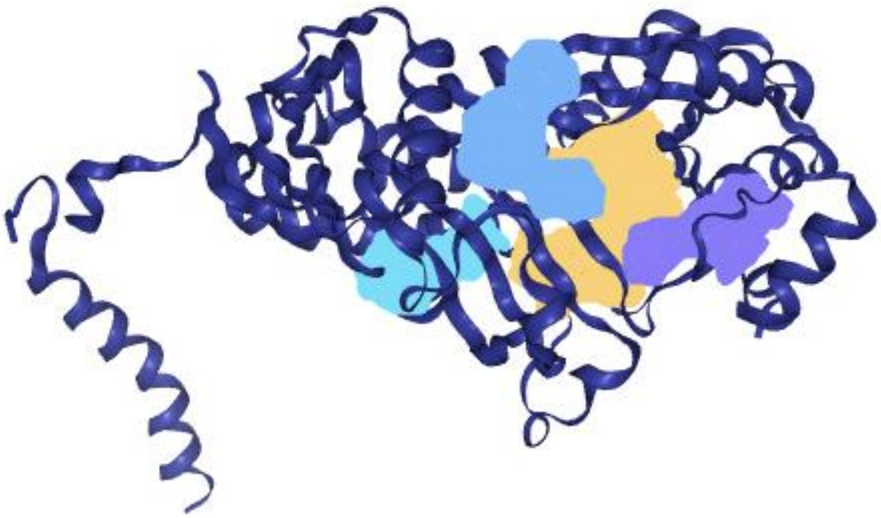

Figure S38. Predicted pockets for ASS using DoGSiteScorer.

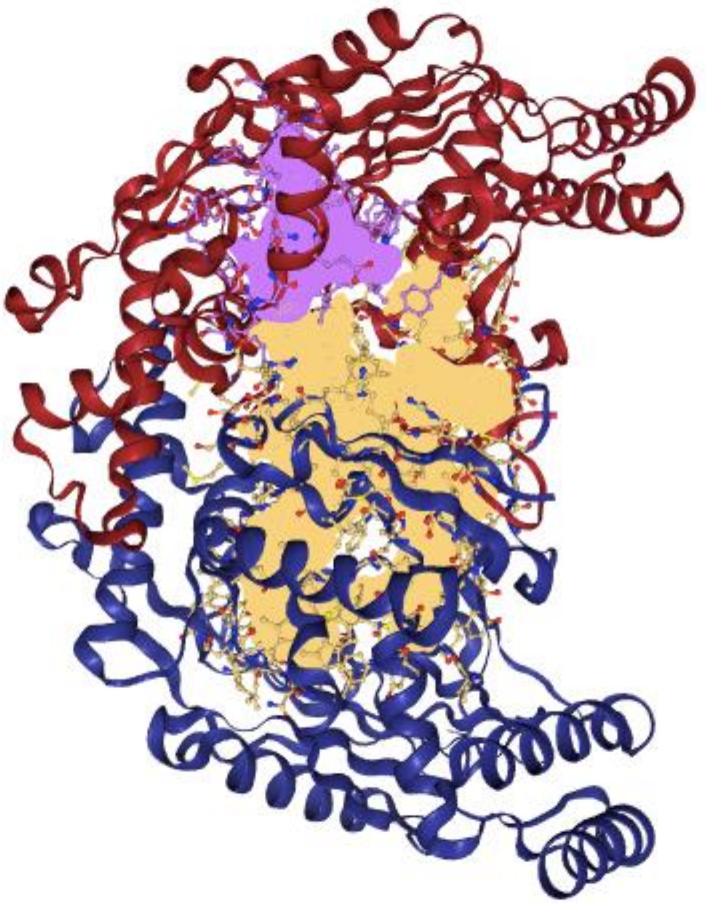

Figure S39. Predicted pockets for iNOS using DoGSiteScorer.

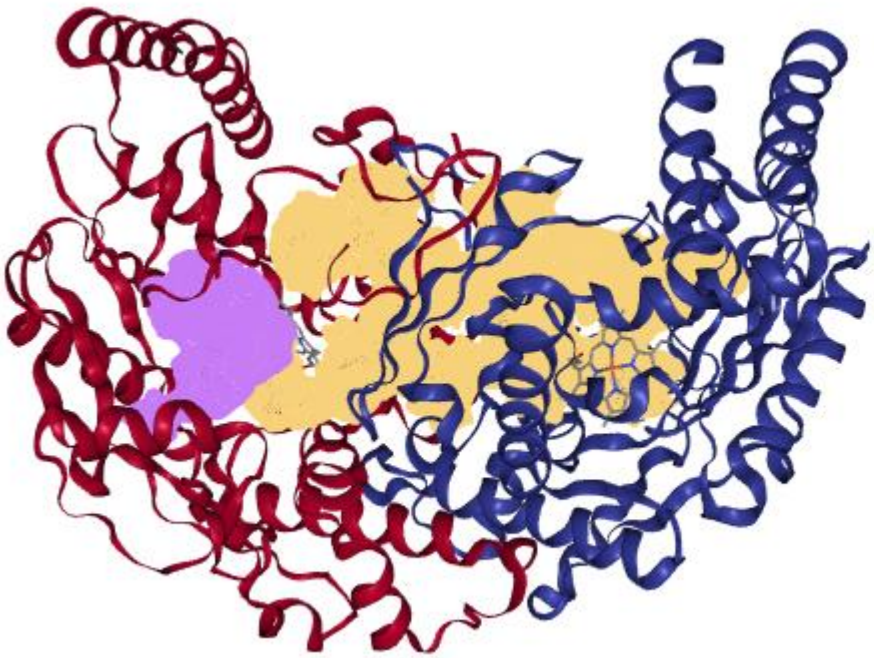

Figure S40. Predicted pockets for eNOS using DoGSiteScorer.

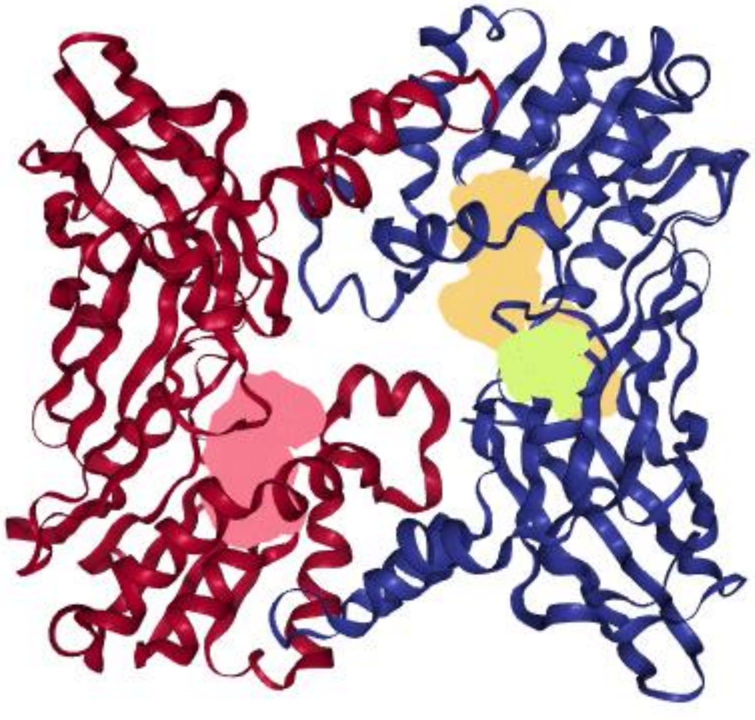

Figure S41. Predicted pockets for PRMT1 using DoGSiteScorer.

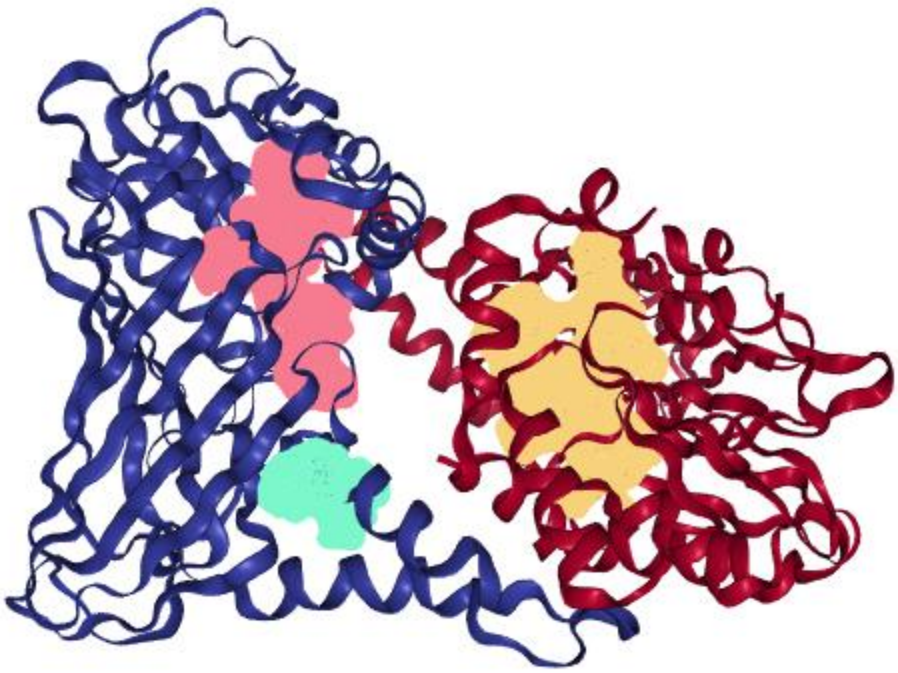

Figure S42. Predicted pockets for PRMT4 using DoGSiteScorer.

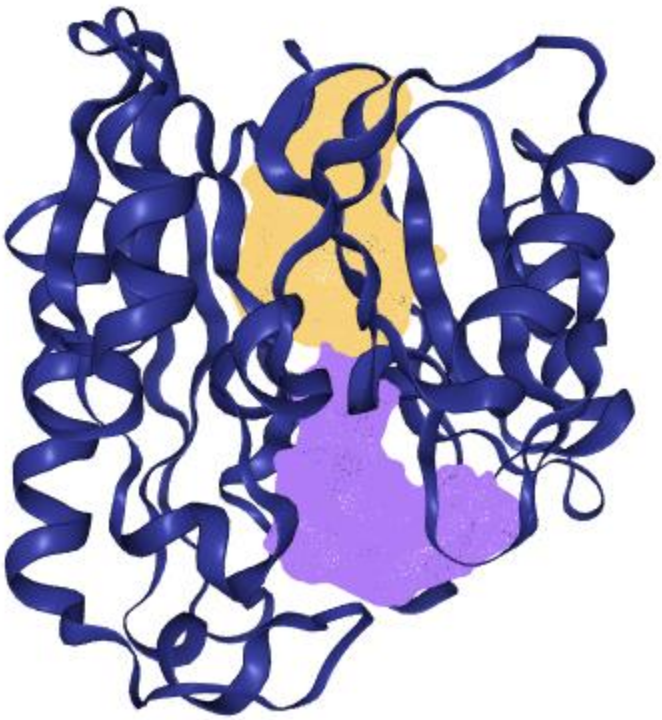

Figure S43. Predicted pockets for DDAH1 using DoGSiteScorer.

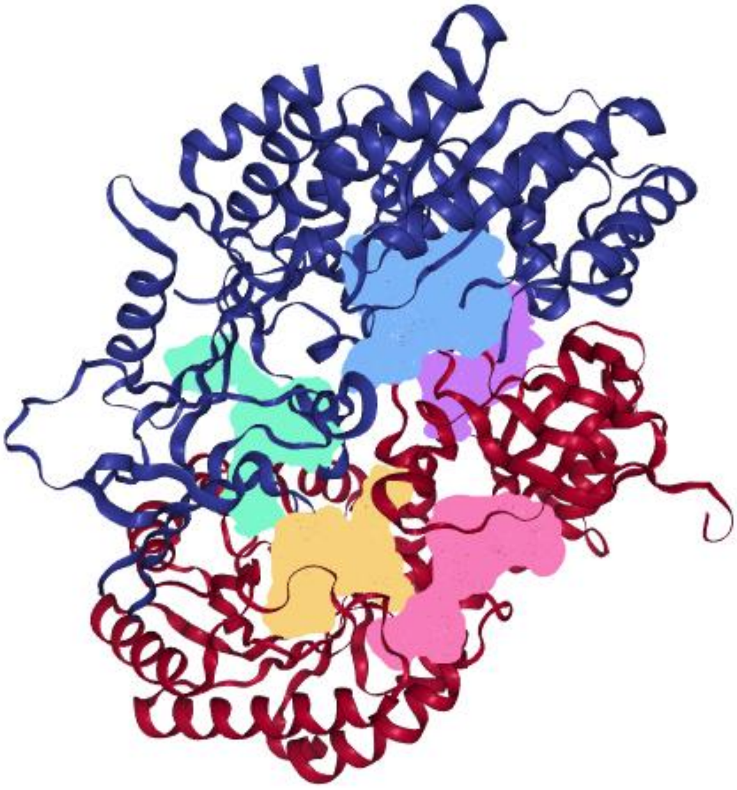

Figure S44. Predicted pockets for ODC using DoGSiteScorer.

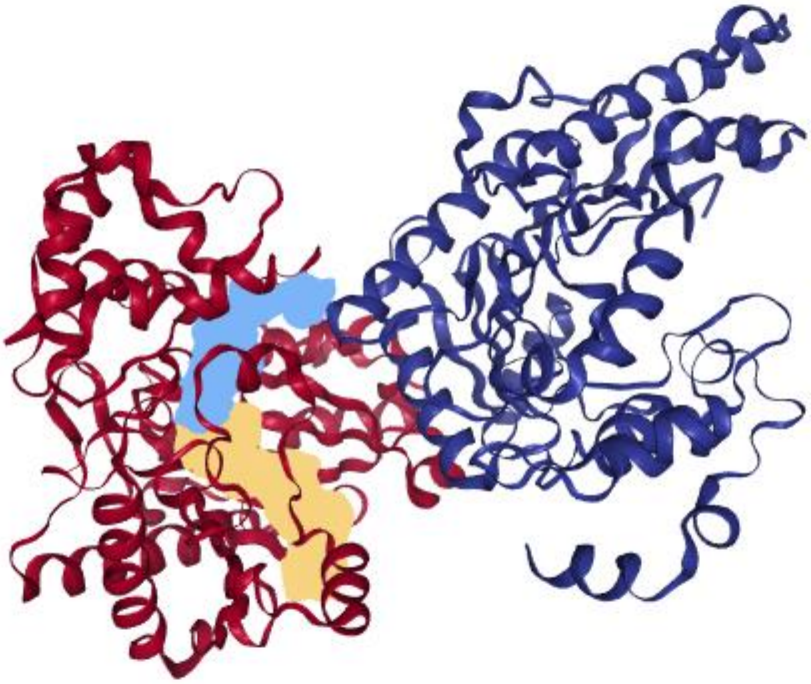

Figure S45. Predicted pockets for CKB using DoGSiteScorer.

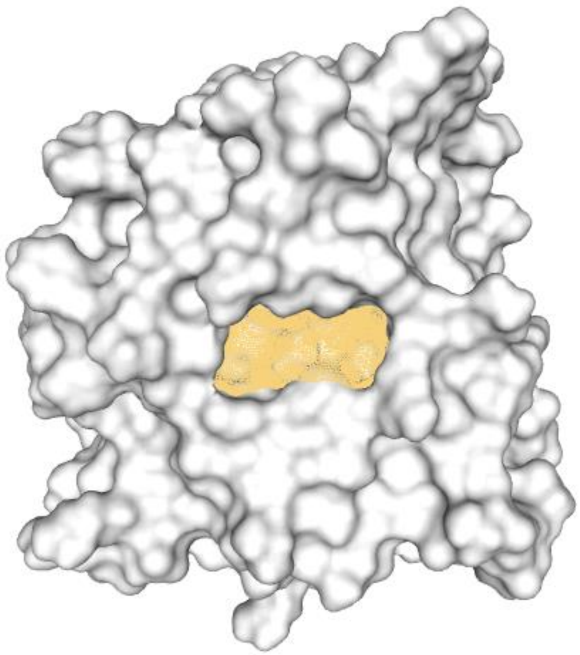

Figure S46. Predicted pockets for ARG2 using DoGSiteScorer.

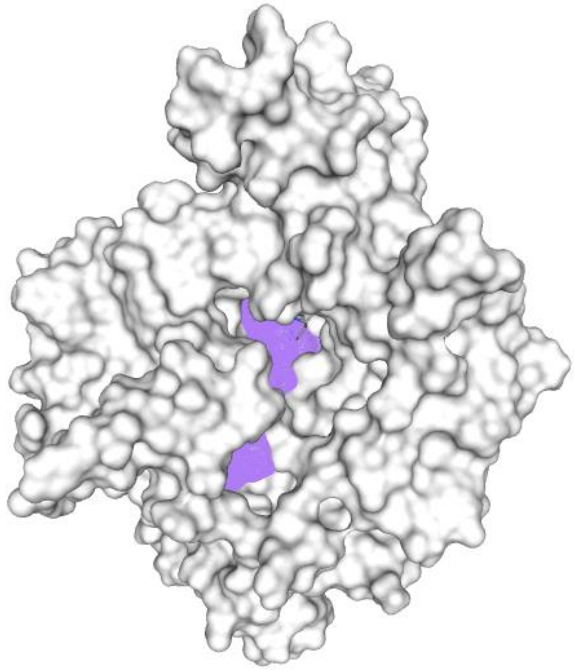

Figure S47. Predicted pockets for nNOS using DoGSiteScorer.

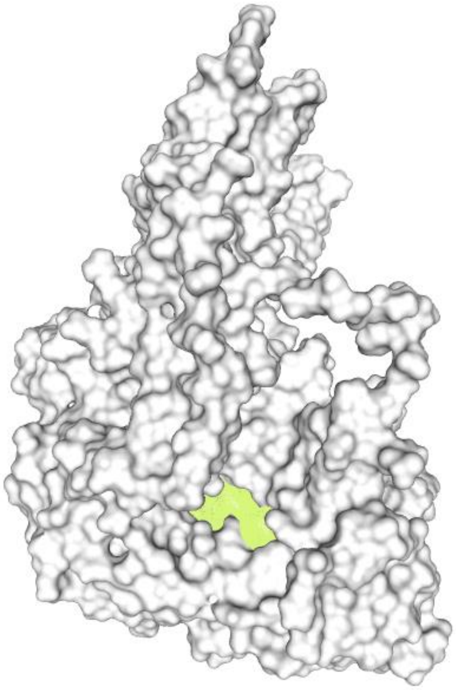

Figure S48. Predicted pockets for DAO using DoGSiteScorer.

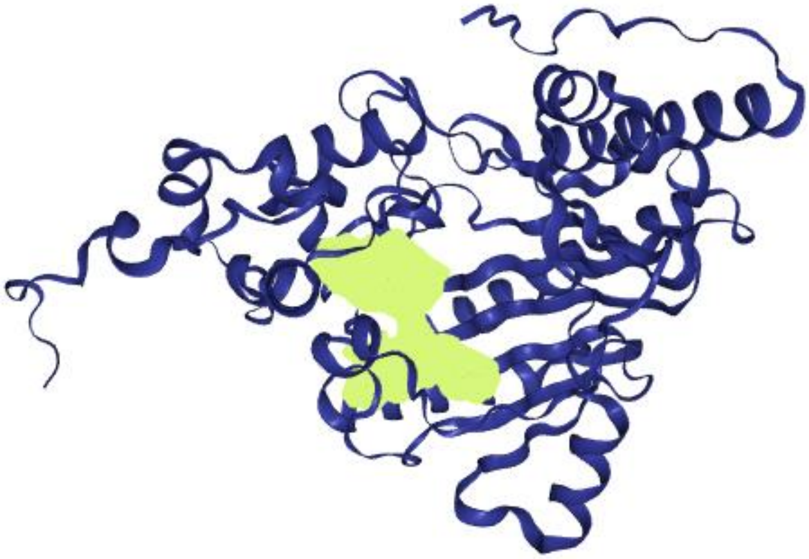

Figure S49. Predicted pockets for CKU using DoGSiteScorer.

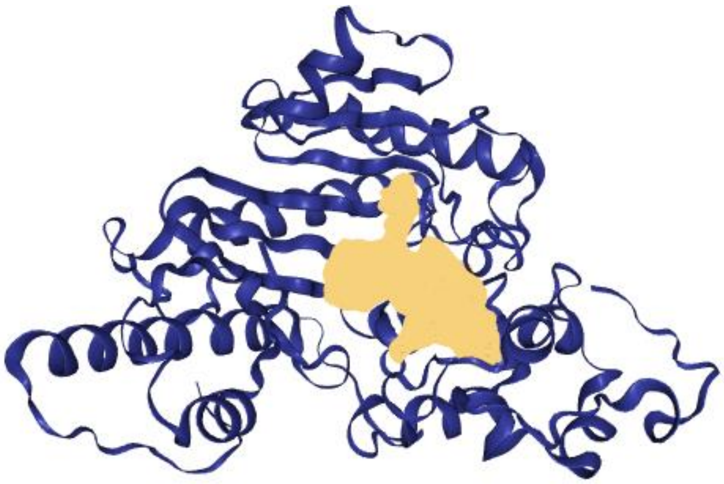

Figure S50. Predicted pockets for CKM using DoGSiteScorer.

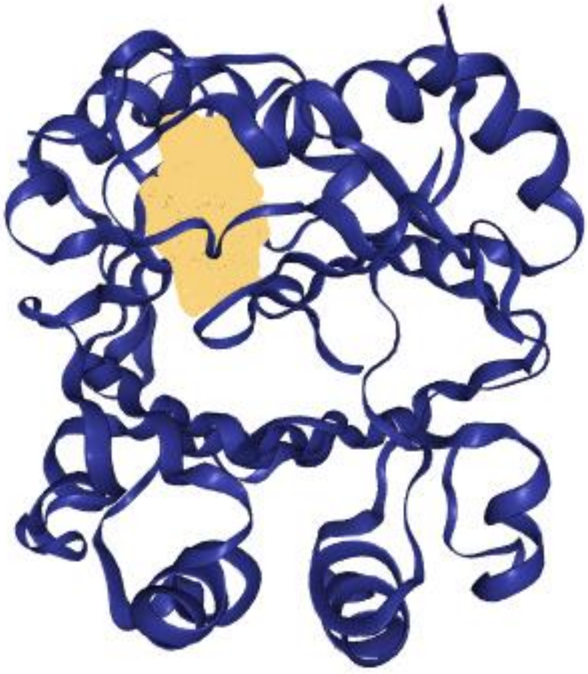

Figure S51. Predicted pockets for CASTOR1 using DoGSiteScorer.

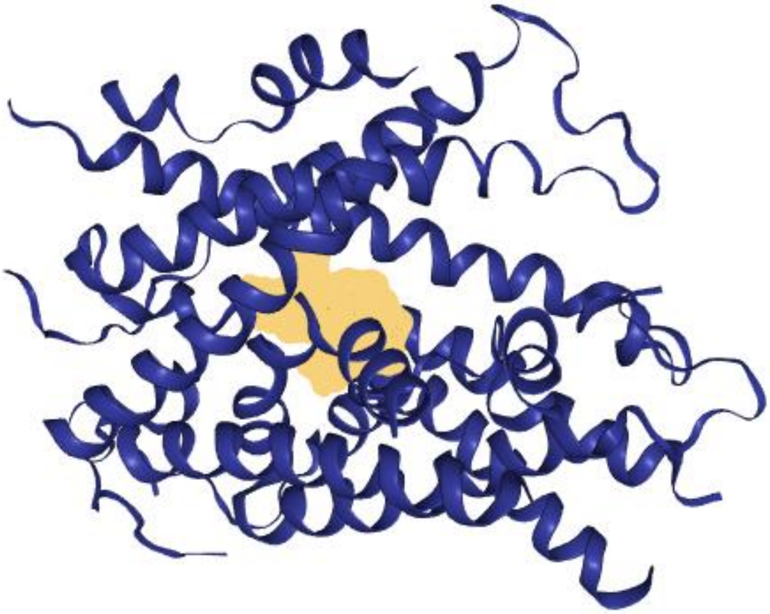

Figure S52. Predicted pockets for SLC38A9 using DoGSiteScorer.

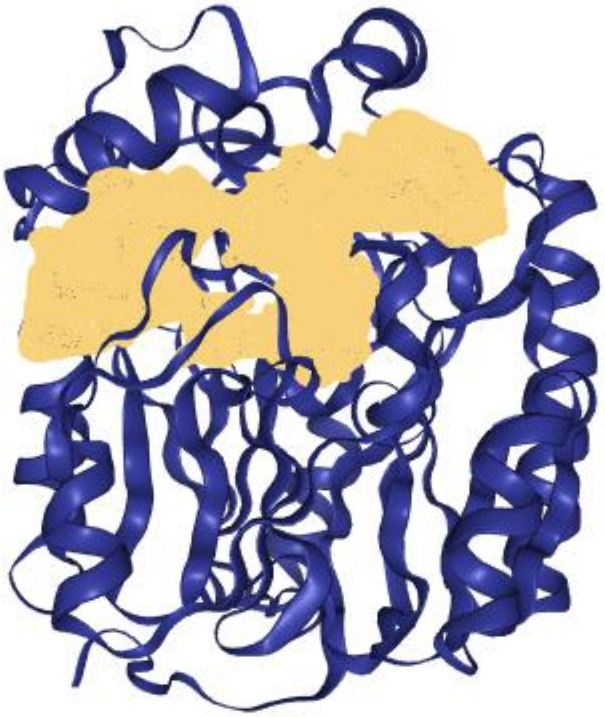

Figure S53. Predicted pockets for AGAT using DoGSiteScorer.

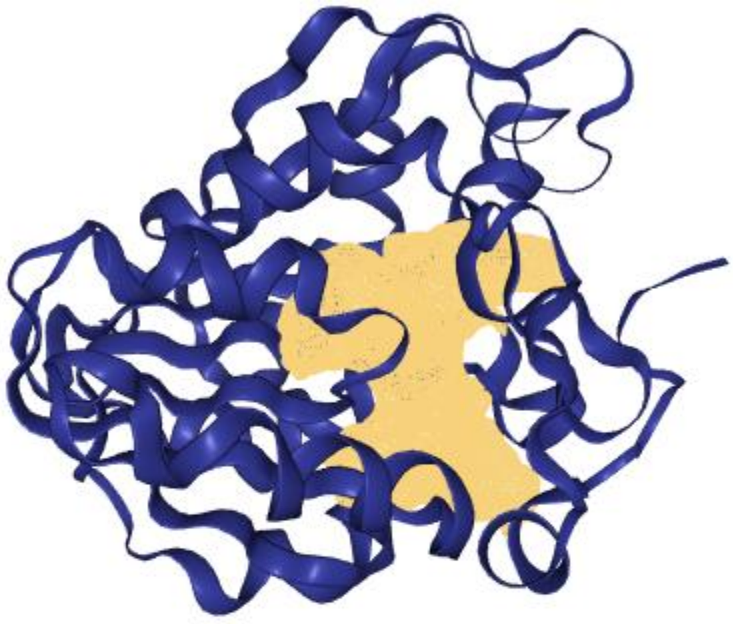

Figure S54. Predicted pockets for GAMT using DoGSiteScorer.

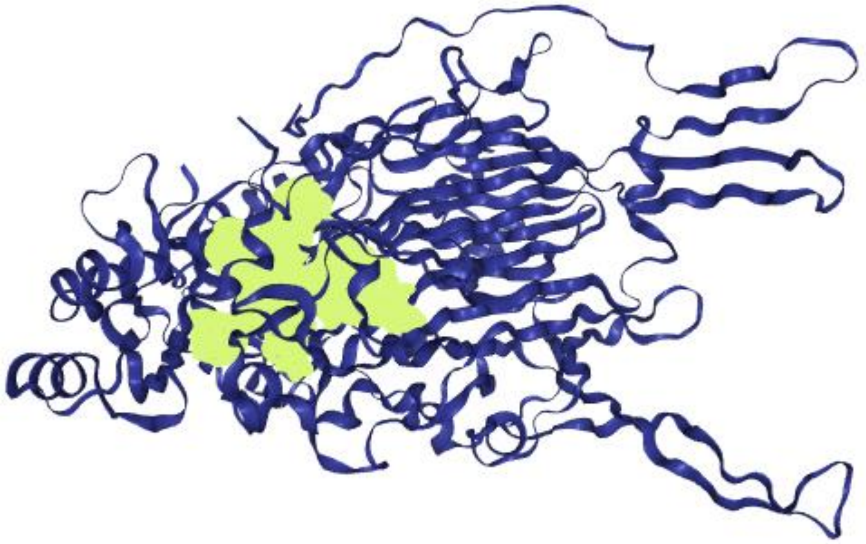

Figure S55. Predicted pockets for DAO using DoGSiteScorer.

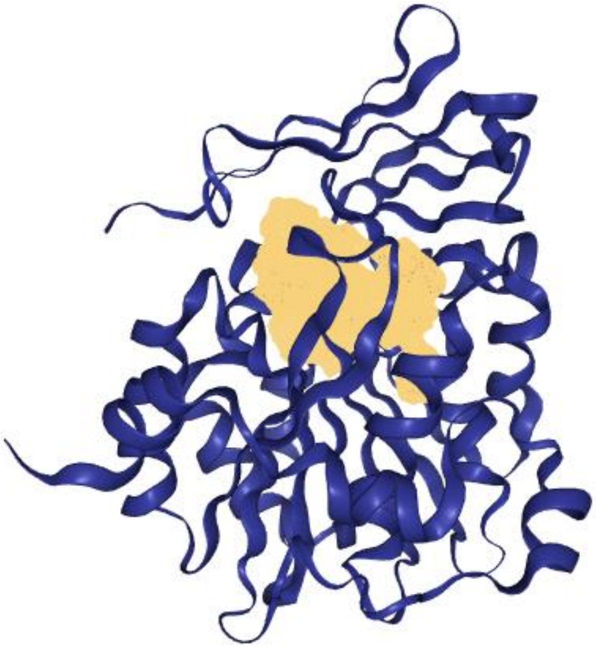

Figure S56. Predicted pockets for SPDS using DoGSiteScorer.

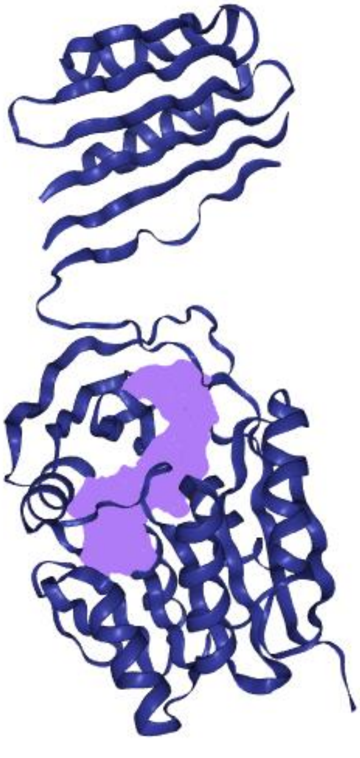

Figure S57. Predicted pockets for SPMS using DoGSiteScorer.

- **Validation of the molecular docking methodology**

Once we identified the docking site for our candidate targets, we performed the validation of the molecular docking methodology by employing the redocking method. In Supplementary Material part C, we report the

parameters employed in defining the grid box during the validation process. Our docking simulations were carried out in AutoDock 4.2 in a rigid modality. In the redocking method, we followed our established methodology, employing selected candidate targets and their corresponding crystallized ligands to perform the simulation. After the simulation, we performed a comparison between our simulated conformation and the crystallized conformation of the original ligand in UCSF Chimera. The coordinates employed in docking simulations are shown below, including the RMSD values in those validated targets.

Table S2. 3D coordinates and sizes for the grid boxes employed in the docking simulations

Target	Grid box Size			Grid box Coordinates			RMSD for the redocked ligand
	X	Y	Z	X	Y	Z	
ARG1	38	40	30	2.08	22.624	-11.323	NV
ARG2	34	34	34	35.198	89.007	68.774	4.603
OTC	30	20	20	3.923	2.994	-22.584	1.269
ASS	30	38	30	3.147	37.218	18.577	3.391
iNOS	24	26	38	56.623	20.285	79.908	2.107
eNOS	40	40	40	18.814	243.469	24.373	1.516
nNOS	36	34	36	230.156	28.582	11.324	2.08
PRMT1	40	40	40	-4.725	36.708	-14.904	1.083
PRMT4	40	40	40	-16.476	21.323	13.604	1.27
DDAH1	40	40	40	24.179	-5.728	45.845	NV
ODC	40	40	40	9.372	-3.213	60.512	3.245
CKB							
CKU	48	34	44	49.427	17.144	104.641	NV
CKM	58	44	54	1.013	15.828	91.566	NV
GAMT	40	48	40	64.371	62.13	14.016	1.249
AGAT	44	44	44	47.049	65.583	13.552	NV
CASTOR1	40	40	40	48.822	81.527	80.321	1.101
SLC38A9	28	28	28	-54.894	35.9	70.782	4.314
DAO	46	42	44	-32.828	-11.041	73.498	1.403
SPMS	20	32	20	6.611	67.642	0.787	1.667
SPDS	20	32	32	14.314	26.303	10.686	2.084

NV: Not validated.

Some targets could not be validated because their original PDB files did not include a ligand to perform the redocking method. However, we still selected the grid box based on UniProt information and DoGSiteScorer, performed the simulations, and compared our results with the scientific literature to test if our methodology was generating reliable results.

- **Binding modes of biguanides and candidate metabolites in the docked targets**

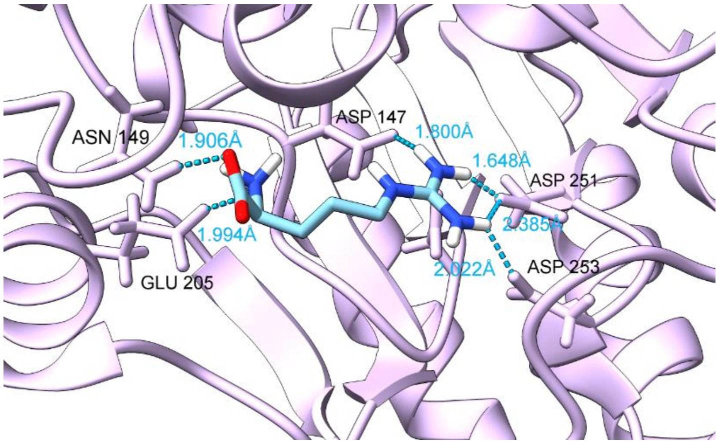

Figure S58. Predicted binding mode of L-arginine in ARG2.

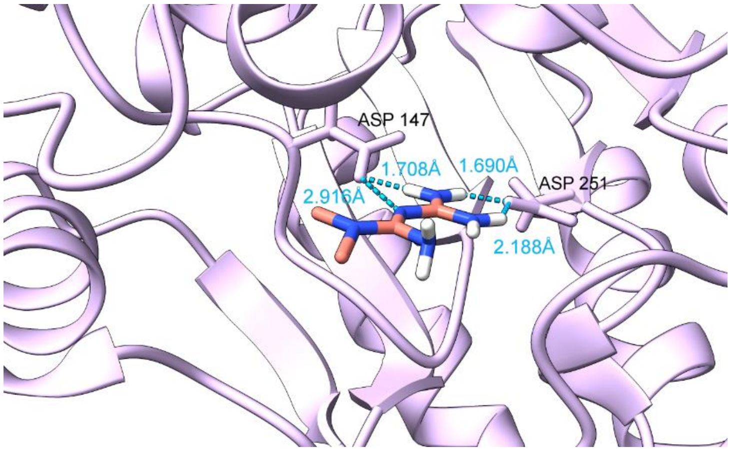

Figure S59. Predicted binding mode of metformin in ARG2.

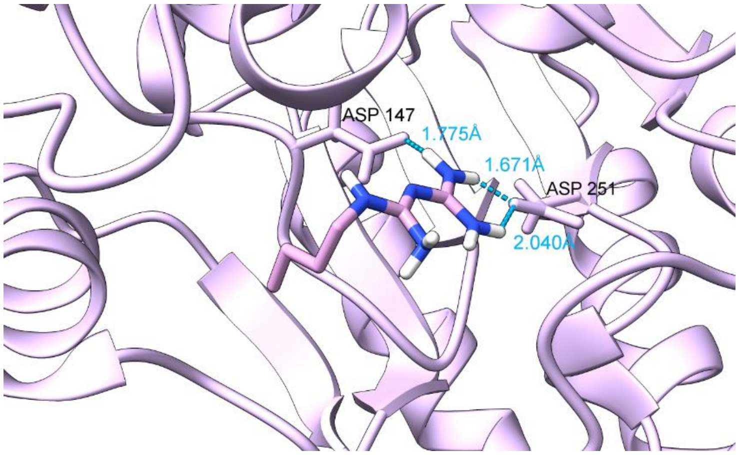

Figure S60. Predicted binding mode of buformin in ARG2.

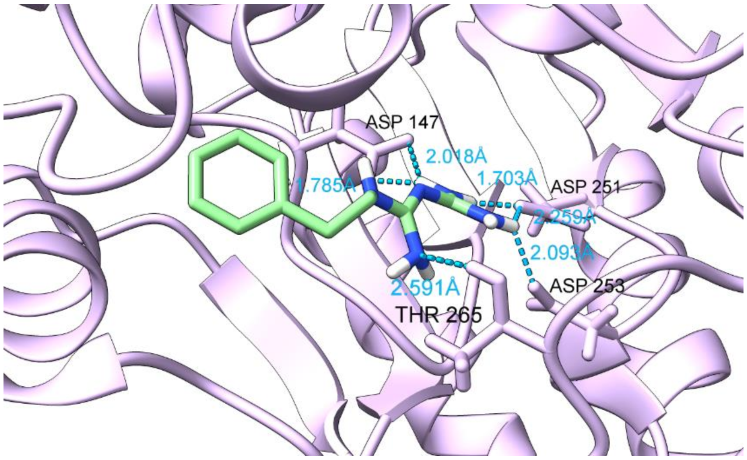

Figure S61. Predicted binding mode of phenformin in ARG2.

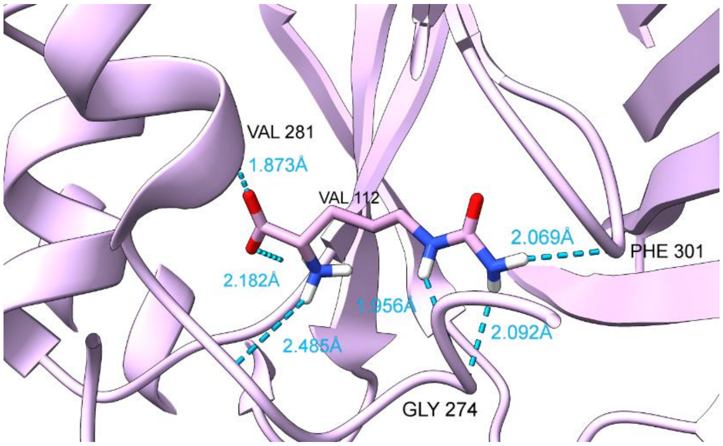

Figure S62. Predicted binding mode of L-citrulline in CASTOR1.

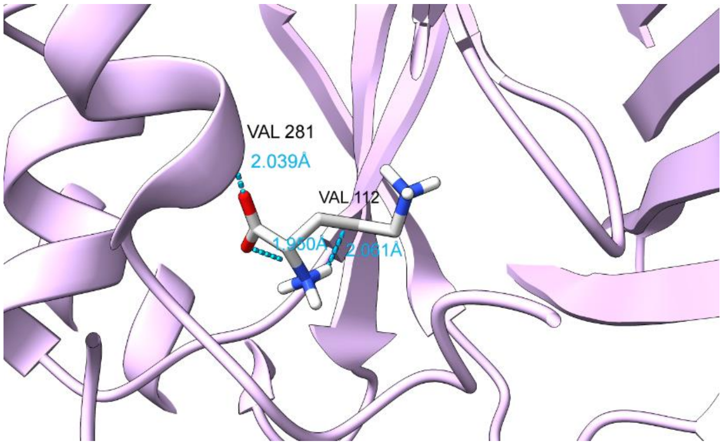

Figure S63. Predicted binding mode of L-ornithine in CASTOR1.

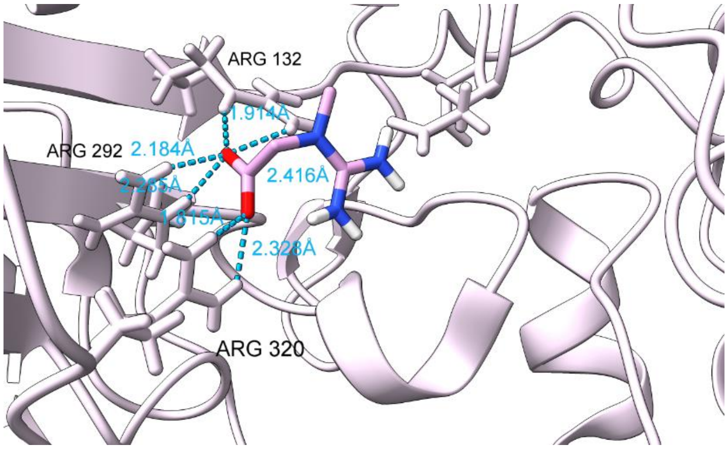

Figure S64. Predicted binding mode of creatine in M-type creatine kinase.

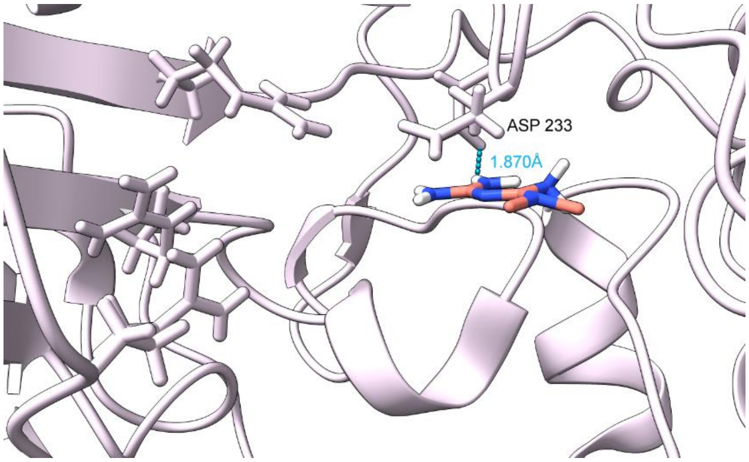

Figure S65. Predicted binding mode of metformin in M-type creatine kinase.

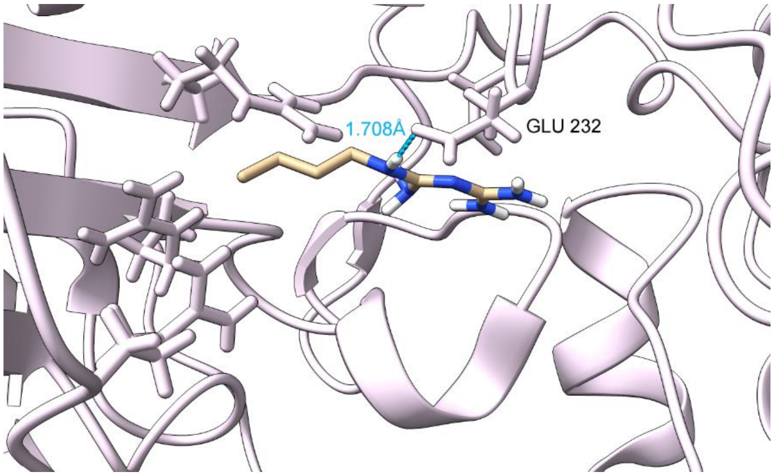

Figure S66. Predicted binding mode of buformin in M-type creatine kinase.

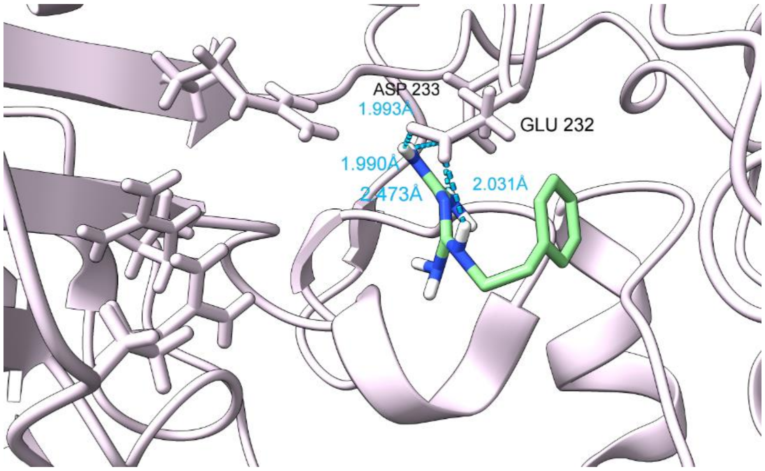

Figure S67. Predicted binding mode of phenformin in M-type creatine kinase.

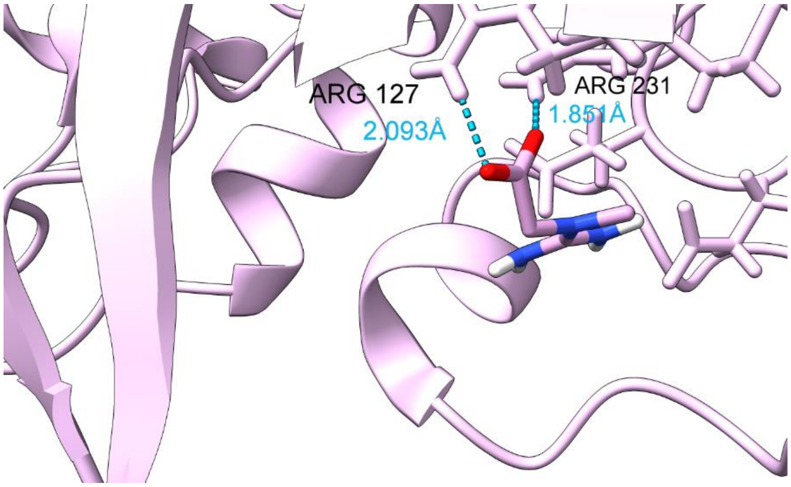

Figure S68. Predicted binding mode of creatine in the ubiquitous creatine kinase.

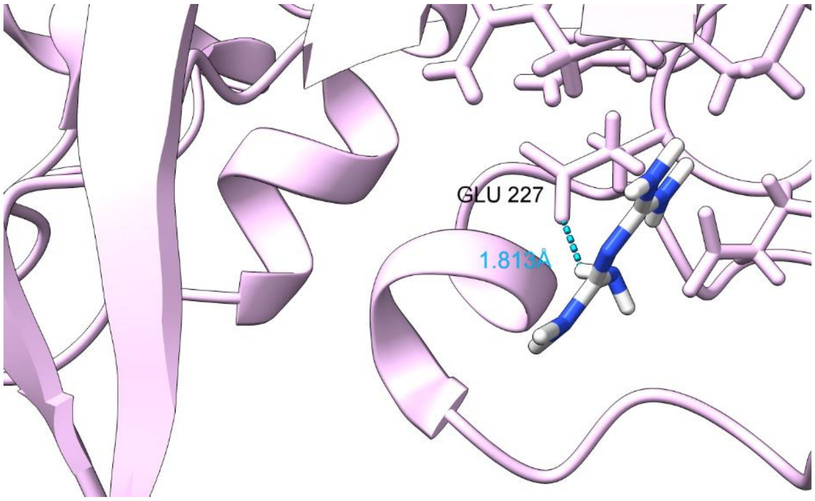

Figure S69. Predicted binding mode of metformin in the ubiquitous creatine kinase.

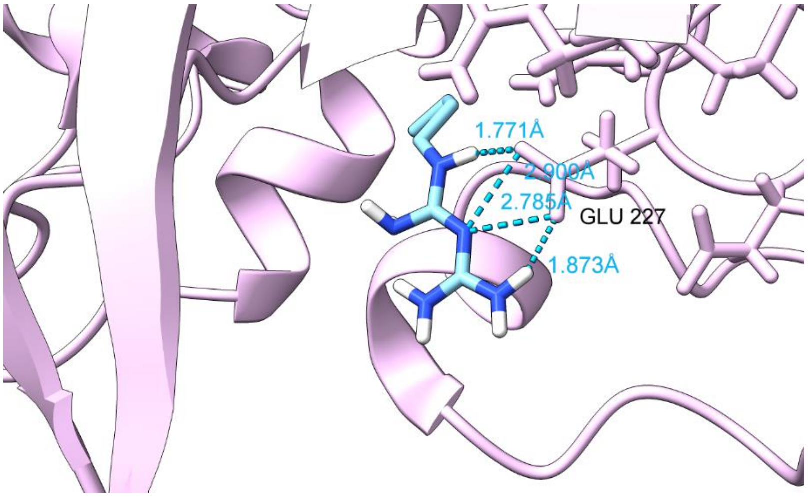

Figure S70. Predicted binding mode of buformin in the ubiquitous creatine kinase.

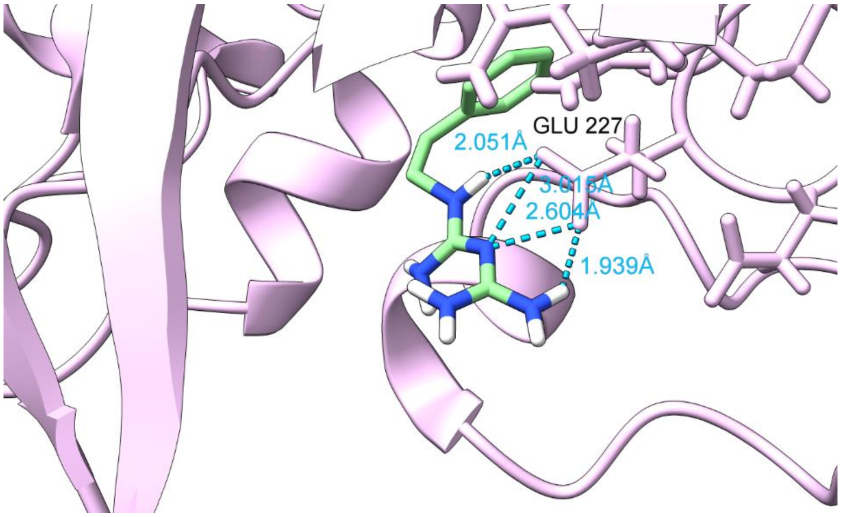

Figure S71. Predicted binding mode of phenformin in the ubiquitous creatine kinase.

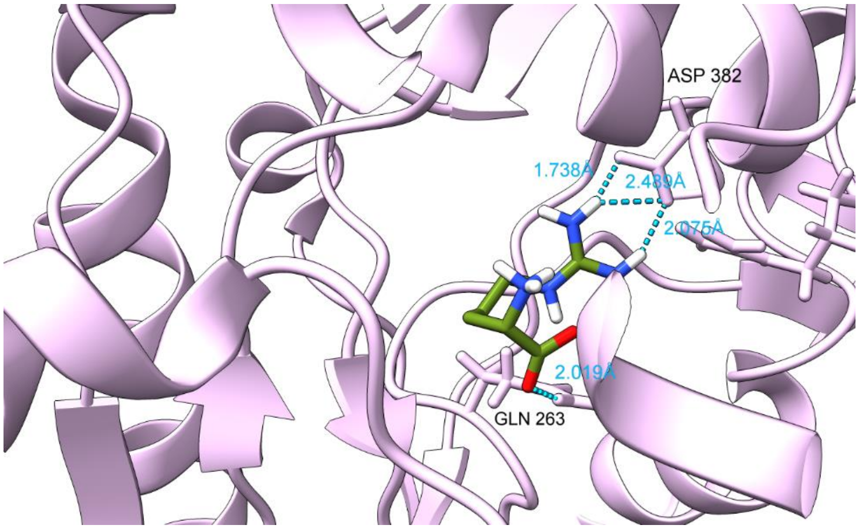

Figure S72. Predicted binding mode of L-arginine in iNOS.

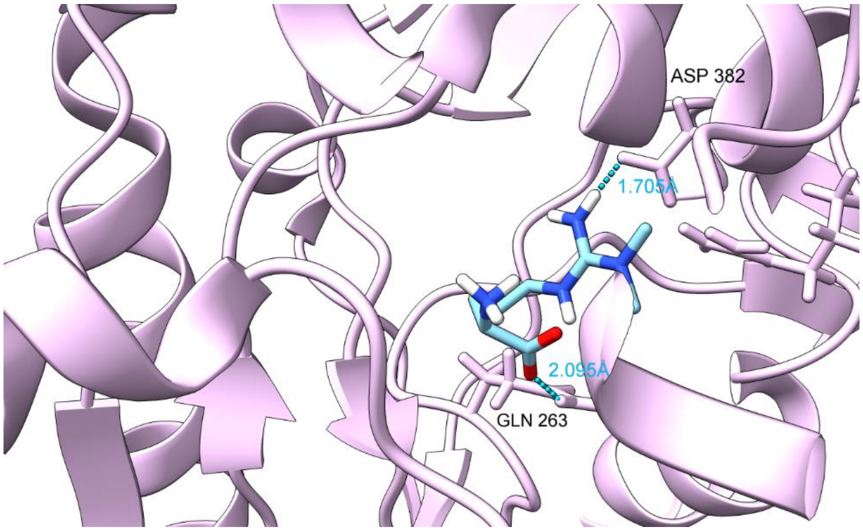

Figure S73. Predicted binding mode of ADMA in iNOS.

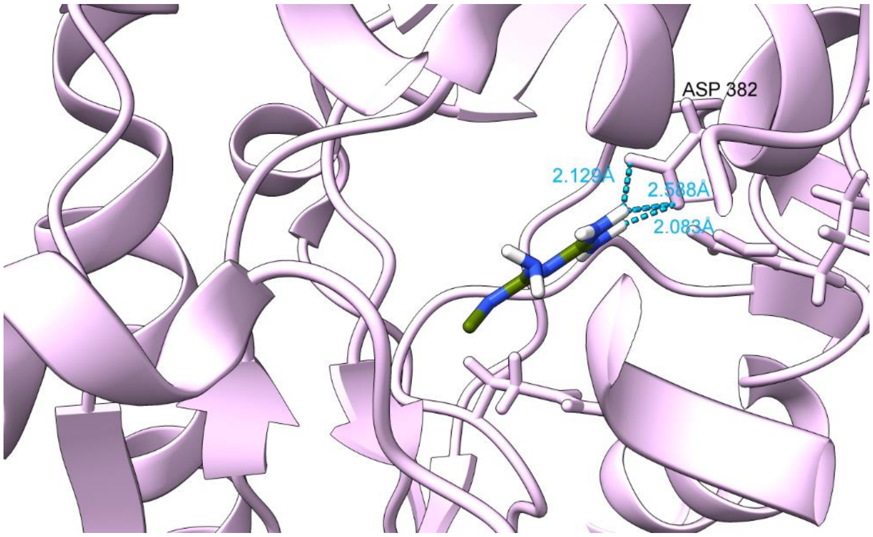

Figure S74. Predicted binding mode of metformin in iNOS.

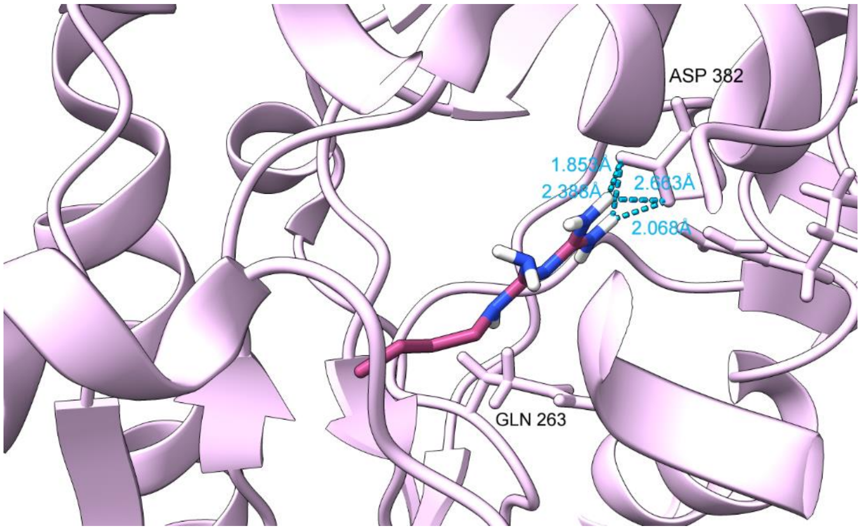

Figure S75. Predicted binding mode of buformin in iNOS.

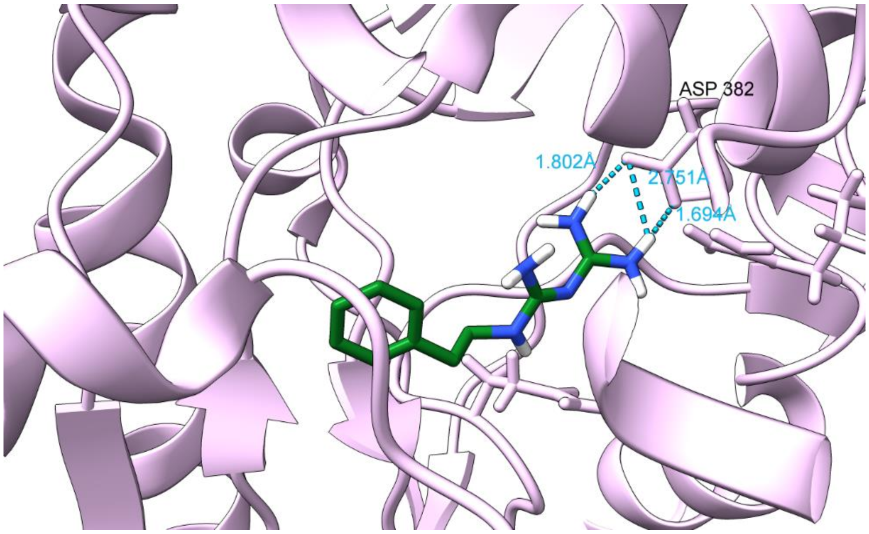

Figure S76. Predicted binding mode of phenformin in iNOS.

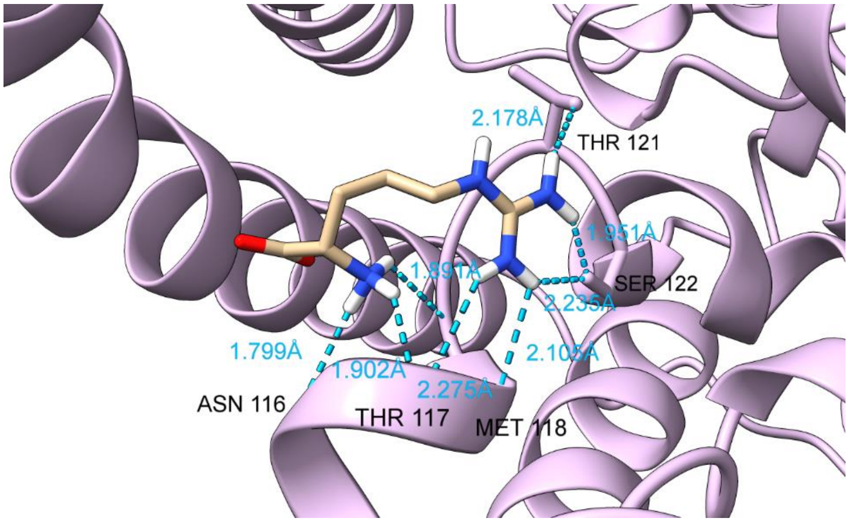

Figure S77. Predicted binding mode of L-arginine in SLC38A9.

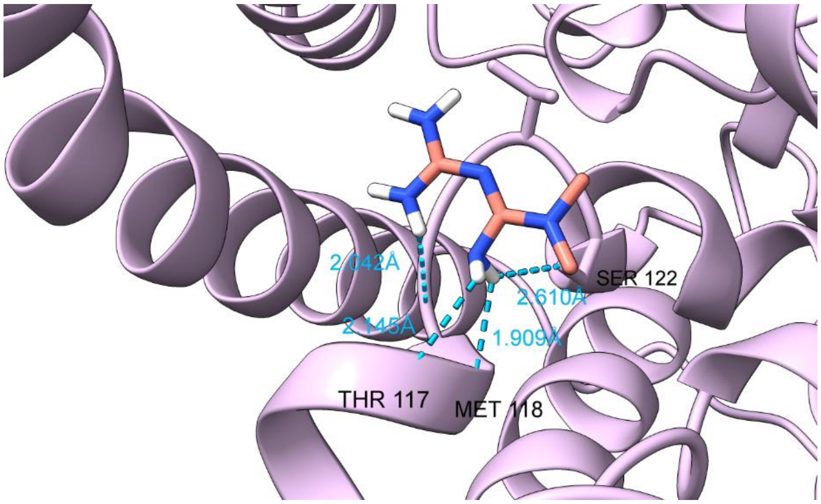

Figure S78. Predicted binding mode of metformin in SLC38A9.

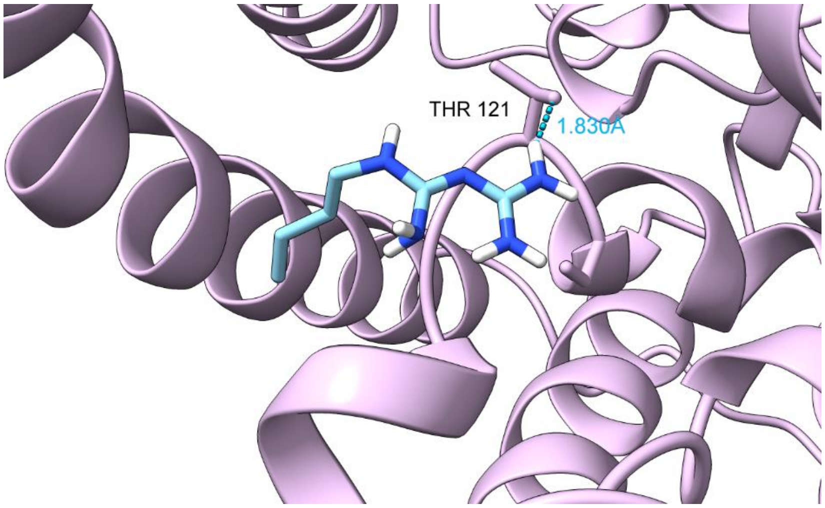

Figure S79. Predicted binding mode of buformin in SLC38A9.

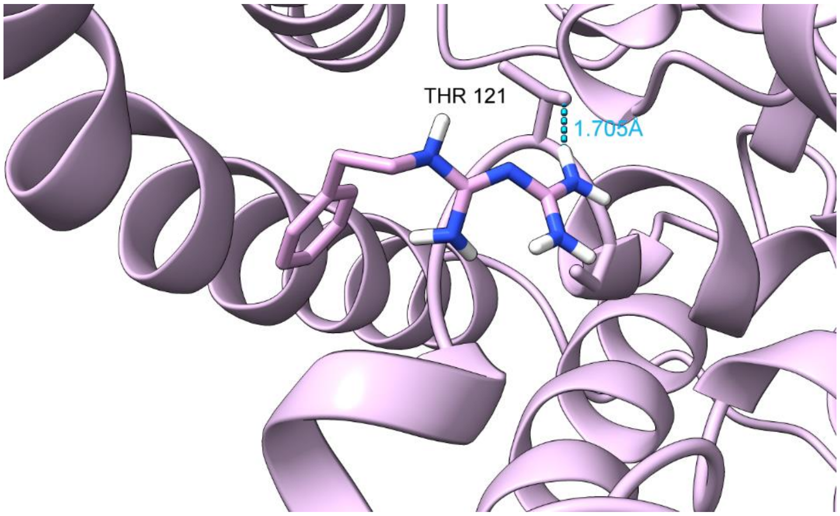

Figure S80. Predicted binding mode of phenformin in SLC38A9.

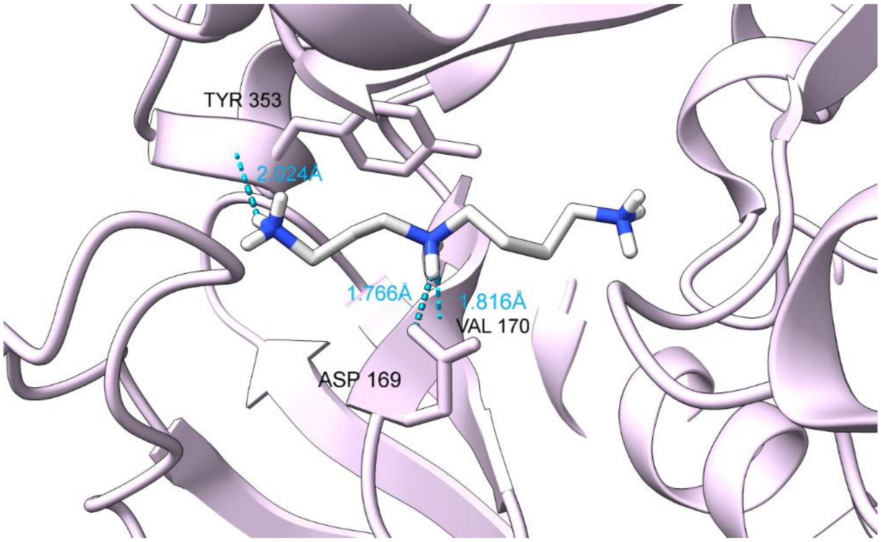

Figure S81. Predicted binding mode of spermidine in SPMS.

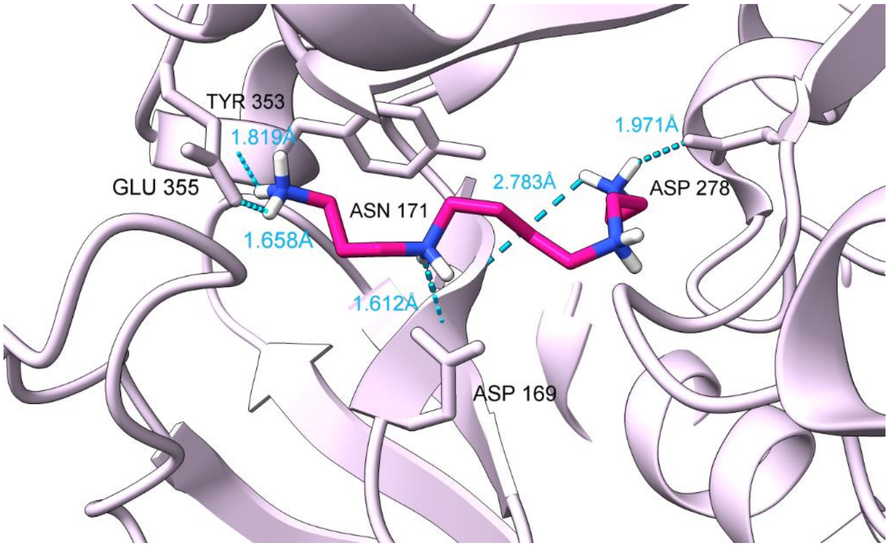

Figure S82. Predicted binding mode of spermine in SPMS.

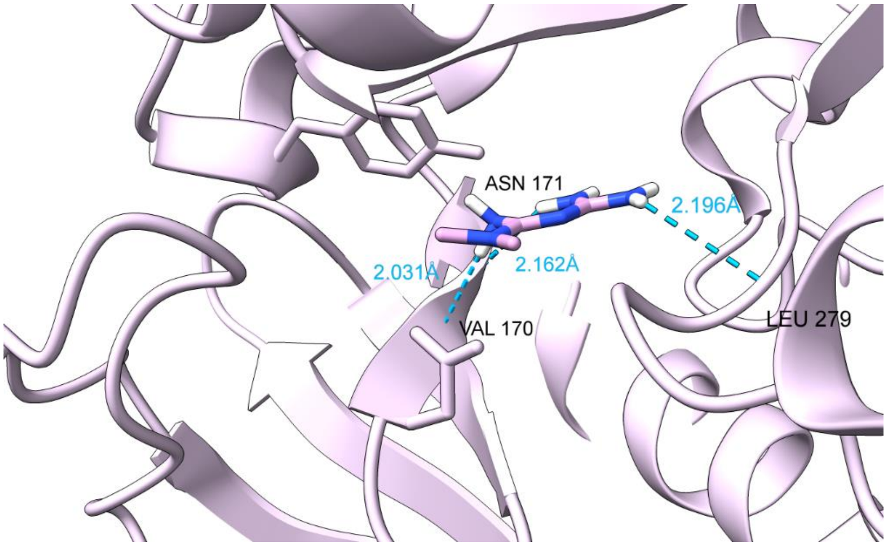

Figure S83. Predicted binding mode of metformin in SPMS.

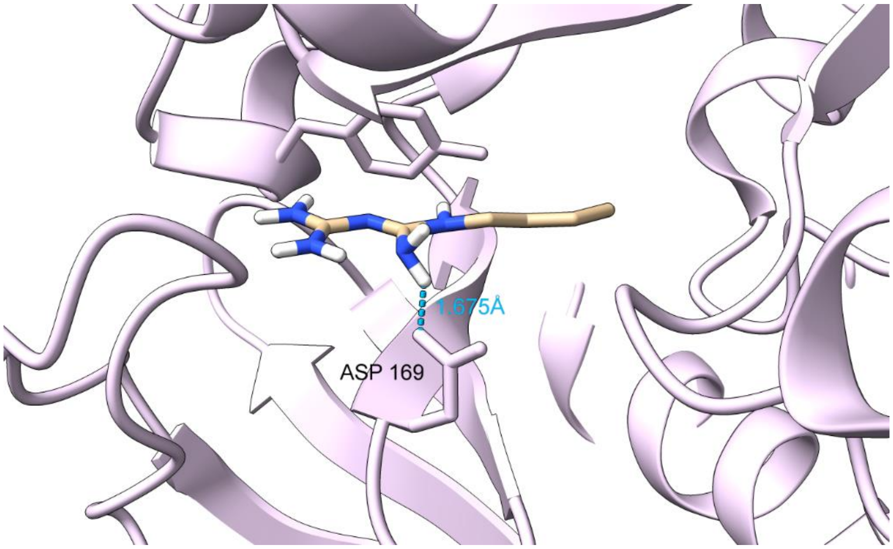

Figure S84. Predicted binding mode of buformin in SPMS.

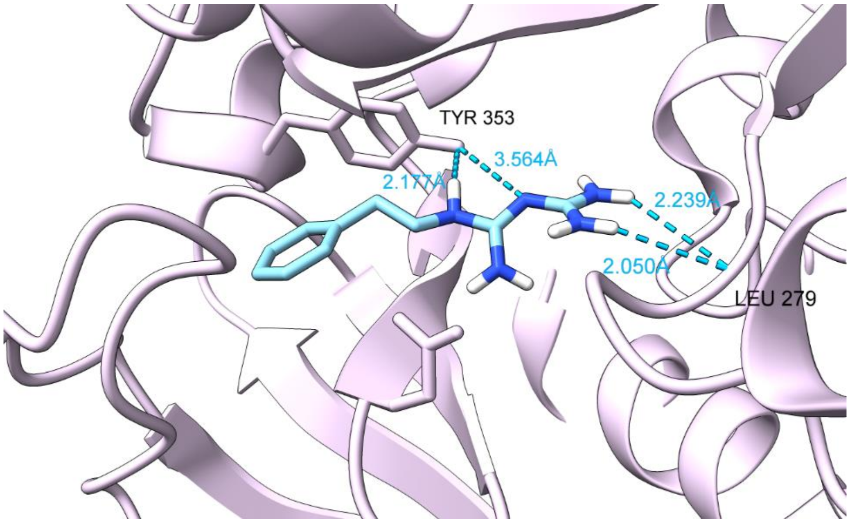

Figure S85. Predicted binding mode of phenformin in SPMS.

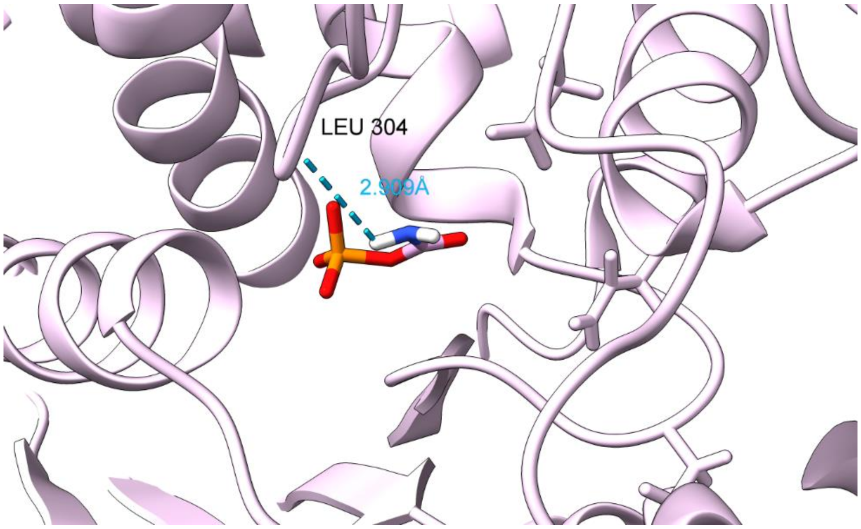

Figure S86. Predicted binding mode of carbamoyl phosphate in OTC.

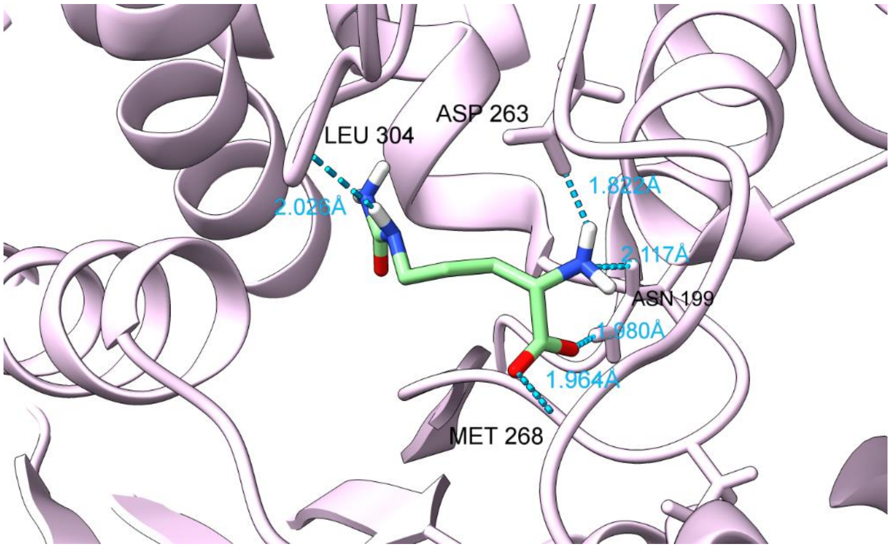

Figure S87. Predicted binding mode of L-citrulline in OTC.

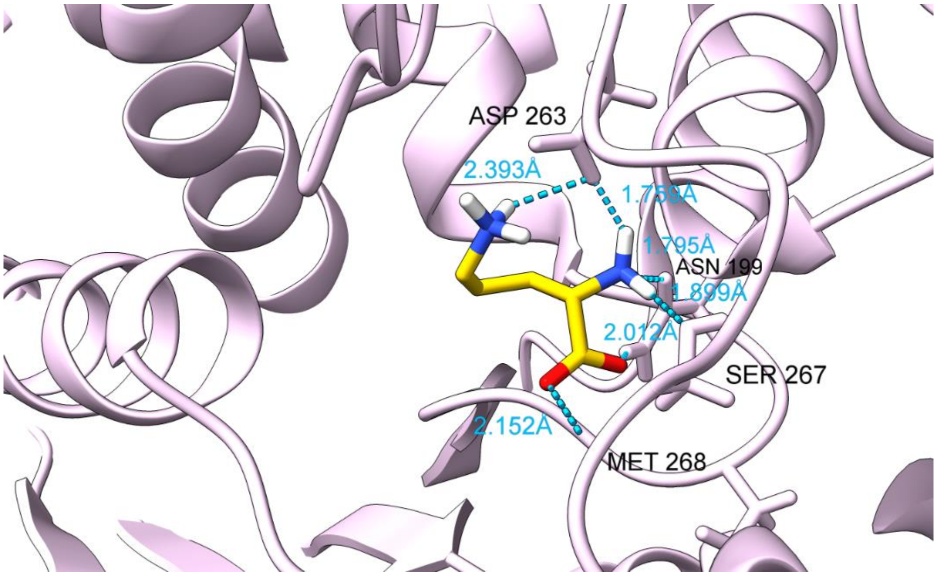

Figure S88. Predicted binding mode of L-ornithine in OTC.

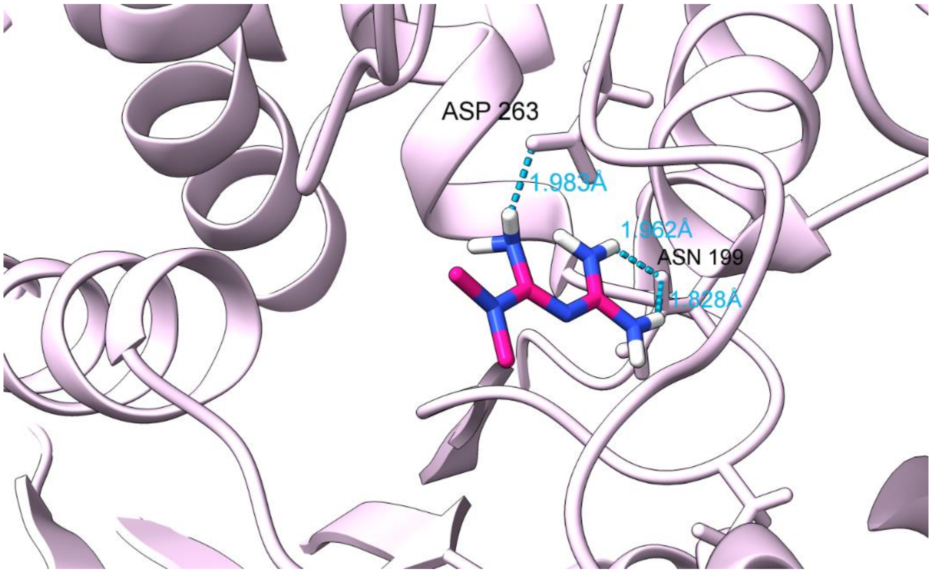

Figure S89. Predicted binding mode of metformin in OTC.

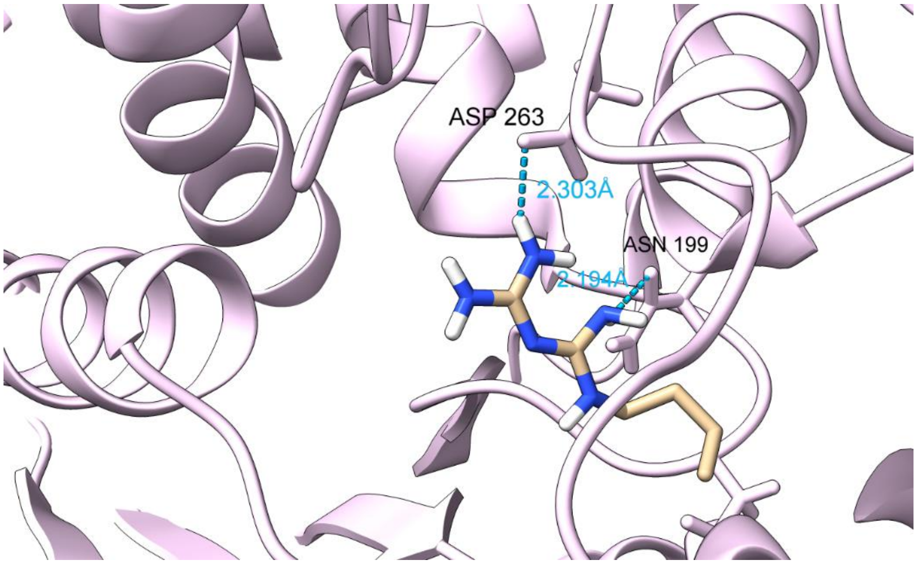

Figure S90. Predicted binding mode of buformin in OTC.

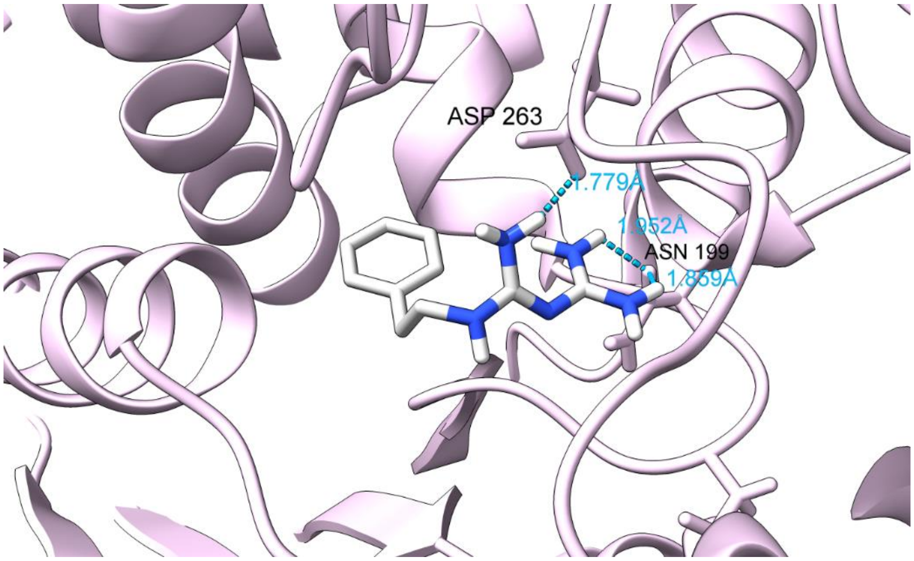

Figure S91. Predicted binding mode of phenformin in OTC.

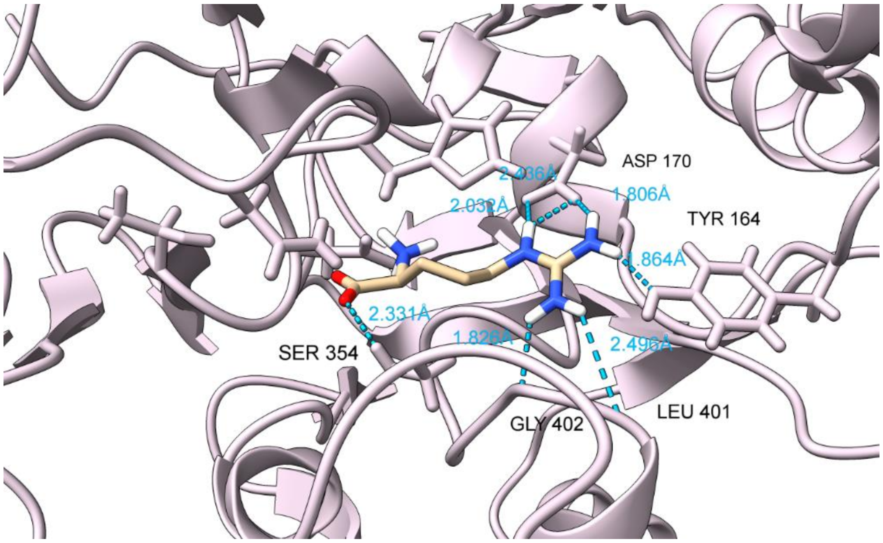

Figure S92. Predicted binding mode of L-arginine in AGAT.

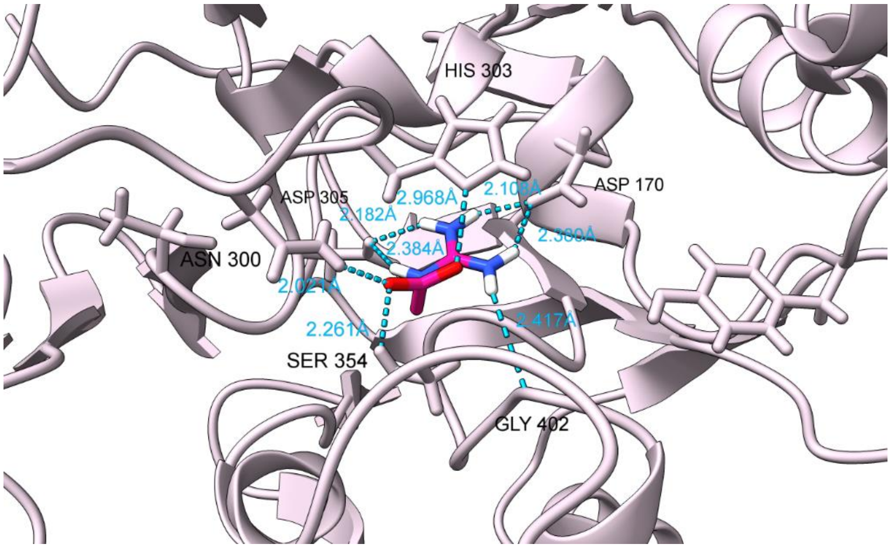

Figure S93. Predicted binding mode of guanidinoacetate in AGAT.

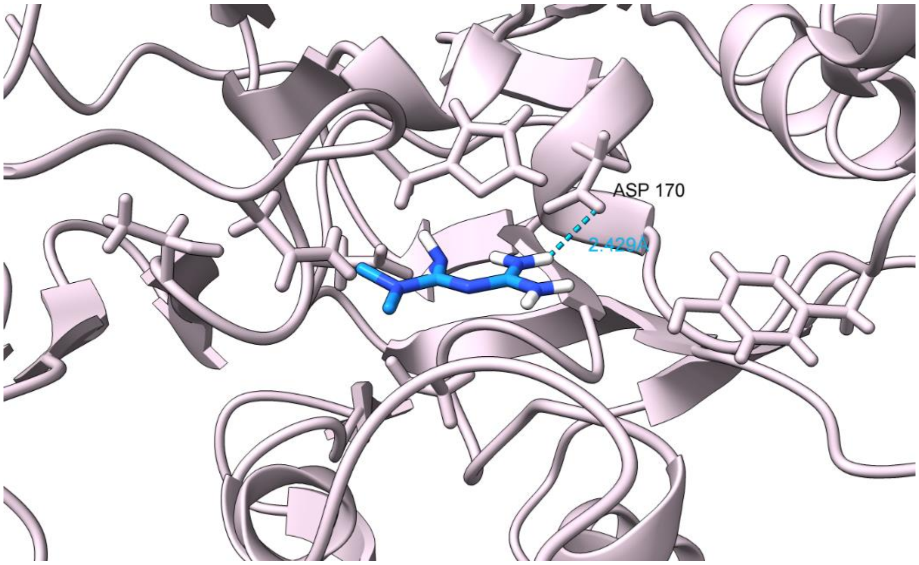

Figure S94. Predicted binding mode of metformin in AGAT.

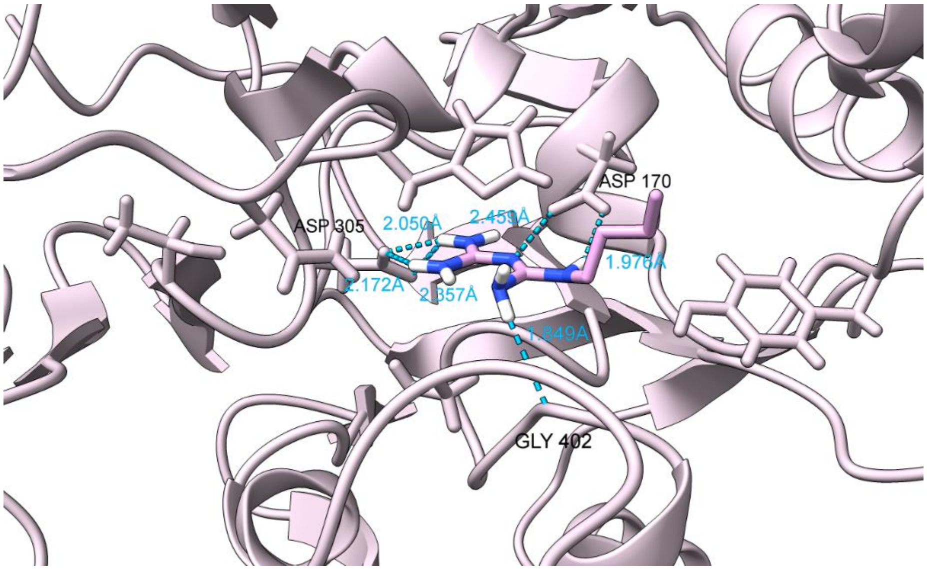

Figure S95. Predicted binding mode of buformin in AGAT.

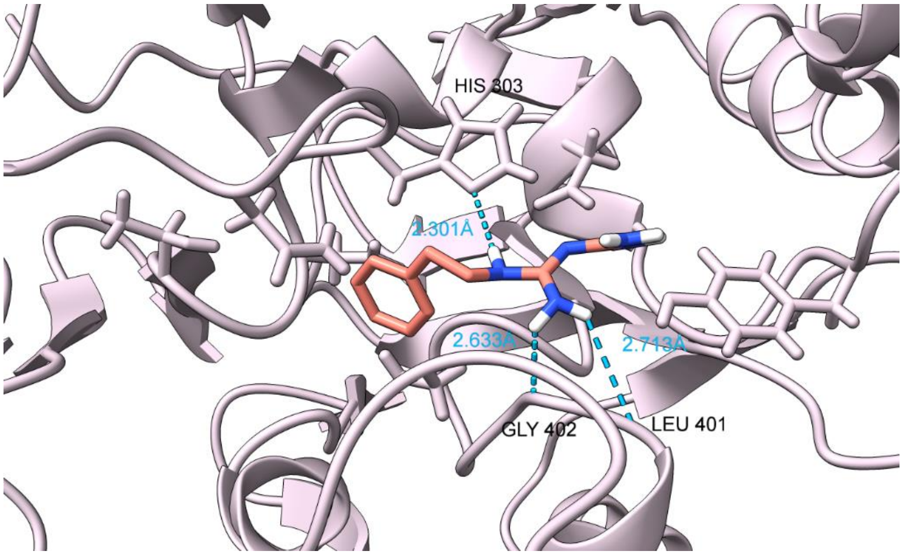

Figure S96. Predicted binding mode of phenformin in AGAT.

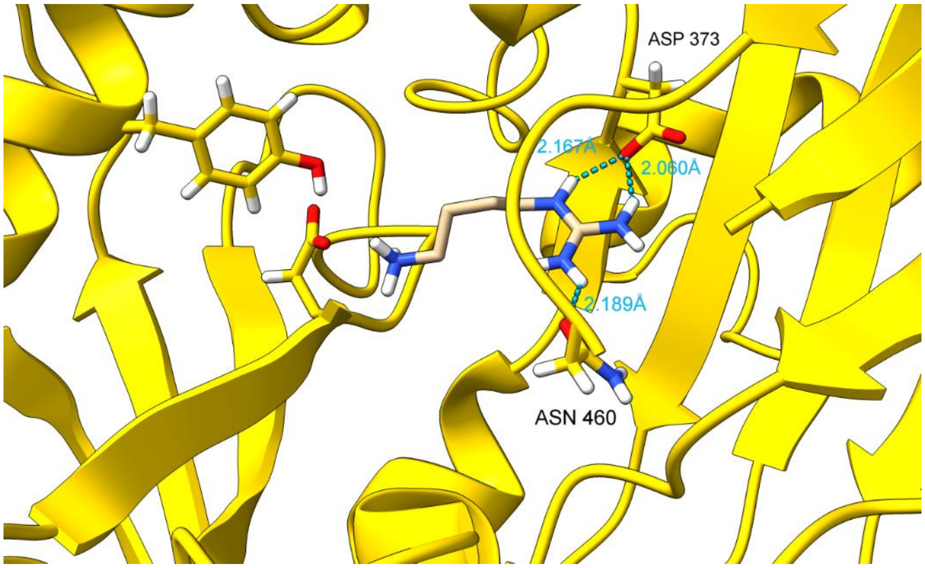

Figure S97. Predicted binding mode of agmatine in DAO.

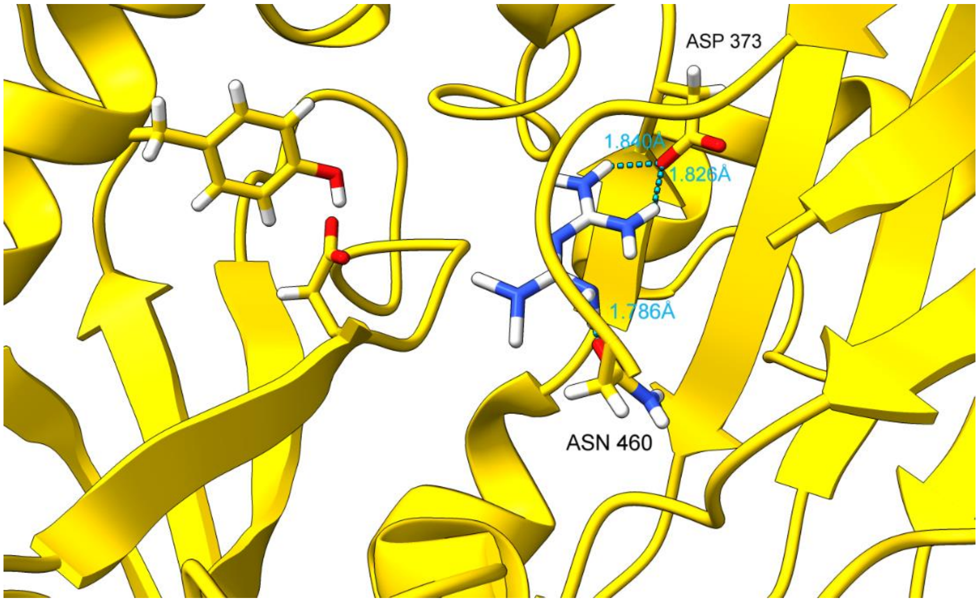

Figure S98. Predicted binding mode of metformin in DAO.

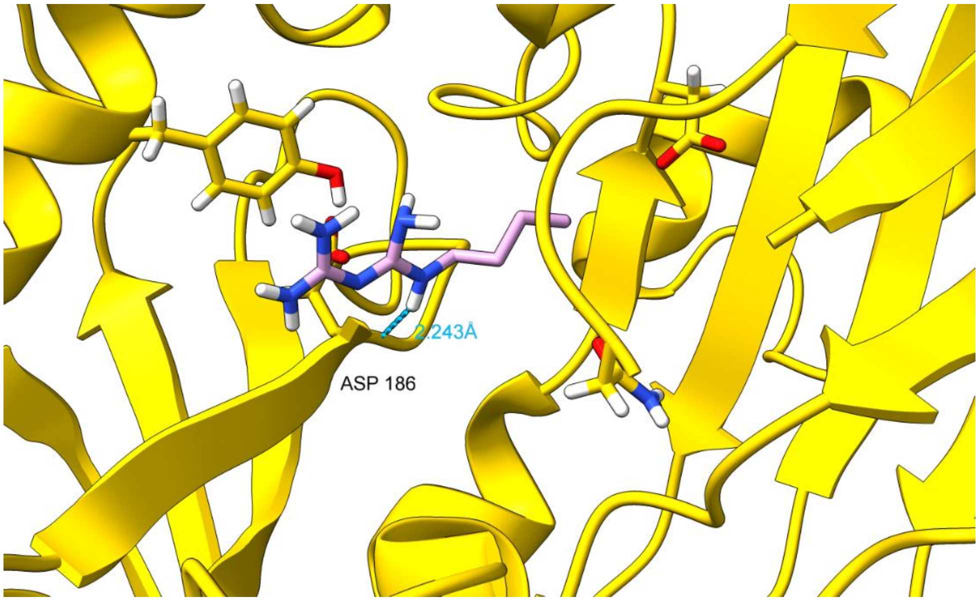

Figure S99. Predicted binding mode of buformin in DAO.

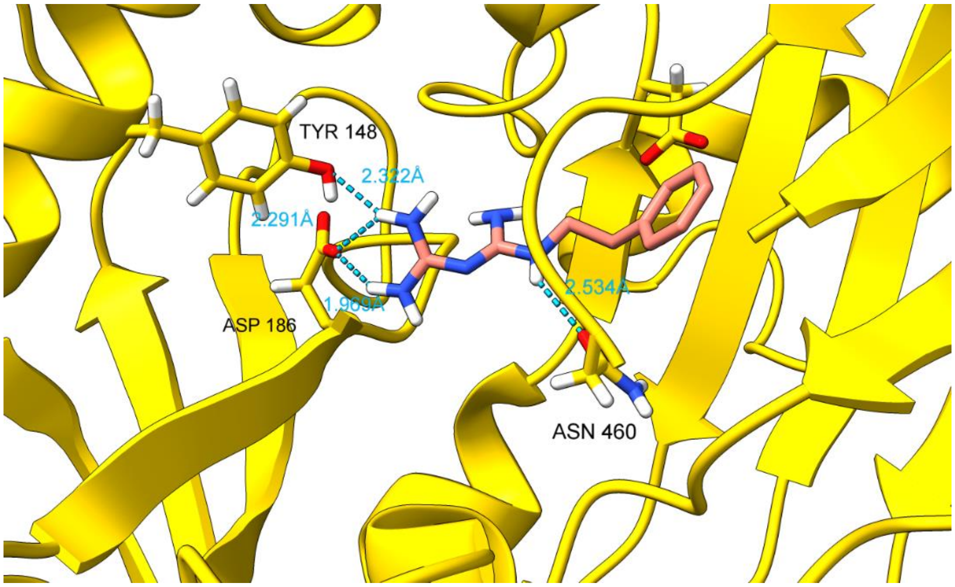

Figure S100. Predicted binding mode of phenformin in DAO.

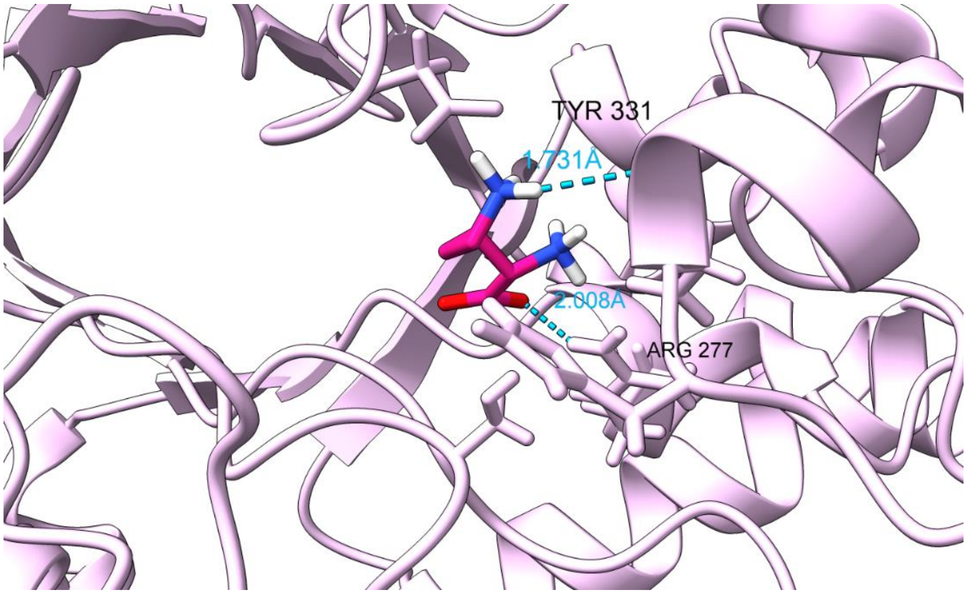

Figure S101. Predicted binding mode of L-ornithine in ODC.

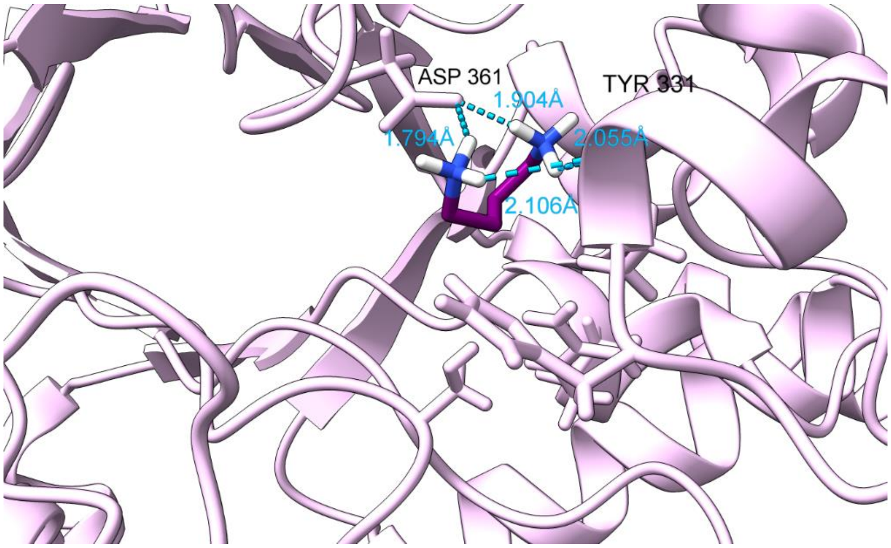

Figure S102. Predicted binding mode of putrescine in ODC.

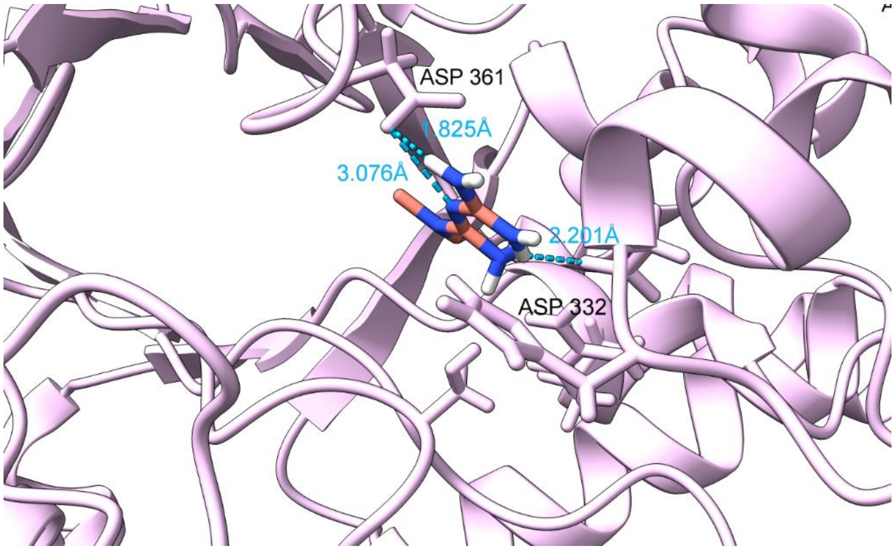

Figure S103. Predicted binding mode of metformin in ODC.

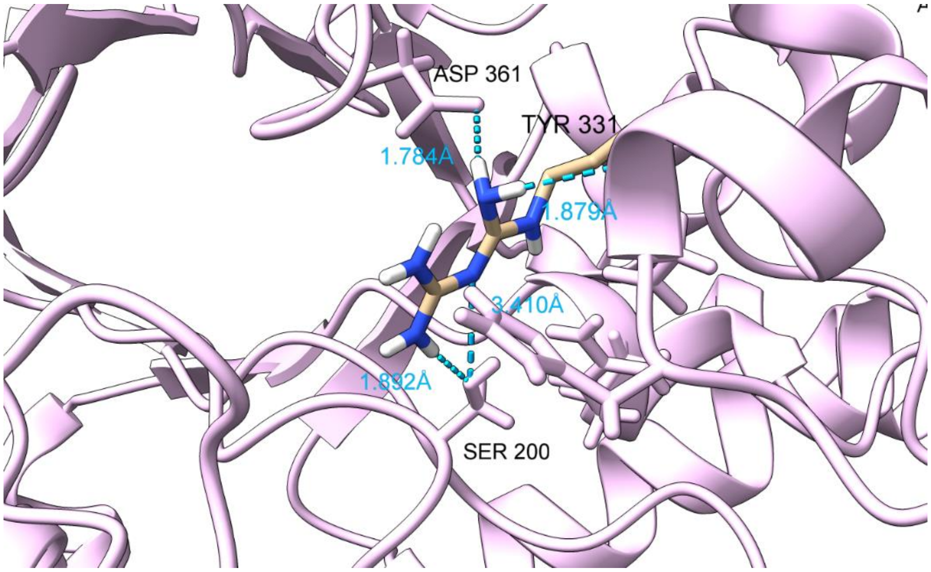

Figure S104. Predicted binding mode of buformin in ODC.

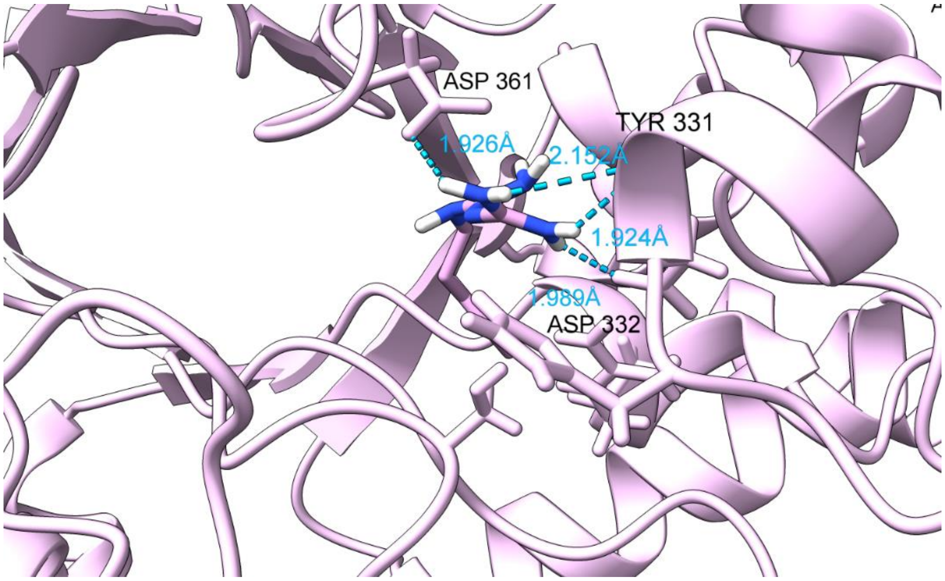

Figure S105. Predicted binding mode of phenformin in ODC.

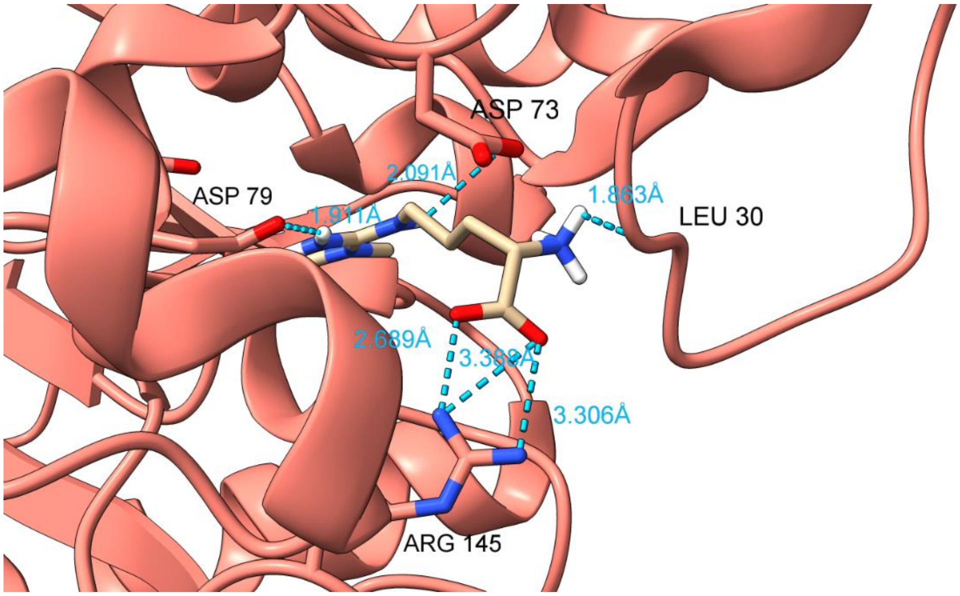

Figure S106. Predicted binding mode of ADMA in DDAH1.

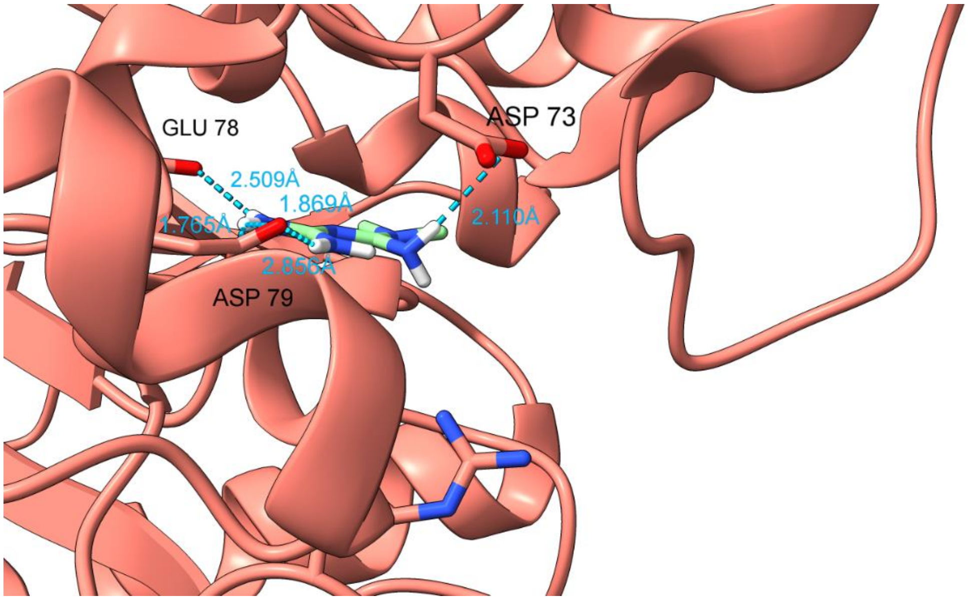

Figure S107. Predicted binding mode of metformin in DDAH1.

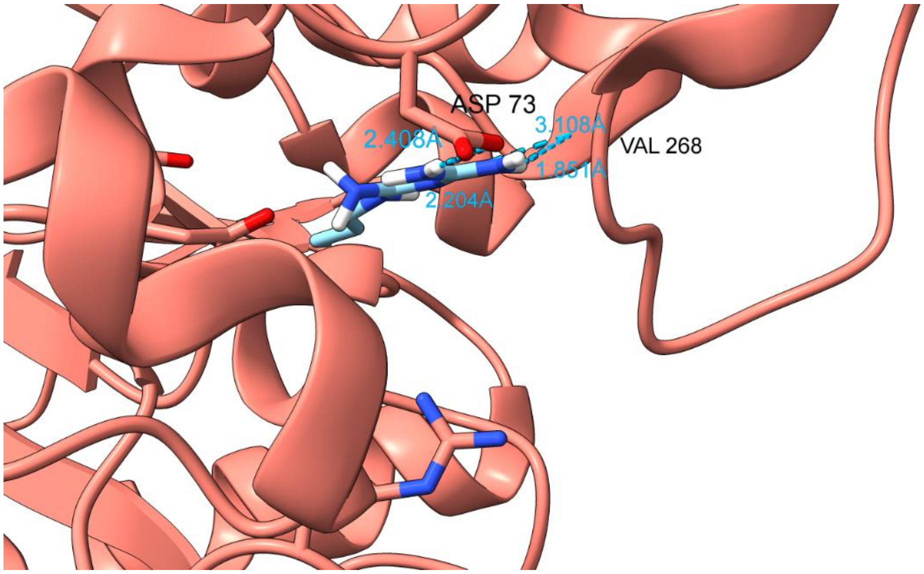

Figure S108. Predicted binding mode of buformin in DDAH1.

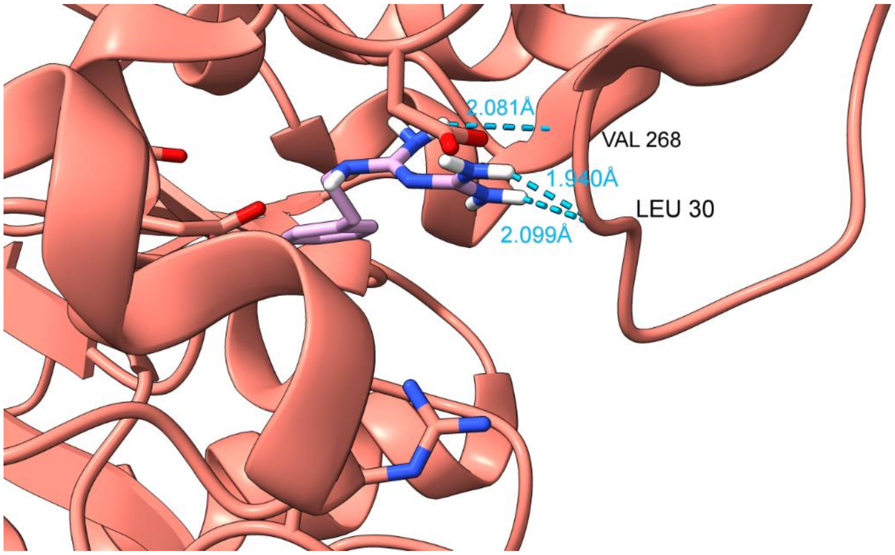

Figure S109. Predicted binding mode of phenformin in DDAH1.

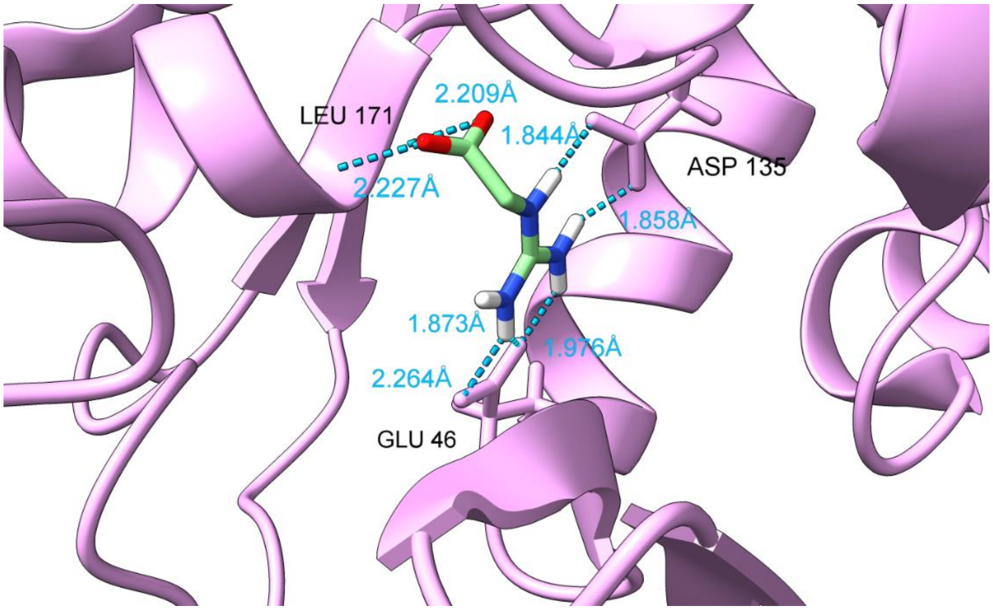

Figure S110. Predicted binding mode of guanidinoacetate in GAMT.

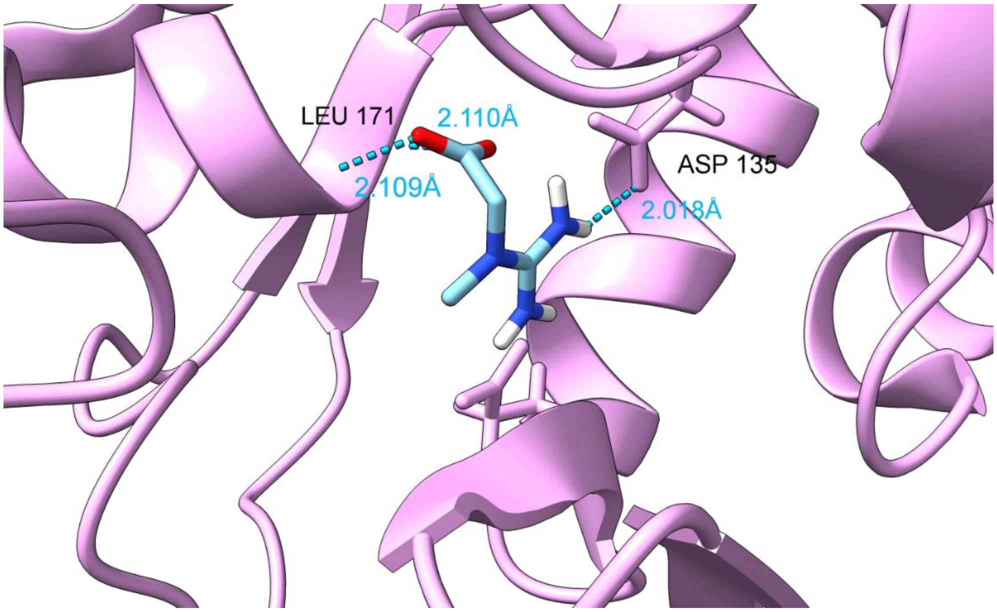

Figure S111. Predicted binding mode of creatine in GAMT.

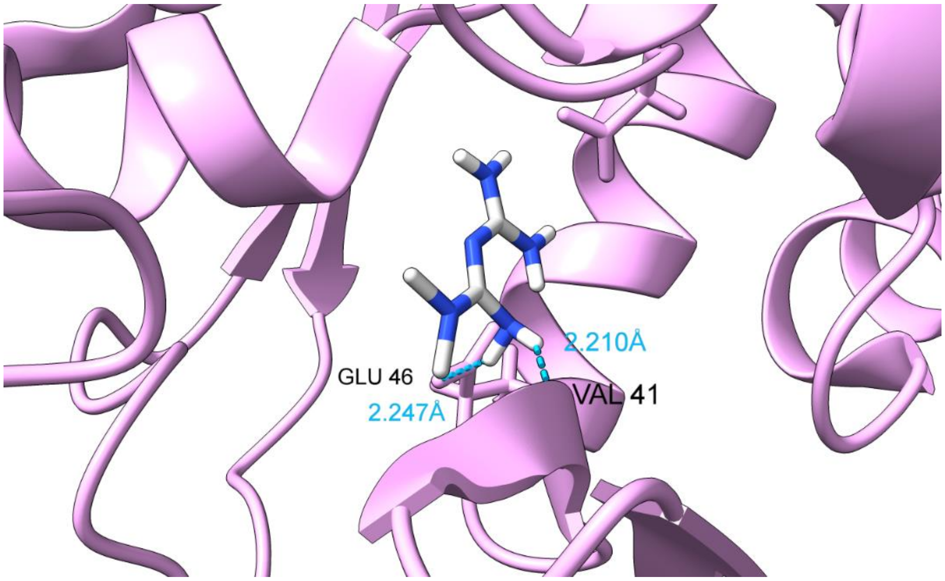

Figure S112. Predicted binding mode of metformin in GAMT.

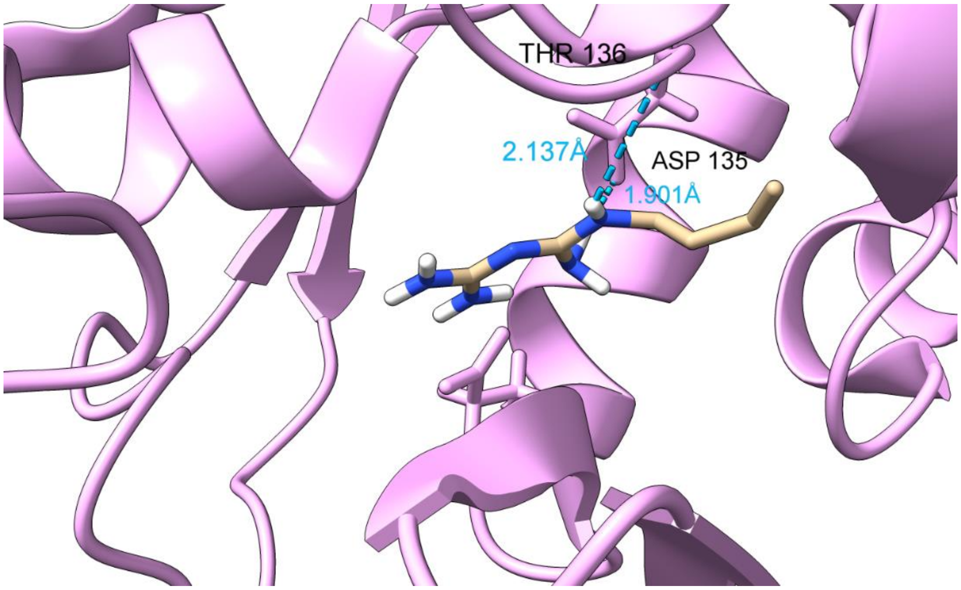

Figure S113. Predicted binding mode of buformin in GAMT.

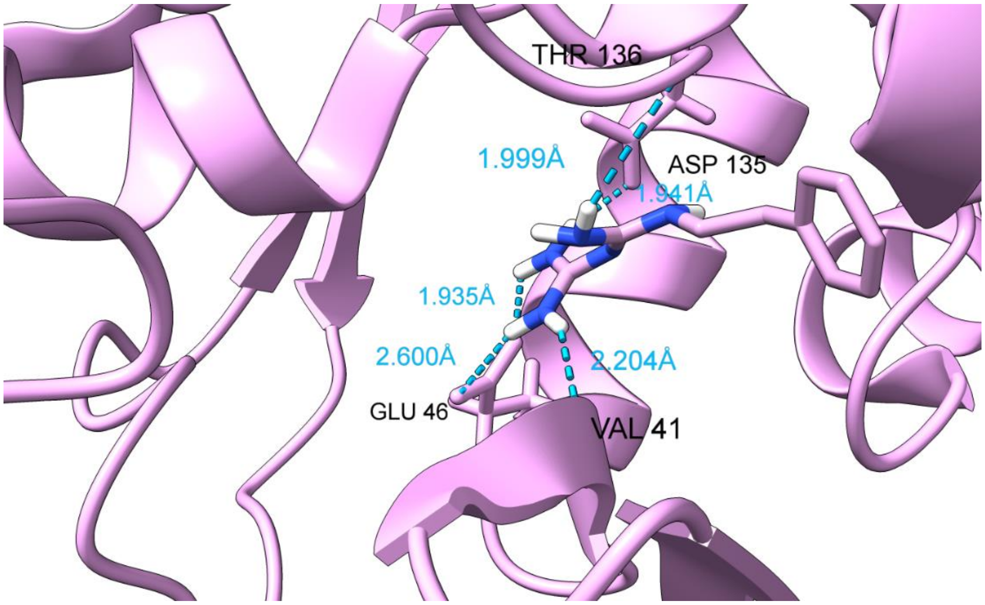

Figure S114. Predicted binding mode of phenformin in GAMT.
